# A new taxonomy was developed for overlap across 'overviews of systematic reviews': A meta‐research study of research waste

**DOI:** 10.1002/jrsm.1542

**Published:** 2022-01-23

**Authors:** Carole Lunny, Emma K. Reid, Trish Neelakant, Alyssa Chen, Jia He Zhang, Gavindeep Shinger, Adrienne Stevens, Sara Tasnim, Shadi Sadeghipouya, Stephen Adams, Yi Wen Zheng, Lester Lin, Pei Hsuan Yang, Manpreet Dosanjh, Peter Ngsee, Ursula Ellis, Beverley J. Shea, James M. Wright

**Affiliations:** ^1^ Cochrane Hypertension Review Group, Therapeutics Initiative, Department of Anesthesiology, Pharmacology & Therapeutics, Faculty of Medicine University of British Columbia Vancouver British Columbia Canada; ^2^ Nova Scotia Health Halifax Nova Scotia Canada; ^3^ Royal College of Surgeons Ireland; ^4^ Faculty of Pharmaceutical Science University of British Columbia Vancouver British Columbia Canada; ^5^ Michael G. DeGroote Cochrane Canada Centre, Department of Health Research Methods, Evidence, and Impact McMaster University Ontario Canada; ^6^ Woodward Library University of British Columbia Vancouver British Columbia Canada; ^7^ Clinical Epidemiology Program Ottawa Hospital Research Institute, University of Ottawa Ontario Canada

**Keywords:** duplication, meta‐reviews, overlap, overview of systematic reviews, overviews of reviews, ‘reviews of reviews’, redundancy, umbrella reviews, waste

## Abstract

Multiple ‘overviews of reviews’ conducted on the same topic (“overlapping overviews”) represent a waste of research resources and can confuse clinicians making decisions amongst competing treatments. We aimed to assess the frequency and characteristics of overlapping overviews. MEDLINE, Epistemonikos and Cochrane Database of Systematic Reviews were searched for overviews that: synthesized reviews of health interventions and conducted systematic searches. Overlap was defined as: duplication of PICO eligibility criteria, and not reported as an update nor a replication. We categorized overview topics according to 22 WHO ICD‐10 medical classifications, overviews as broad or narrow in scope, and overlap as identical, nearly identical, partial, or subsumed. Subsummation was defined as when broad overviews subsumed the populations, interventions and at least one outcome of another overview. Of 541 overviews included, 169 (31%) overlapped across similar PICO, fell within 13 WHO ICD‐10 medical classifications, and 62 topics. 148/169 (88%) overlapping overviews were broad in scope. Fifteen overviews were classified as having nearly identical overlap (9%); 123 partial overlap (73%), and 31 subsumed (18%) others. One third of overviews overlapped in content and a majority covered broad topic areas. A multiplicity of overviews on the same topic adds to the ongoing waste of research resources, time, and effort across medical disciplines. Authors of overviews can use this study and the sample of overviews to identify gaps in the evidence for future analysis, and topics that are already studied, which do not need to be duplicated.

## 1 BACKGROUND

Rigorous ‘overviews of systematic reviews’ (henceforth called overviews) and high quality ‘systematic reviews with or without meta‐analysis’ give the best perspective of our current state of evidence on a subject. Overviews synthetize the results of multiple systematic reviews and help inform evidence‐based clinical practice. Overviews, also called umbrella reviews, meta‐reviews, or reviews of reviews, are one of the multiple types of evidence syntheses. They are growing in number and popularity, and our bibliometric study of the prevalence of overviews found an 8‐fold increase in the number of overviews published between 2009 (*n* = 25) and 2020 (*n* = 332).^1^ The growth in overviews is unlikely to decrease.^1^

Several author teams have expressed concerns about the volume of systematic reviews published which are overlapping in content.^2^, ^3^, ^4^ Conflicting results or conclusions across systematic reviews on the same topic can confuse or create uncertainty for policymakers and clinicians who are required to choose amongst all competing treatments, and may impact and delay clinical decision‐making.^5^ These concerns can also be extrapolated to overviews reporting discordant findings. Another downside to the publication of multiple overviews on the same topic is that the efforts of the investigators, journal editors and peer reviewers may be unnecessarily duplicated.

Overviews have the potential for overlap because many are broad in scope, thus covering several individual topics. These broad overviews often address less specific questions than their constituent systematic reviews, including a wider range of study populations and conditions, interventions, and contexts. Overviews that are broad in scope allow for policy relevance; for example, overviews have informed clinical practice guidelines (e.g., Zhang, 2007^5^) and government health policies (e.g., Australian Government Department of Health, 2015).^6^ Broad overviews can make a large volume of evidence accessible to clinicians and policymakers,^7^ but may necessitate extensive screening, data extraction, and the synthesis of a large number of systematic reviews.

Overviews may alternatively aim to answer narrow, focused clinical, public health, and policy questions, and to identify and explore reasons for variation in the results, interpretation, or conclusions of systematic review analyses.^8^, ^9^, ^10^, ^11^, ^12^, ^13^, ^14^, ^15^, ^16^, ^17^ As an example, an overview that compared surgical versus conservative treatments for clavicle fractures aimed to determine which systematic review provided the most trustworthy evidence for treatment, and explored reasons for differences in review‐level results.^15^ Overviews with narrowly focused questions can be completed more quickly as compared with broad questions, but may have limited generalizability to different populations and settings.^18^

Research is needed to establish if and how overviews overlap in content. For example, overviews may be performed on unique topics which only partially overlap; they may represent updates of previous overviews done by the same team of authors (similar to updated Cochrane reviews); replications; or may be redundant/salami slicing publications on the same topic.^19^

This paper is the second of two companion papers. The first paper evaluated the bibliometric characteristics of overviews, and factors affecting citation rates and journal impact factors.^20^ In this second paper, we aimed to determine if the overviews overlap in eligibility criteria according to their populations and interventions and at least one outcome, henceforth referred to as overlapping overviews. In a post hoc analysis, we also aimed to categorize overviews as being narrow or broad in scope, and classify the overlap into four categories: partial, nearly identical, identical, or subsumed.

## 2 METHODS

### 2.1 Study design

This is a meta‐research study, which aims to evaluate research practices.^21^ We followed systematic review guidance for the searching, study selection, and data extraction stages of our study.^22^

### 2.2 Eligibility criteria

As described in our first companion paper,^1^ we performed a bibliometric study of overviews published from 2000–2018.

#### 2.2.1 Inclusion criteria for overviews including systematic reviews

- Synthesizes systematic reviews with or without meta‐analyses (but the overview may also include primary studies) as a primary focus.
- Searches the literature systematically, and with a search strategy section found in the main body of the paper (i.e., search strategy includes text words and MeSH terms in at least two databases).
- Methods section located in the main manuscript or within a supplementary file (not just in the abstract).
- Focuses on the effects of health interventions (e.g., clinical treatments like medication or therapies).

We excluded overviews that based their results exclusively on primary studies and methodological studies. Reports that were editorials, letters, or comments were excluded. Overviews of risk, exposure, prevention, measurement instruments, quality indicators, diagnostic, screening, or prognostic research were also excluded. We excluded protocols of overviews.

We included overviews published in any language and published from January 1, 2000 to December 31, 2018. Given that the Cochrane Handbook for Systematic Reviews of Interventions chapter on overviews was first published in 2009, we did not expect to identify overviews published prior to 2000. Reports were translated by one of the authors (French, Spanish, German, Mandarin), when needed.

We reasoned that overview may exist as a stand‐alone report or also packaged as part of a clinical practice guideline and health technology assessment; accordingly, we developed eligibility criteria for both circumstances.

#### 2.2.2 Inclusion criteria for clinical practice guidelines and health technology assessments (HTAs)

- Clinical practice guidelines or HTAs aim to primarily include, synthesize and present the results of the systematic reviews; but may also include additional primary studies.

#### 2.2.3 Inclusion criteria for overlapping overviews

We determined two or more overviews were overlapping if their eligibility criteria included the same population(s) or condition(s), intervention(s), and at least one outcome (PICO). When reported, we did not consider an update by the same authors, or if the authors stated the overview was a replication, as an overlapping overview.

### 2.3 Search

Overviews were retrieved using a validated search filter^23^ from MEDLINE (Ovid), Epistemonikos and the Cochrane Database of Systematic Reviews (CDSR) from 2000 to 2018 (Appendix A). The Epistemonikos search was limited to the “Broad Syntheses” category, which includes overviews of systematic reviews, HTAs and clinical practice guidelines.

### 2.4 Overview screening and study selection

The initial search results were imported into Excel 2010 for screening. A pilot screening of the first 19 papers was conducted in duplicate by all screeners to ensure high levels of agreement and common definitions of coding. We screened the titles and abstracts against the stated eligibility criteria first, then eligible full‐text articles were reviewed for inclusion. Two independent reviewers screened reports at the title and abstract, and then again at the full text stage, then compared their results. Discrepancies were resolved by consensus, and arbitration by a third reviewer when necessary.

In two previously published studies categorizing all methods used in overviews (2013 and 2016),^23^, ^24^ 187 overviews were screened using identical methods (Appendix A). We therefore included these 187 studies and categorized them as “other sources”.

### 2.5 Data extraction

Data extraction was piloted on 20 studies by all authors independently to identify any missing variables, come to agreement on coding definitions, and refine/reword the items. Discrepancies in the piloting phase were discussed and consensus reached by two authors. Full data extraction was performed independently by one investigator and checked by a second reviewer.

We categorized the medical classification of each overview using the 10th revision of the International Statistical Classification of Diseases and Related Health Problems (ICD), a medical classification list by the World Health Organization (WHO) (https://www.who.int/standards/classifications/classification-of-diseases). The main condition or intervention in the title of the overview was entered into the search function of the WHO ICD‐10 site (https://icd.who.int/browse10/2016/en) to determine its classification. For example, the title “Nonpharmacological *treatment for behavioral and psychological disturbances* in older adults with dementia” was categorized in the ICD‐10 classification “Mental and behavioral disorders” because the intervention was treatment for behavioral disturbance in a dementia population. In addition to ICD‐10 classification, the topic of each overview was determined through review of the eligibility criteria, including study population(s), intervention(s), and outcome(s).

For overlapping overviews, we additionally extracted whether an author was involved in more than one overview on the same topic. We hypothesized that if an overview was conducted by the “same team of authors” or had some authors in common, then it might be reported as an update of a previous overview, or might represent two forms of self‐plagiarism: (a) redundant (duplicate) publication, or (b) salami slicing or a salami publication.^19^ Redundant/duplicate publication can be defined as a reporting identical or very similar data in two or more papers without explicitly stating that the data are recycled.^19^ Salami publication, aka salami slicing, is a form of redundant publication where different papers from the same data set are published. We also extracted the journal of publication, number of included systematic reviews, search date, inclusion of meta‐analysis, and funding status.

### 2.6 Classification of overviews as broad or narrow in scope

**FIGURE 1:**
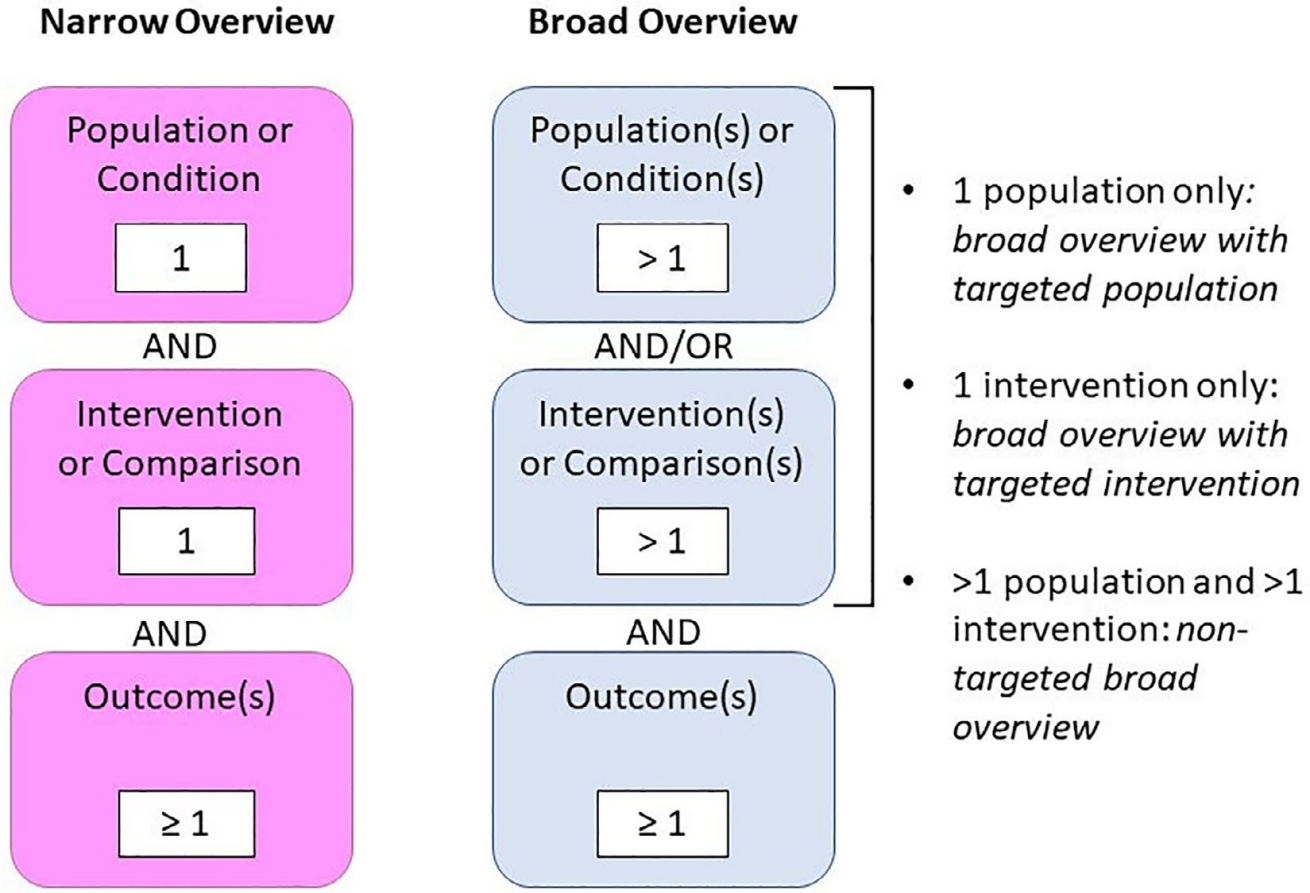
Classification of broad and narrow overviews [Colour figure can be viewed at wileyonlinelibrary.com]

In a post hoc analysis, we classified overviews as being broad or narrow in scope (Figure 1). We hypothesized that broad overviews were more prevalent than overviews with a narrow scope. We defined a broad overview as addressing: (a) more than one distinct population (e.g., individuals with cancer, menopause, and lower back pain) or a generalized population (e.g., humans of all ages), and/or (b) multiple interventions (e.g., aerobic exercise, resistance training) for outcomes of interest. Broad overviews could therefore be further sub‐classified as being non‐targeted (multiple populations and interventions), as having a targeted population (with multiple interventions), or as having a targeted intervention (for multiple populations). We defined narrow overviews as covering only one population and one intervention or comparison (e.g., aripiprazole for patients with schizophrenia).

The scope of outcomes within overviews was not specifically addressed within the broad/narrow classification system in this study. We did not consider the broad or narrow scope of the overviews to mean a broad or narrow scope of the evidence but rather intended this system to be a means to characterize the breadth of the topic investigated.

This schema provides a general system to classify the scope of overviews and can be applied to overviews with or without topic overlap. When specifically assessing how two or more overviews overlap, the schema is helpful as a first step from which the overlap type can be more easily determined.

### 2.7 Classification of overlapping overviews

We classified the overlap across overviews based on the degree of similarity of their PICO eligibility criteria elements. We classified the overlapping overviews into four categories:

- *Identical overlap* occurred when an overview's eligible populations, interventions, comparisons, and all outcomes were identical to another overview. Identical overviews had to have the same aims and include the same study designs.
- *Nearly identical overlap* was when an overview's eligible populations, interventions, comparisons, and *at least one* outcome were identical to another. Nearly identical may have included overviews with the same PICO elements but allowed for inclusion of different study designs.
- *Partial overlap* was defined as two or more overviews with at least one component of each of their populations, interventions, and comparisons in common, along with at least one common outcome (example in Section 3.7).
- *Subsumed overlap* was when the full scope of populations, interventions, comparisons and at least one outcome in an overview was addressed in full by a second (broader) overlapping overview.

A broad overview that subsumed another overview (broad or narrow) was classified as having partial overlap with the overview it encompassed, and the overview encompassed by the broader overview was considered subsumed. One overview could be classified in multiple categories depending on the number of overviews it overlapped with and the nature of the overlap (e.g., Wells et al.^25^ is subsumed by Geneen^26^; and has partial overlap with Swinkels^27^). Two authors independently coded the overlapping overviews which was then checked by a second reviewer.

### 2.8 Data analysis

Descriptive analysis using frequencies and percentages were performed for categorical data and median and interquartile range (IQR) for continuous data.

The distribution of total overviews by medical classification was plotted in a bubble chart using Excel. The x‐axis represents medical classification, y‐axis the number of overviews pertaining to that medical classification and the size of the bubble (third variable) represents the cumulative number of systematic reviews in all overviews included in that classification. We described the gaps in ICD‐10 medical classifications covered by all overviews published between 2000 and 2018.

## 3 RESULTS

### 3.1 Search results

After searching MEDLINE, CDSR, Epistemonikos and other sources, we retrieved 10,145 records (Appendix B Figure). After removal of duplicates, 8220 records remained, 6733 were excluded at the title/abstract stage, and 946 were excluded at the full text stage. A total of 541 overviews published between 2000 and 2018 were included (Appendix C). Many of the citations were excluded because they did not have a methods section, did not conduct a systematic search, and did not search for and include systematic reviews (Appendix B Figure). For example, of the 873 citations that might have been included as overviews (as they searched for, and included systematic reviews/meta‐analyses, guidelines or HTAs), 122 (14%) did not contain a systematic search strategy.

### 3.2 WHO ICD‐10 medical classifications

**FIGURE 2:**
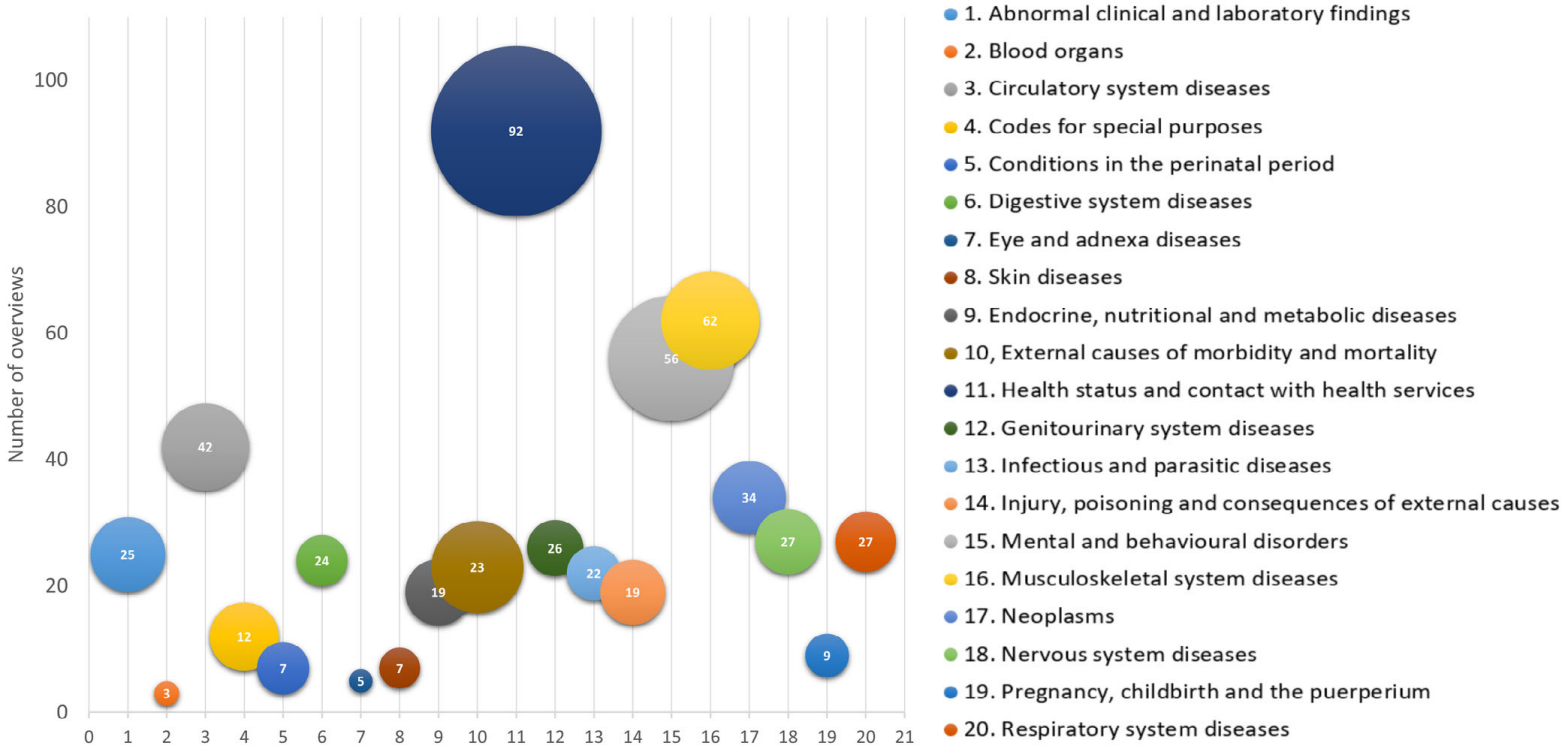
Bubble chart of WHO ICD‐10 medical classifications and overview frequency. Bubble chart depicts the ICD‐10 medical classifications by color, the y‐axis is the number of overviews, the x‐axis is the 20 WHO ICD‐10 medical classifications, and the size of the bubble (third variable) represents the cumulative number of systematic reviews included in the overviews. For example, the most prominent bubble is dark blue in the top center covering the most frequent ICD‐10 classification “Factors influencing health status and contact with health services” (92/541 [17%]) [Colour figure can be viewed at wileyonlinelibrary.com]

The 541 overviews covered 20 of the 22 WHO ICD‐10 medical classifications (Figure 2). The most frequent ICD‐10 classification for retrieved overviews (92/541 [17%]) was “factors influencing health status and contact with health services.” Another 62/541 (11.5%) focused on diseases of the musculoskeletal system and connective tissue, 56/541 (10.4%) were about mental and behavioral disorders, 42/541 (7.8%) were on diseases of the circulatory system, and 34/541 (6.3%) were focused on neoplasms (Figure 2). A little under half of the overviews focused on 15 other ICD‐10 medical classification (Table 1).

**TABLE 1:** Frequency of WHO ICD‐10 medical classifications in 541 overviews

WHO ICD‐10 medical classifications	Frequency of overviews	Percent (%)
Blood and blood‐forming organs	3	0.6
Certain conditions originating in the perinatal period	7	1.3
Certain infectious and parasitic diseases	22	4.1
Codes for special purposes	12	2.2
Congenital malformations, deformations, and chromosomal abnormalities	0	0.0
Diseases of the circulatory system	42	7.8
Diseases of the digestive system	24	4.4
Diseases of the ear and mastoid process	0	0.0
Diseases of the eye and adnexa	5	0.9
Diseases of the genitourinary system	26	4.8
Diseases of the musculoskeletal system and connective tissue	62	11.5
Diseases of the nervous system	27	5.0
Diseases of the respiratory system	27	5.0
Diseases of the skin and subcutaneous tissue	7	1.3
Endocrine, nutritional, and metabolic diseases	19	3.5
External causes of morbidity and mortality	23	4.3
Factors influencing health status and contact with health services	92	17.0
Injury, poisoning and certain other consequences of external causes	19	3.5
Mental and behavioral disorders	56	10.4
Neoplasms	34	6.3
Pregnancy, childbirth, and the puerperium	9	1.7
Symptoms, signs, and abnormal clinical and laboratory findings, not elsewhere classified	25	4.6
Total	541	100.00

### 3.3 Gaps in WHO ICD‐10 medical classifications across 541 overviews

The WHO ICD‐10 medical classification has 22 classifications in total. No overviews were found for two WHO ICD‐10 medical classifications, namely ‘congenital malformations, deformations and chromosomal abnormalities’ and ‘diseases of the ear and mastoid process’ (Table 1).

### 3.4 Overlapping overviews according to medical classification

Of 541 overviews published from 2000 to 2018, 169 (31%) had at least one other overlapping overview (median 2 overviews per topic, IQR 2–3, maximum 6; listed in Appendix D). Of the 169 overlapping overviews, 39 (23%) were published on or before 2010, 61 (36%) from 2011 to 2015, and 70 (41%) from 2016 to 2019. These numbers show how incidence of overlapping overviews has changed chronologically.

The 169 overlapping overviews fell under 13/22 (59%) WHO ICD‐10 medical classifications. The greatest number of overlapping overviews were classified under “factors influencing health status and contact with health services” (40/169 [24%]), followed by “musculoskeletal system and connective tissue diseases” (35/169 [21%]), and “symptoms, signs and abnormal clinical and laboratory findings, not elsewhere classified” (23/169 [14%]).

### 3.5 Overlap in overview topics

A total of 62 topics involving a combination of the same population(s), intervention(s) and outcome(s) were covered by two or more of the 169 overlapping overviews (Table 2). One topic was covered by six overviews, namely behavioral counseling, and pharmacotherapy interventions for tobacco cessation. The topics of acupuncture for pain, cannabinoids for pain and symptoms, acupuncture for management of pregnancy‐related symptoms, and exercise therapy for bone and muscle health overlapped across five overviews each. Nine topics were covered by four overviews each, 13 topics were covered by three overviews each, and 34 topics were covered by two pairs of overviews (Table 2).

**TABLE 2:** Overview topics covered by 169 overlapping overviews (*n* = 62)

Overview topics	Frequency	Percentage by topic (%)
Behavioral counseling and pharmacotherapy interventions for tobacco cessation	6	9.7
Acupuncture for management of pregnancy‐related symptoms	5	8.1
Acupuncture for pain	5	8.1
Cannabinoids for pain, and symptoms	5	8.1
Exercise therapy	5	8.1
Exercise to relieve pain	4	6.5
Nonpharmacological treatment for behavioral and psychological disturbances	4	6.5
Over‐the‐counter analgesics for pain	4	6.5
Pharmacological, nonpharmacological, and surgical treatments of low back disorders	4	6.5
Reduction interventions of alcohol intake	4	6.5
Spinal manipulation	4	6.5
Surgical treatment of low back pain	4	6.5
Treatment of venous thromboembolism with LMWH and UFH	4	6.5
Vitamin D supplementation	4	6.5
Acupuncture for palliative care of cancer	3	4.8
Antipsychotic drugs for schizophrenia	3	4.8
Complementary and alternative procedures for fibromyalgia	3	4.8
Diets to reduce weight and obesity	3	4.8
Influenza vaccination	3	4.8
Interventions to treat complex wounds	3	4.8
Nonpharmacological interventions for osteoarthritis	3	4.8
Pharmacologic treatment of low back pain	3	4.8
Pharmacological and nonpharmacological treatments for depression	3	4.8
Physical activity promotion in children and adolescents	3	4.8
Preterm birth	3	4.8
Psychotherapy and nonmedication‐based interventions	3	4.8
Urinary incontinence	3	4.8
Acute asthma management in children	2	3.2
Assisted reproductive technologies (ARTs)	2	3.2
Childhood obesity interventions	2	3.2
Chronic treatment in childhood asthma	2	3.2
Cupping	2	3.2
Effects of coffee	2	3.2
Effects of financial arrangements for health systems in low‐income countries	2	3.2
Exercise to relieve fatigue	2	3.2
Food supplements for body weight reduction	2	3.2
Ginkgo biloba for dementia	2	3.2
Hip fracture pre‐op management and rehabilitation	2	3.2
Interventions for improving patient quality of life	2	3.2
Knee osteoarthritis ‐ physical therapy	2	3.2
Lung cancer cost effectiveness analysis	2	3.2
Lung cancer treatment	2	3.2
Lupus nephritis treatment	2	3.2
Mammography screening	2	3.2
Management of hip and knee osteoarthritis	2	3.2
Manual therapy for the treatment of migraine	2	3.2
Neuraminidase inhibitors for influenza	2	3.2
Nonpharmacological interventions for insomnia	2	3.2
Nonpharmacological treatment for cancer‐related fatigue	2	3.2
Opioid use in noncancer pain	2	3.2
Periodontal treatment and glycemic control	2	3.2
Pharmacological interventions for smoking cessation	2	3.2
Pharmacological treatments for major depressive disorder	2	3.2
Physiotherapy exercise	2	3.2
Physiotherapy and exercise	2	3.2
Rheumatoid arthritis ‐ nonparmacological interventions	2	3.2
Rheumatoid arthritis ‐ pharmacological interventions	2	3.2
Rotator cuff repair surgery rehabilitation	2	3.2
Therapy for fibromyalgia syndrome	2	3.2
Treatment for multiple myeloma	2	3.2
Web‐based interventions for weight loss	2	3.2

Appendix D lists the 169 overviews that overlapped across the 62 topics with their WHO ICD‐10 medical classification, population, interventions, and outcomes.

### 3.6 Classification of overlapping overviews as broad or narrow in scope

Our hypothesis that broad overviews were more prevalent than overviews with a narrow scope was supported, as 148/169 (88%) overlapping overviews were characterized as broad in scope (Appendix D). Most frequently, broad overviews had targeted populations for which multiple interventions were addressed (65/148 (44%]), and least frequently broad overviews addressed a targeted intervention for multiple populations [27/148 [18%]). Broad overviews categorized as nontargeted (56/148) accounted for 38% of broad overviews.

### 3.7 Classification of overlap as identical, nearly identical, partial, or subsumed

The 169 overviews overlapped such that a similar portion, major component(s), or complete representation of an overview was duplicated across one or more different overviews. The following characterizes the type of overlap found:

- 0 identical (0%),
- 15 nearly identical (9%),
- 31 subsumed (18%), and
- 123 partial (73%).

We did not identify any overviews for which overlap was identical to another overview according to our definition. The 15 overviews with nearly identical overlap spanned across seven topics (periodontal treatment and glycemic control [*n* = 2]; food supplements for body weight reduction [*n* = 2]; vitamin D supplementation [*n* = 3]; acupuncture for managing gynecologic conditions [*n* = 2]; acupuncture for management of pregnancy‐related symptoms [*n* = 2]; acupuncture for pain [*n* = 2]; rotator cuff repair surgery rehabilitation [*n* = 2]). A theme amongst nearly identical overviews was for slightly different outcomes to be investigated for the same patient population(s) and intervention(s). For example, overviews by Hasuike^28^ and Botero^29^ both addressed patients with Type 1 or Type 2 diabetes with periodontitis, receiving periodontal treatment, with a primary outcome of impact on hemoglobin A1c (HbA1c). Botero^29^ includes an additional secondary outcome of fasting blood glucose, which Hasuike^28^ does not. Hasuike^28^ was published after Botero^29^ but the dates of searches were comparable.

Two clusters of overviews (^30^, ^31^ and ^32^, ^33^) representing nearly identical overlap were from the same author group. In the latter example, the 2011 overview by Lee and Ernst^33^ included Cochrane reviews, whereas the 2011 overview by Ernst, Lee, and Choi^32^ included reviews of all types (Cochrane and non‐Cochrane).

A notable example of nearly identical overlap were three vitamin D overviews by Autier,^34^ Mateussi,^35^ and Rejnmark.^36^ They were categorized as nearly identical based on their similarly general populations and the intervention of vitamin D supplementation. Mateussi^35^ explored a broad range of outcomes which included skeletal outcomes. Interestingly, the aim of the two other overviews^27^, ^34^ was to examine specifically nonskeletal outcomes. Because there is overlap in at least one outcome (in fact, many are common to the three overviews) they are classified as nearly identical. However, the aims of the three are clearly different.

Subsummation of overviews could happen in different contexts. Some broad overviews covering an intervention for multiple patient populations subsumed one or multiple overviews investigating the intervention for just a portion of these populations. This situation is exemplified by the overlapping reviews by Posadzki,^37^, ^38^, ^39^ “Is spinal manipulation effective for pain? An overview of systematic reviews,” “Spinal manipulation: an update of a systematic review of systematic reviews,” and “Systematic reviews of spinal manipulations for headache: and attempt to clear up the confusion.” All three overviews address the targeted intervention of spinal manipulation for pain outcomes in varying populations. The former^37^ is a broad overview focusing on patients with any types of pain, which subsumes the more specific populations in the broad overview focusing on patients with pain from any type of clinical condition.^39^ Both of these overviews subsume the scope of the narrow overview investigating spinal manipulation in the specific sub‐population with headache.^38^

Sometimes overviews investigating multiple interventions in a targeted population subsumed others covering just a subset of these interventions. An example of this type of overlap happened with Chen's^40^ broad overview entitled “Treatment for lupus nephritis: an overview of systematic reviews and meta‐analyses,” and Mac‐Namara,^41^ “Is rituximab effective for induction of remission in lupus nephritis?” Both overviews investigated pharmacologic treatments in patients with lupus nephritis at various stages. Chen's^42^ overview broadly encompassed all interventions and more diverse regimens, which subsumed Mac‐Namara's^41^ overview which targeted any medication regimen involving rituximab specifically.

**FIGURE 3:**
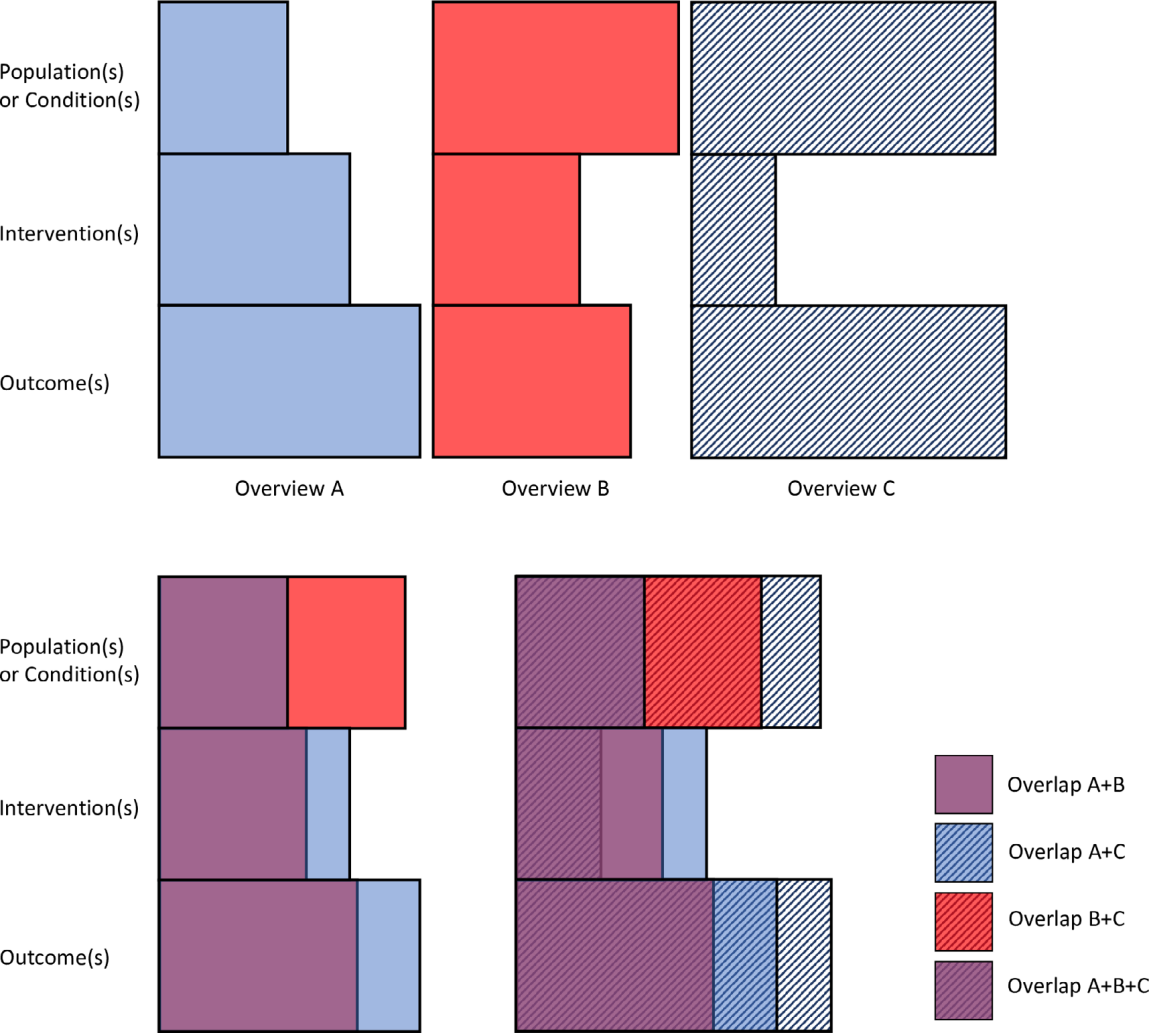
Example of three partially overlapping overviews. Figure 3 visually represents three broad, partially overlapping overviews from our sample [Colour figure can be viewed at wileyonlinelibrary.com]

Overviews were most often partially overlapping in various combinations of their populations, interventions, and outcomes. An example are the overviews by Wu, Towler, and Ezzo^33^, ^34^, ^35^ which are visually represented in Figure 3. Wu (Overview A in Figure 3)^35^ addressed any type of acupuncture therapy for cancer patients receiving palliative care. Towler (Overview B in Figure 3)^34^ addressed a narrower intervention, acupuncture excluding any acupressure therapy, for a slightly broader population of cancer patients receiving palliative or supportive care. Ezzo (Overview C in Figure 3)^33^ had an even narrower targeted intervention of acupuncture on point P6, and investigated multiple populations including cancer patients with chemotherapy‐induced nausea and vomiting, postoperative nausea and vomiting, and pregnancy‐related nausea and vomiting. Nausea and vomiting were outcomes common to all three overviews.

### 3.8 Authors who publish overviews on similar topics

Amongst groups of overlapping overviews, 28/169 (17%) had at least one author involved in each of the overviews that overlapped. Of these 28, seven were self‐reported as updates of older overviews (Appendix D). Subsummation was exemplified by three overviews published by a common author between 2011 and 2012 about the targeted intervention of spinal manipulation for pain.^43^, ^44^, ^45^ In another instance, one of the same authors published two studies on acupuncture for pain which represented nearly identical overlap.^32^, ^33^

### 3.9 Updated overviews or replications

Twelve studies (12/169 [7%]) were reported as updates of previous overviews (Appendix D), and we found no studies self‐reporting as a replication.

### 3.10 Overlap in Cochrane reviews

We identified 11 (7%) Cochrane overviews in our sample of 169 overlapping overviews. The majority (10/11 [91%]) were partially overlapping with non‐Cochrane overviews. One overview by Moore 2015^42^ was subsumed by a broader 2015 Cochrane overview by the same authors,^46^ and was partially overlapping with Els 2017.^47^

## 4 DISCUSSION

### 4.1 Summary of results

This is the first study to examine overlap across a sample of overviews. Our methodological assessment identified 31% of overviews dated between 2000 and 2018 that overlapped in content across 13 WHO ICD‐10 medical classifications and 62 topics. If we would have included overviews without systematic searches/methods sections, we believe there would have been more overlapping overviews identified, which points to why systematic methods are needed. As many as six overviews (median of 2) were completed on the same topic (e.g., behavioral counseling and pharmacotherapy interventions for tobacco cessation). We found that it was common for some overlapping overviews to cover broad topic areas, whereas others considered only subsets of the evidence. Though slight differences in the scope of the overlapping overviews were observed, we feel that at least some of the multiplicity represents unnecessary overlap, although we address legitimate reasons for overlap below. This study and the database of overviews found in Appendix D can provide a guide to authors about which topics are covered, and gaps in the evidence for future analysis. Overview authors can use the appendix to determine if the topic they are wanting to examine is already published. Our definitions of overlap can be used by other methodologists studying overlap across randomized controlled trials, systematic reviews, and overviews.

### 4.2 Legitimate reasons for observed overlap

Observed overlap can be legitimately justified for several reasons. Overviews may be out‐of‐date and therefore an update, including more recently published reviews, is needed. This reason is justified especially when systematic reviews on the topic are inconclusive, and their synthesis may reconcile discrepancies in their results, interpretation, and conclusions. For example, the overview by Doll^48^, published in 2017, addressing the safety and effectiveness of neuraminidase inhibitors for influenza treatment, prophylaxis, and outbreak control, provides and update of the literature reviewed in the overview published by Michiels in 2013,^49^ after which more systematic review evidence became available. A duplicate overview may be warranted when an older overview used inappropriate or invalid methods, or was of low methodological quality (e.g., if reassessments of risk of bias, re‐analyses of data, or [re]evaluation of GRADE assessments are required). Broader, more comprehensive overviews may be warranted if existing overviews are narrow in focus. A rationale for why an overlapping overview is needed should be provided by the authors and citing existing overviews known to the authorship team to make the case.

Finally, replication is also an appropriate reason to conduct an overview on the same topic with the same or similar PICO. Reproducibility of research by independent and conflict‐free academics to obtain the same (or similar) results when repeating an experiment or test is one of the hallmarks of good science.^50^ As defined by Karunananthan et al.^51^ for reviews, one type of replication involves repetition of PICO, using the same or very similar methods as a previous study to determine whether similar results can be obtained.^51^

With replication of the same research results, decision makers, healthcare workers and patients can be confident in the consistency and trustworthiness of the research.^37^ David Moher and colleagues have suggested two to three replicated systematic reviews with similar eligibility criteria would help ensure reliability of the findings, but four or more might represent unnecessary duplication and research waste.^37^ Postulating from the replication of systematic reviews literature, we hypothesized that the number of appropriate replications for overviews where there has been no change otherwise in the literature should be one to two. The overview replication(s) would have to be accompanied by a strong rationale such as re‐analyzing studies from included reviews when this was not done in the original overview.

### 4.3 Estimating the cost of research waste

Global research output is growing rapidly,^38^ as is the number of systematic reviews being produced^39^ which means that evidence syntheses now take longer and cost more to conduct than they did over 40 years ago.^52^ In 1999, it was estimated that meta‐analyses took on average of 1139 hours to complete,^53^ which has more than doubled in 2017 to approximately 2700 hours (i.e., 67.5 weeks).^54^ The cost of conducting a systematic review is considerable, recently estimated at $141,000 USD.^55^ For the Canadian context, the Canadian Institutes of Health Research would previously allocate $100,000 CAD for a knowledge synthesis grant for which a systematic review would be the intended output (e.g., [56]). Due to the challenges in searching for, collecting and synthesizing evidence in broad overviews, we estimate an overview could cost between $100,000 to $600,000 CAD. As a conservative estimate, of the 15 overviews that were nearly identical, two could be considered wasted. This would equate to a loss of between $200,000 and $1,200,000 Canadian research dollars.

### 4.4 Potential for discrepant or conflicting results and conclusions across overlapping overviews

We would hope that overviews with the same eligibility criteria would find the same results and come to similar conclusions, but this is often not the case with systematic reviews.^10^, ^11^, ^57^, ^58^, ^59^ For example, two nearly identical overviews^60^, ^61^ in our sample with range of motion (ROM), pain, functional improvements as outcomes and with 6/9 meta‐analyses in common, came to discordant conclusions. The overview by Mazuquin^61^ found that of seven meta‐analyses, early motion improves ROM after rotator cuff repair but increases the risk of rotator cuff retear. The overview by Houck,^60^ with nine included meta‐analyses, came to an opposing conclusion; namely that early motion does not improve ROM and does not cause a higher retear rate. Potential discrepancies like these can cause endless debates (e.g.,^62^), and confuse and possibly mislead clinicians and policymakers.^2^ Avoidance of this problem is the responsibility of researchers thinking about conducting overviews, funders, and publishers of this type of research.

### 4.5 Potential solutions and recommendations

To avoid unnecessary overlap and redundancy, several strategies can be used. First, protocols of overviews should be registered in a targeted database; second, journal editors and peer reviewers, funders and commissioners should require acknowledgement of other similar overviews and scrutinize the rationale where a de novo overview is proposed, and if one is found then suggest repurposing or updating the existing overview; and third, authors should cite the other known overviews that overlap in scope with a clinical or methodological rationale as to why the study is needed.^63^ Authors should clearly outline in their study protocol how their overview is different than earlier similar overviews based on: (a) the search strategy, (b) the results of the search, (c) the screening and inclusion criteria, (d) re‐analysis of review data, etc. and then justify why a new overview is needed. Authors should also define why the choice of an overview design is preferable over other synthesis types such as scoping reviews, systematic reviews with meta‐analyses, or network meta‐analyses.

We do not advocate that authors conduct ‘meta‐overviews’ (i.e., overviews of overviews) to try and explain differences in results across multiple overviews, as this would produce more redundant and potential useless research. Our list of 541 overviews have undergone some basic quality checks for inclusion (reported methods, conducted a systematic search, synthesized systematic review with or without meta‐analysis), and can be used in policy decisions. There is currently no quality appraisal tool for overviews, and hence overlapping overviews cannot at this time be chosen based on a quality assessment. To choose one overview amongst several on the same topic, we suggest policymakers choose the overview with the closest eligibility criteria in terms of population, interventions, and outcomes. In addition, we suggest at the very least using our stated inclusion criteria for the overviews examined in our report to ensure a minimum level of rigor. Recency and comprehensiveness of the evidence accumulated should be next examined to determine which overview should be chosen by healthcare providers and policymakers.

Currently, there is no dedicated database registry for protocols of overviews, such as there is for systematic reviews (i.e., PROSPERO). A dedicated database for overview protocols, as well as the development of a MeSH term for overviews, would help in their identification by prospective overview authors, who when finding a similar overview, could choose a different topic or new scope to explore. A published search filter for overviews^23^ can help in their identification, as would imbedded filters in databases such as Epistemonikos, MEDLINE, and Embase. The CDSR attempts to avoid duplication of effort by publishing only one overview and systematic review per topic of interest,^64^ although this has not been successfully empirically defended. In fact, in our study, we found subsummation and partial overlap across three Cochrane overviews. Other journals should follow suit and avoid duplicate publication of overviews with similar PICOs.

During the protocol phase and conduct of the overview, guidance^65^ and methodological studies^24^, ^66^, ^67^, ^68^ should be consulted to ensure rigor and a consistently high level of quality. Overviews of high quality will reduce the need for overlapping overviews and aid in avoiding wasting researchers' time, effort, and resources.

### 4.6 Principles of transparency and best practice in scholarly publishing

Finally, we are against the practice of duplicate publications of the same research in different journals. We speculate that this was done by the authors of two overviews on acupuncture for pain^32^, ^33^ as the 2011 overview was written by three authors, and a year later, two of these authors published an overview with the same populations, intervention, and efficacy outcomes, but this time focusing solely on Cochrane Reviews. The second paper references the earlier 2011 paper which was in print at the time, while providing a subsumed, subset of information from the earlier paper which we feel represents redundancy. Redundant publication is defined as findings that have previously been published elsewhere without proper attribution to previous sources or disclosure to the editor, permission to republish, or justification.^69^ These same Principles of Transparency and Best Practice in Scholarly Publishing^69^ state that “in cases of partial overlap (i.e., when authors present new findings in an article that contains a substantial amount of previously published information) editors should consider whether the entire article is retracted or whether to issue a correction.” Retractions may be used to alert readers to cases of *redundant publication*, or partial overlap.

### 4.7 Strengths and limitations

Despite the growing popularity of overviews as a method to synthesize systematic reviews, to our knowledge this is the first study to examine overlap across a sample of overviews. A strength of our research is that we based our methods on systematic review guidance, and searched using a validated search strategy for overviews. We selected the overviews based on stringent eligibility criteria using two independent reviewers, who then compared their results and identified and resolved discrepancies. We acknowledge, however, that our methods were not outlined in a protocol and registered prior to undertaking this meta‐research study. We recognize that this reduces the transparency of our preplanned analyses and created the potential for redundancy in the efforts of this and other similar studies. We did, however, acknowledge transparently our post hoc analyses and declare that our categorization of overlap is therefore exploratory. A second study that uses our classification system and methods to preplan the analysis could be used to validate our findings.

A limitation to our study was the subjective nature of classifying overlapping topics in overviews. Many broad overview topics could have been classified under several ICD‐10 classifications. For example, the overview entitled “Melatonin for health” was classified under “Mental and behavioral disorders” due to the primary theme of its outcomes (sleep latency, pre‐operative anxiety, prevention of agitation and risk of breast cancer). This same overview could have also been classified under the ICD‐10 classification “Factors influencing health status and contact with health services”. There was also room for error or subjectivity when extracting the overview eligibility criteria, that is, PICOs to identify overlap, and classifying the type of overlap represented across overlapping overviews. Performing these steps in duplicate was our attempt to ensure accuracy but we recognize that some of the overview topics were specialized and nuanced. Another limitation is that we excluded protocols of overviews; including them would have given us a broader sense of overlap.

## 5 CONCLUSIONS

Our empirical evaluation identified overlap in 31% of overviews dated between 2000 and 2018. Sixty‐two topics across 13 WHO ICD‐10 medical classifications had overlapping overviews. As many as six overviews addressed the same topic. We found that it was common for overlapping overviews to cover broad topic areas. Most frequently, broad overviews had targeted populations for which multiple interventions were addressed, and least frequently broad overviews addressed a targeted intervention for multiple populations. Our taxonomy of identical, nearly identical, partial, and subsumed overlap can be used by other methodologists studying overlap across randomized controlled trials, systematic reviews, and overviews. A multiplicity of overviews on the same topic adds to the ongoing waste of research resources, time, and effort across medical disciplines.

To avoid duplication of research and redundancy, protocols of overviews should be registered in an open registry like the Open Science Framework or as a preprint, and overviews should cite others on similar PICO topics with a rationale as to why they are undertaking the overview despite existence of others. Authors of overviews can use this study and the sample of overviews to identify topics and PICO eligibility criteria that are already covered, and gaps in the evidence for future analysis.

## CONFLICT OF INTEREST

The authors have declared no competing interest.

## AUTHORS' CONTRIBUTIONS

Carole Lunny conceived of the study design, wrote the study protocol, analyzed the data, wrote, and revised the final manuscript. Gavindeep Shinger, Yi Wen Zheng, Carole Lunny, Lester Lin, Pei Hsuan Yang, Shadi Sadeghipouya, Manpreet Dosanjh, Peter Ngsee screened citations and full‐text articles. Trish Neelakant, Adrienne Stevens, Gavindeep Shinger, Sara Tasnim, Stephen Adams, Ursula Ellis, Jia He Zhang, Emma K. Reid abstracted and verified data. Carole Lunny, Emma K. Reid, Trish Neelakant, Beverley J. Shea, and Adrienne Stevens analyzed the data. Carole Lunny, Emma K. Reid, and Beverley J. Shea interpreted results, and edited the manuscript. Trish Neelakant, AC, Gavindeep Shinger, Adrienne Stevens, Sara Tasnim, Shadi Sadeghipouya, Stephen Adams, Yi Wen Zheng, Lester Lin, Pei Hsuan Yang, Manpreet Dosanjh, Peter Ngsee, Ursula Ellis, Beverley J. Shea, Emma K. Reid, and James M. Wright edited and approved of the final manuscript.

## Supporting information


**Appendix S1**: Supporting Information

**Appendix S2**: Supporting Information

## Data Availability

Data sharing and availability of data and materials. The full dataset in Appendix D is available on https://osf.io/q9t63
