# Supplementary material for: A new taxonomy was developed for overlap across 'overviews of systematic reviews': A meta‐research study of research waste
Source: Res Synth Methods. 2022 Jan 23;13(3):315–29. doi: 10.1002/jrsm.1542 (PMC9303867; doi:10.1002/jrsm.1542)
Supplement: Supplementary file 1 — Appendix S1: Supporting Information [file JRSM-13-315-s001.docx]

**Appendices**

**Appendix A – Search Strategy**

**Appendix B - Figure 1: PRISMA flowchart of study selection**

**Appendix C – 541 overviews included**

**Appendix A – Search Strategy**

**Ovid MEDLINE (**Ovid MEDLINE(R) In-Process & Other Non-Indexed Citations, Ovid MEDLINE(R) Daily, Ovid MEDLINE(R) and Ovid OLDMEDLINE(R) 1946 to Present)

Dates searched: Jan 2000 to Dec 2012; and Jan 2017 to Jan 8, 2021

*Sensitivity-maximising strategy (Lunny 2016) :*

((overview$ or review or synthesis or summary or Cochrane or analysis) and (reviews or meta-analyses or articles or umbrella)).ti. or "umbrella review".ab. or (meta-review or metareview).ti,ab. or ((overview$ or reviews) and (systematic or Cochrane)).ti. or (reviews adj2 (meta or published or quality or included or summar$)).ab. or "cochrane reviews".ab. or (evidence and (reviews or meta-analyses)).ti.

**Epistemonikos**

Dates searched: inception to Jan 8, 2021

Limit: Broad syntheses

(title:(overview OR review of reviews OR meta-review OR umbrella review OR review of systematic) OR abstract:(overview OR review of reviews OR meta-review OR umbrella review OR review of systematic))

**Cochrane Database of Systematic Reviews of Interventions**

Dates searched: Jan 2000 to Jan 8, 2021

Limit: Overviews of systematic reviews

**Other sources**

Lunny 2016 (n = 187):

Sample was comprised of the overviews retrieved from screening the combined results from the search strategies of Hartling et al., Pieper et al. (2012 and 2014), and an Ovid MEDLINE search over the publication years 2012 to 2014 and limited to English language and humans.

Lunny 2020 (n = 50):

MEDLINE was searched by using a validated search strategy for overviews (Lunny 2015) restricting the search from January 2015 to March 2017.

**References:**

Lunny C, McKenzie JE, McDonald S. Retrieval of overviews of systematic reviews in MEDLINE was improved by the development of an objectively derived and validated search strategy. Journal of clinical epidemiology. 2016 Jun 1;74:107-18.

Lunny C, Brennan SE, Reid J, McDonald S, McKenzie JE. Overviews of reviews incompletely report methods for handling overlapping, discordant, and problematic data. Journal of clinical epidemiology. 2020 Feb 1;118:69-85.

**Appendix B - Figure 1: PRISMA flowchart of study selection**


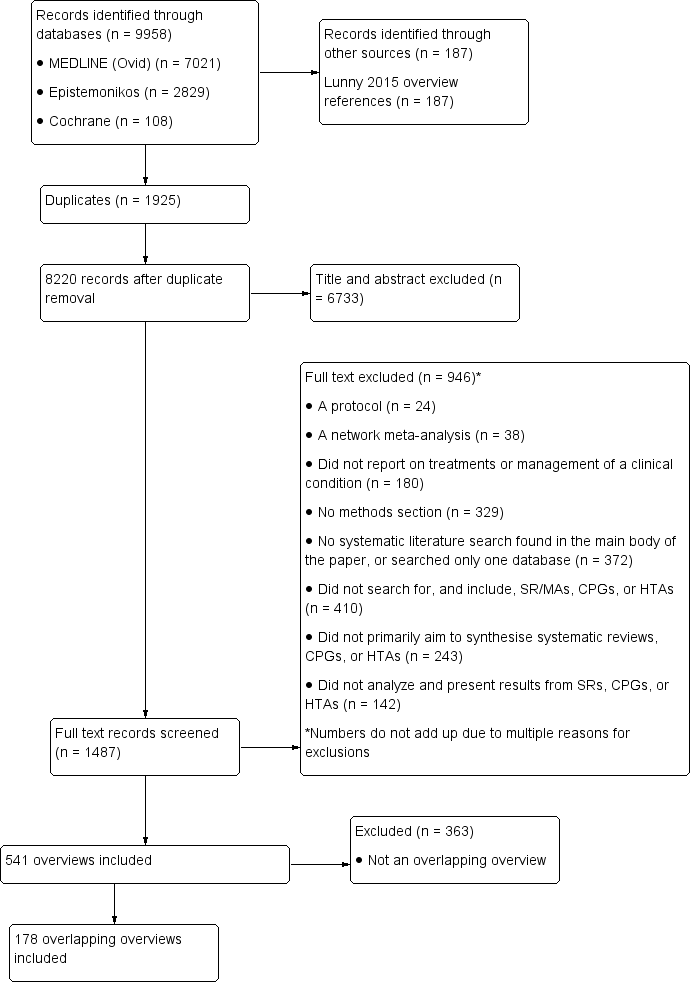


**Appendix C – 541 overviews included**

| Aberdein J, Singer M. Clinical review: a systematic review of corticosteroid use in infections. Critical Care. 2005 Feb;10(1):203. https://www.ncbi.nlm.nih.gov/pmc/articles/PMC1550829/ |
| --- |
| Abraha I, Rimland JM, Trotta FM, Dell'Aquila G, Cruz-Jentoft A, Petrovic M, Gudmundsson A, Soiza R, O'Mahony D, Guaita A, Cherubini A. Systematic review of systematic reviews of non-pharmacological interventions to treat behavioural disturbances in older patients with dementia. The SENATOR-OnTop series. BMJ open. 2017 Mar 1;7(3):e012759. https://www.ncbi.nlm.nih.gov/pmc/articles/PMC5372076/ |
| Abraha I, Trotta F, Rimland JM, Cruz-Jentoft A, Lozano-Montoya I, Soiza RL, Pierini V, Fulgheri PD, Lattanzio F, O’Mahony D, Cherubini A. Efficacy of non-pharmacological interventions to prevent and treat delirium in older patients: a systematic overview. The SENATOR project ONTOP Series. PloS one. 2015 Jun 10;10(6):e0123090. https://www.ncbi.nlm.nih.gov/pubmed/26062023 |
| Abramowitz J, Thakkar P, Isa A, Truong A, Park C, Rosenfeld RM. Adverse event reporting for proton pump inhibitor therapy: an overview of systematic reviews. Otolaryngology–Head and Neck Surgery. 2016 Oct;155(4):547-54. https://www.ncbi.nlm.nih.gov/pubmed/27188706 |
| Abu-Omar K, Ruetten A, Burlacu I, Messing S, Pfeifer K, Ungerer-Röhrich U. A systematic review of reviews of interventions for the promotion of physical activity: methodology and first results. https://www.ncbi.nlm.nih.gov/pubmed/28399586 |
| Adam SS, McDuffie JR, Lachiewicz PF, Ortel TL, Williams JW. Comparative effectiveness of new oral anticoagulants and standard thromboprophylaxis in patients having total hip or knee replacement: a systematic review. Annals of internal medicine. 2013 Aug 20;159(4):275-84. https://www.ncbi.nlm.nih.gov/pubmed/?term=Ann+Intern+Med.+2013%3B159%3A275-284. |
| Adams LV, Talbot EA, Odato K, Blunt H, Steingart KR. Interventions to improve delivery of isoniazid preventive therapy: an overview of systematic reviews. BMC infectious diseases. 2014 Dec;14(1):281. https://www.ncbi.nlm.nih.gov/pmc/articles/PMC4038070/ |
| Adams NP, Jones PW. The dose–response characteristics of inhaled corticosteroids when used to treat asthma: an overview of Cochrane systematic reviews. Respiratory medicine. 2006 Aug 1;100(8):1297-306. https://www.ncbi.nlm.nih.gov/pubmed/?term=Respiratory+Medicine+(2006)+100%2C+1297–1306 |
| Aguiar PM, de Mendonça Lima T, Colleoni GW, Storpirtis S. Efficacy and safety of bortezomib, thalidomide, and lenalidomide in multiple myeloma: an overview of systematic reviews with meta-analyses. Critical reviews in oncology/hematology. 2017 May 1;113:195-212. https://www.ncbi.nlm.nih.gov/pubmed/28427509 |
| Ah-See K. Sinusitis (acute). BMJ clinical evidence. 2011;2011. https://www.ncbi.nlm.nih.gov/pmc/articles/PMC3275132/ |
| Ahumada V, Moraga F, Rada G. Laparoscopy or open surgery for the treatment of hydatid cyst?. Medwave. 2016 Mar 22;16(Suppl 1):e6385. https://www.medwave.cl/link.cgi/English/Updates/Epistemonikos/6386?ver=sindiseno |
| Akl EA, Muti P, Schunemann HJ. Anticoagulation in patients with cancer: an overview of reviews. Polskie Archiwum Medycyny Wewnetrznej. 2008 Apr;118(4):183. https://www.ncbi.nlm.nih.gov/pubmed/18575417 |
| Alderdice F, McNeill J, Lynn F. A systematic review of systematic reviews of interventions to improve maternal mental health and well-being. Midwifery. 2013 Apr 1;29(4):389-99. https://www.ncbi.nlm.nih.gov/pubmed/22882967 |
| Al-Hameed F, Al-Dorzi HM, Al Momen A, Algahtani F, Al Zahrani H, Al Saleh K, Al Sheef M, Owaidah T, Alhazzani W, Neumann I, Wiercioch W. Prophylaxis and treatment of venous thromboembolism in patients with cancer: the Saudi clinical practice guideline. Annals of Saudi medicine. 2015 Mar;35(2):95-106. https://www.ncbi.nlm.nih.gov/pubmed/26336014 |
| Allan GM, Finley CR, Ton J, Perry D, Ramji J, Crawford K, Lindblad AJ, Korownyk C, Kolber MR. Systematic review of systematic reviews for medical cannabinoids: Pain, nausea and vomiting, spasticity, and harms. Canadian Family Physician. 2018 Feb 1;64(2):e78-94. https://www.cfp.ca/content/cfp/64/2/e78.full.pdf |
| Alvarez-Bueno C, Rodriguez-Martin B, Garcia-Ortiz L, Gómez-Marcos MÁ, Martinez-Vizcaino V. Effectiveness of brief interventions in primary health care settings to decrease alcohol consumption by adult non-dependent drinkers: a systematic review of systematic reviews. Preventive Medicine. 2015 Jul 1;76:S33-8. https://www.ncbi.nlm.nih.gov/pubmed/?term=25514547 |
| Amato L, Davoli M, Ferri M, Gowing L, Perucci CA. Effectiveness of interventions on opiate withdrawal treatment: an overview of systematic reviews. Drug and alcohol dependence. 2004 Mar 8;73(3):219-26. https://www.ncbi.nlm.nih.gov/pubmed/15036544 |
| Amato L, Minozzi S, Davoli M. Efficacy and safety of pharmacological interventions for the treatment of the Alcohol Withdrawal Syndrome. Cochrane database of systematic reviews. 2011(6). https://www.ncbi.nlm.nih.gov/pubmed/21678378 |
| Anderson IM. Meta-analytical studies on new antidepressants. British Medical Bulletin. 2001 Mar 1;57(1):161-78. https://doi.org/10.1093/bmb/57.1.161 |
| Anderson L, Taylor RS. Cardiac rehabilitation for people with heart disease: an overview of Cochrane systematic reviews. Cochrane Database of Systematic Reviews. 2014(12). https://www.ncbi.nlm.nih.gov/pubmed/25503364 |
| Aneiba KM, Tashani OA. Artificial Disc Replacement versus Fusion in the Cervical Spine: A Systematic Review of Systematic Reviews. Global Spine Journal. 2014 May;4(1_suppl):s-0034. http://sci-hub.tw/10.1055/s-0034-1376700 |
| Ashfaq K, Yahaya I, Hyde C, et al. Clinical effectiveness and cost-effectiveness of stem cell transplantation in the management of acute leukaemia: a systematic review. Health Technol Assess. 2010;14(54):iii-141. https://www.ncbi.nlm.nih.gov/pubmed/21138675 |
| Atkinson RA, Cullum NA. Interventions for pressure ulcers: a summary of evidence for prevention and treatment. Spinal Cord. 2018 Mar;56(3):186. https://www.ncbi.nlm.nih.gov/pubmed/29371701 |
| Autier P, Mullie P, Macacu A, Dragomir M, Boniol M, Coppens K, Pizot C, Boniol M. Effect of vitamin D supplementation on non-skeletal disorders: a systematic review of meta-analyses and randomised trials. The lancet Diabetes & endocrinology. 2017 Dec 1;5(12):986-1004. https://www.ncbi.nlm.nih.gov/pubmed/29102433 |
| Azar FE, Azami-Aghdash S, Pournaghi-Azar F, Mazdaki A, Rezapour A, Ebrahimi P, Yousefzadeh N. Cost-effectiveness of lung cancer screening and treatment methods: a systematic review of systematic reviews. BMC health services research. 2017 Dec;17(1):413. https://www.ncbi.nlm.nih.gov/pmc/articles/PMC5477275/pdf/12913_2017_Article_2374.pdf |
| Babatunde OO, Jordan JL, Van der Windt DA, Hill JC, Foster NE, Protheroe J. Effective treatment options for musculoskeletal pain in primary care: A systematic overview of current evidence. PloS one. 2017 Jun 22;12(6):e0178621. https://www.ncbi.nlm.nih.gov/pmc/articles/PMC5480856/ |
| Bachmann M, Bachmann C, Rief W, Mattejat F. Efficacy of psychiatric and psychotherapeutic interventions in children and adolescents with psychiatric disorders--a systematic evaluation of meta-analyses and reviews. Part II: ADHD and conduct disorders. Zeitschrift fur Kinder-und Jugendpsychiatrie und Psychotherapie. 2008 Sep;36(5):321. https://www.ncbi.nlm.nih.gov/pubmed/18791982 |
| Bailey C. Stomach cancer. BMJ clinical evidence. 2011;2011. https://www.ncbi.nlm.nih.gov/pmc/articles/PMC3275308/ |
| Bała MM, Leśniak W, Strzeszyński Ł. Efficacy of pharmacological methods used for treating tobacco dependence: meta-analysis. Polskie Archiwum Medycyny Wewnetrznej. 2008;118(1-2):20-8. https://www.researchgate.net/profile/Wiktoria_Lesniak/publication/5447853_Efficacy_of_pharmacological_methods_used_for_treating_tobacco_dependence_meta-analysis/links/565785f308aeafc2aac109c1.pdf |
| Bala MM, Lesniak W. Efficacy of non-pharmacological methods used for treating tobacco dependence: meta-analysis. Polskie Archiwum Medycyny Wewnetrznej. 2007;117(11/12):504. https://www.researchgate.net/profile/Malgorzata_Bala2/publication/5489648_Efficacy_of_non-pharmacological_methods_used_for_treating_tobacco_dependence_Meta-analysis/links/0c96051b63350608e2000000/Efficacy-of-non-pharmacological-methods-used-for-treating-tobacco-dependence-Meta-analysis.pdf |
| Bao Y, Kong X, Yang L, Liu R, Shi Z, Li W, Hua B, Hou W. Complementary and alternative medicine for cancer pain: an overview of systematic reviews. Evidence-Based Complementary and Alternative Medicine. 2014;2014. https://www.ncbi.nlm.nih.gov/pubmed/24817897 |
| Bardus M, Smith JR, Samaha L, Abraham C. Mobile and Web 2.0 interventions for weight management: an overview of review evidence and its methodological quality. The European Journal of Public Health. 2016 Jun 21;26(4):602-10. https://www.ncbi.nlm.nih.gov/pubmed/27335330 |
| Barlow J, McMillan AS, Kirkpatrick S, Ghate D, Barnes J, Smith M. Health‐led interventions in the early years to enhance infant and maternal mental health: A review of reviews. Child and Adolescent Mental Health. 2010 Nov;15(4):178-85. https://onlinelibrary.wiley.com/doi/abs/10.1111/j.1475-3588.2010.00570.x |
| Barnes NP. Migraine headache in children. BMJ clinical evidence. 2015;2015. https://www.ncbi.nlm.nih.gov/pmc/articles/PMC4456852/pdf/2015-0318.pdf |
| Bashi N, Karunanithi M, Fatehi F, Ding H, Walters D. Remote monitoring of patients with heart failure: an overview of systematic reviews. Journal of medical Internet research. 2017;19(1):e18. https://www.ncbi.nlm.nih.gov/pubmed/28108430 |
| Befus D, Coeytaux RR, Goldstein KM, McDuffie JR, Shepherd-Banigan M, Goode AP, Kosinski A, Van Noord MG, Adam SS, Masilamani V, Nagi A. Management of menopause symptoms with acupuncture: an umbrella systematic review and meta-analysis. The Journal of Alternative and Complementary Medicine. 2018 Apr 1;24(4):314-23. https://www.ncbi.nlm.nih.gov/pubmed/29298078 |
| Bellamy RJ. HIV: treating Pneumocystis pneumonia (PCP). BMJ clinical evidence. 2008;2008. https://www.ncbi.nlm.nih.gov/pmc/articles/PMC2907978/pdf/2008-2501.pdf |
| Bennett K, Manassis K, Duda S, Bagnell A, Bernstein GA, Garland EJ, Miller LD, Newton A, Thabane L, Wilansky P. Treating child and adolescent anxiety effectively: Overview of systematic reviews. Clinical Psychology Review. 2016 Dec 1;50:80-94. https://www.ncbi.nlm.nih.gov/pubmed/27744168 |
| Bergamo TR, Latorraca COC, Pachito DV, Martimbianco ALC, Riera R. Findings and methodological quality of systematic reviews focusing on acupuncture for pregnancy-related acute conditions. Acupuncture in medicine : journal of the British Medical Acupuncture Society. 2018;36(3):146-52. |
| Biddle SJ, Asare M. Physical activity and mental health in children and adolescents: a review of reviews. British journal of sports medicine. 2011 Sep 1;45(11):886-95. https://www.ncbi.nlm.nih.gov/pubmed/21807669 |
| Biddle SJ, Petrolini I, Pearson N. Interventions designed to reduce sedentary behaviours in young people: a review of reviews. Br J Sports Med. 2014 Feb 1;48(3):182-6. https://www.ncbi.nlm.nih.gov/pubmed/24347578 |
| Bidonde J, Jean Busch A, Bath B, Milosavljevic S. Exercise for adults with fibromyalgia: an umbrella systematic review with synthesis of best evidence. Current rheumatology reviews. 2014 Apr 1;10(1):45-79. https://www.ncbi.nlm.nih.gov/pubmed/25229499 |
| Bloom BS. Effects of continuing medical education on improving physician clinical care and patient health: a review of systematic reviews. International journal of technology assessment in health care. 2005 Jul;21(3):380-5. https://www.ncbi.nlm.nih.gov/pubmed/16110718 |
| Bobrovitz N, Heneghan C, Onakpoya I, Fletcher B, Collins D, Tompson A, Lee J, Nunan D, Fisher R, Scott B, O’Sullivan J. Medications that reduce emergency hospital admissions: an overview of systematic reviews and prioritisation of treatments. BMC medicine. 2018 Dec;16(1):115. https://www.ncbi.nlm.nih.gov/pubmed/30045724 |
| Boluyt N, Van der Lee JH, Moyer VA, Brand PL, Offringa M. State of the evidence on acute asthma management in children: a critical appraisal of systematic reviews. Pediatrics. 2007 Dec 1;120(6):1334-43. https://www.ncbi.nlm.nih.gov/pubmed/18055684 |
| Boric K, Dosenovic S, Jelicic Kadic A, Batinic M, Cavar M, Urlic M, Markovina N, Puljak L. Interventions for postoperative pain in children: An overview of systematic reviews. Pediatric Anesthesia. 2017 Sep;27(9):893-904. https://www.ncbi.nlm.nih.gov/pubmed/28707454 |
| Botero JE, Rodriguez C, Agudelo‐Suarez AA. Periodontal treatment and glycaemic control in patients with diabetes and periodontitis: an umbrella review. Australian dental journal. 2016 Jun;61(2):134-48. https://www.ncbi.nlm.nih.gov/pubmed/26815303 |
| Bouzid M, Brainard J, Hooper L, Hunter PR. Public health interventions for Aedes control in the time of Zikavirus–a meta-review on effectiveness of vector control strategies. PLoS neglected tropical diseases. 2016 Dec 7;10(12):e0005176. https://www.ncbi.nlm.nih.gov/pmc/articles/PMC5142773/ |
| Brackstone M, Fletcher GG, Dayes IS, Madarnas Y, SenGupta SK, Verma S. Locoregional therapy of locally advanced breast cancer: a clinical practice guideline. Current Oncology. 2015 Mar;22(Suppl 1):S54. https://www.ncbi.nlm.nih.gov/pmc/articles/PMC4381791/pdf/conc-22-s54.pdf |
| Bradbury P, Sivajohanathan D, Chan A, Kulkarni S, Ung Y, Ellis PM. Postoperative Adjuvant Systemic Therapy in Completely Resected Non–Small-Cell Lung Cancer: A Systematic Review. Clinical lung cancer. 2017 May 1;18(3):259-73. https://www.ncbi.nlm.nih.gov/pubmed/28162945 |
| Bradley JM, Moran FM, Elborn JS. Evidence for physical therapies (airway clearance and physical training) in cystic fibrosis: an overview of five Cochrane systematic reviews. Respiratory medicine. 2006 Feb 1;100(2):191-201. https://www.ncbi.nlm.nih.gov/pubmed/16412951 |
| Brown J, Farquhar C. Endometriosis: an overview of Cochrane Reviews. Cochrane Database of Systematic Reviews. 2014(3). https://www.ncbi.nlm.nih.gov/pubmed/24610050 |
| Browne G, Gafni A, Roberts J, Byrne C, Majumdar B. Effective/efficient mental health programs for school-age children: a synthesis of reviews. Social science & medicine. 2004 Apr 1;58(7):1367-84. https://doi.org/10.1016/S0277-9536(03)00332-0 |
| Brunström M, Eliasson M, Nilsson PM, Carlberg B. Blood pressure treatment levels and choice of antihypertensive agent in people with diabetes mellitus: an overview of systematic reviews. Journal of hypertension. 2017 Mar 1;35(3):453-62. https://www.ncbi.nlm.nih.gov/pubmed/27870655 |
| Buchberger B, Krabbe L. Evaluation of outpatient acupuncture for relief of pregnancy‐related conditions. International Journal of Gynecology & Obstetrics. 2018 May;141(2):151-8. https://www.ncbi.nlm.nih.gov/pubmed/29355951 |
| Cahill K, Stevens S, Perera R, Lancaster T. Pharmacological interventions for smoking cessation: an overview and network meta‐analysis. Cochrane database of systematic reviews. 2013(5). |
| Campbell JM, Bateman E, Peters MD, Bowen JM, Keefe DM, Stephenson MD. Fluoropyrimidine and platinum toxicity pharmacogenetics: an umbrella review of systematic reviews and meta-analyses. Pharmacogenomics. 2016 Mar;17(4):435-51. https://www.ncbi.nlm.nih.gov/pubmed/26894782 |
| Campbell JM, Bateman E, Stephenson MD, Bowen JM, Keefe DM, Peters MD. Methotrexate-induced toxicity pharmacogenetics: an umbrella review of systematic reviews and meta-analyses. Cancer chemotherapy and pharmacology. 2016 Jul 1;78(1):27-39. https://www.ncbi.nlm.nih.gov/pubmed/27142726 |
| Campbell JM, Stephenson MD, Bateman E, Peters MD, Keefe DM, Bowen JM. Irinotecan-induced toxicity pharmacogenetics: an umbrella review of systematic reviews and meta-analyses. The pharmacogenomics journal. 2017 Jan;17(1):21. https://www.ncbi.nlm.nih.gov/pubmed/27503581 |
| Canadian Agency for Drugs and Technologies in Health. "Cannabinoid Buccal Spray for Chronic Non-Cancer or Neuropathic Pain: A Review of Clinical Effectiveness, Safety, and Guidelines." *CADTH Rapid Response Reports* (2016). |
| Canadian Agency for Drugs and Technologies in Health. Treatments for constipation: A review of systematic reviews. CADTH Rapid Response Reports, Ottawa, Canada. 2014. https://www.ncbi.nlm.nih.gov/books/NBK263445/ |
| Canadian Agency for Drugs and Technologies in Health. Use of Surgical Masks in the Operating Room: A Review of the Clinical Effectiveness and Guidelines. Ottawa (ON): Canadian Agency for Drugs and Technologies in Health; 2013. https://www.ncbi.nlm.nih.gov/books/NBK195773/ Nov. CADTH Rapid Response Reports. https://www.ncbi.nlm.nih.gov/books/NBK195776/ |
| Cao H, Han M, Zhu X, Liu J. An overview of systematic reviews of clinical evidence for cupping therapy. Journal of Traditional Chinese Medical Sciences. 2015 Jan 1;2(1):3-10. https://www.sciencedirect.com/science/article/pii/S2095754815000277?via%3Dihub |
| Castro-Gutiérrez V, Rada G. Is fluid restriction needed in heart failure?. Medwave. 2017 Jan 9;17(Suppl1): e6817. https://doi.org/10.5867/medwave.2017.6817 |
| Castro-Gutierrez V, Rada G. Is there a role for glutamine supplementation in the management of acute pancreatitis?. Medwave. 2016 Aug 17;16(Suppl3):6512. https://www.medwave.cl/link.cgi/English/Updates/Epistemonikos/6513?ver=sindiseno |
| Castro-Rodriguez JA, Rodrigo GJ, Rodriguez-Martinez CE. Principal findings of systematic reviews for chronic treatment in childhood asthma. Journal of Asthma. 2015 Apr 21;52(4):407-16. https://www.ncbi.nlm.nih.gov/pubmed/25275887 |
| Catalan-Matamoros D, Gomez-Conesa A, Stubbs B, Vancampfort D. Exercise improves depressive symptoms in older adults: an umbrella review of systematic reviews and meta-analyses. Psychiatry Research. 2016 Oct 30;244:202-9. https://www.ncbi.nlm.nih.gov/pubmed/27494042 |
| Cates CJ, Oleszczuk M, Stovold E, Wieland LS. Safety of regular formoterol or salmeterol in children with asthma: an overview of Cochrane reviews. Cochrane Database of Systematic Reviews. 2012(10). https://www.ncbi.nlm.nih.gov/pmc/articles/PMC4022036/ |
| Cates CJ, Wieland LS, Oleszczuk M, Kew KM. Safety of regular formoterol or salmeterol in adults with asthma: an overview of Cochrane reviews. Cochrane Database of Systematic Reviews. 2014(2). |
| Chan WV, Pearson TA, Bennett GC, Cushman WC, Gaziano TA, Gorman PN, Handler J, Krumholz HM, Kushner RF, MacKenzie TD, Sacco RL. ACC/AHA special report: clinical practice guideline implementation strategies: a summary of systematic reviews by the NHLBI Implementation Science Work Group: a report of the American College of Cardiology/American Heart Association Task Force on Clinical Practice Guidelines. Journal of the American College of Cardiology. 2017 Feb 20;69(8):1076-92. https://www.ncbi.nlm.nih.gov/pubmed/28132746 |
| Chen Y, Sun J, Yang Y, Huang Y, Liu G. Malignancy risk of anti-tumor necrosis factor alpha blockers: an overview of systematic reviews and meta-analyses. Clinical rheumatology. 2016 Jan 1;35(1):1-8. https://link.springer.com/article/10.1007/s10067-015-3115-7 |
| Chen Y, Sun J, Zou K, Yang Y, Liu G. Treatment for lupus nephritis: an overview of systematic reviews and meta-analyses. Rheumatology international. 2017 Jul 1;37(7):1089-99. https://www.ncbi.nlm.nih.gov/pubmed/28493175 |
| Cheng H, Clymer JW, Sadeghirad B, Ferko NC, Cameron CG, Amaral JF. Performance of Harmonic devices in surgical oncology: an umbrella review of the evidence. World journal of surgical oncology. 2018 Dec;16(1):2. https://www.ncbi.nlm.nih.gov/pmc/articles/PMC5755263/ |
| **Cheung A, Weir M, Mayhew A, Kozloff N, Brown K, Grimshaw J. Overview of systematic reviews of the effectiveness of reminders in improving healthcare professional behavior. Syst Rev. 2012 Aug 16;1:36.** |
| Childs SM, Roberts AP, Meineche-Schmidt V, De Wit NJ, Rubin GP. The management of Helicobacter pylori infection in primary care: a systematic review of the literature. Family practice. 2000 Aug 1;17(suppl_2):S6-s11. https://watermark.silverchair.com/170S06.pdf?token=AQECAHi208BE49Ooan9kkhW_Ercy7Dm3ZL_9Cf3qfKAc485ysgAAAnEwggJtBgkqhkiG9w0BBwagggJeMIICWgIBADCCAlMGCSqGSIb3DQEHATAeBglghkgBZQMEAS4wEQQMlceUBsZj68UeRVqhAgEQgIICJIWUpL2RaG6dCG5cGPefHEt0YUnyFxL7KO7jURnsn7oRLowb2q9JYZKCy3Z3d2AfMtcRcknZa1AuA8IXsRu0YCv6Gursfx01pb4SSSiokMYEjKcwJtcwUlALGlNhltWa-w4jIxWeU3tpSB01XQW3qfmnXwuMj-9Te4qexjurUOBNGGiDAJq1eCQTuoNckDTVDglagW6ZkddQr66qgFtDsKt-LogFCYDYge3IrpmNjFeYIrDWJTBv4E6XurHdFrYVPb4Y4TquXYuGwcwc9E5hVqJ-05d15I51qRTwpFz0bMLfiZ63d79rkC12WUpYotzcbLtEpQdsfU-MqpkoEVlWCmS5KajjhS5bi9wyLT1TnyRmFh9yDjOv6kIgcpb08WZLtXLNPJhOcKIXKDCGhg4v0txHR0yHHC1AeyafGyOVISfyefVYopjFZn-9heKuhE2eo3ZedaQ72tEAHTE6KoINYvOLh366ewYwp88u22vO2BiF4cAmf-60H4NNBZWN_pNfbLYbl11a8QtO0SpsdZ2G6GaSPSMGwW7A0jSYNzQJMAPRI19nGw6sS3N4uOWK05s0dKfJGkIVsj6kRfUpRTNlQKQv1V9Sj6AtiDRsF7gNXM1jH1HfrzIMX6xtt__d_eMs_yYrPIHSVM_VRBV3Um_lIB8HTu3vw3HcqCenhf9zdm9dJ-acq7E7NTooSx3-0wGsk8jy6qHcAEUUmQUWSZrJS2wxceAA |
| Chou R, Huffman LH. Medications for acute and chronic low back pain: a review of the evidence for an American Pain Society/American College of Physicians clinical practice guideline. Annals of internal medicine. 2007 Oct 2;147(7):505-14. https://www.ncbi.nlm.nih.gov/pubmed/17909211 |
| Chou R, McDonagh MS, Nakamoto E, Griffin J. Analgesics for osteoarthritis: an update of the 2006 comparative effectiveness review. Rockville (MD): Agency for Healthcare Research and Quality (US); 2011 Oct. Report No.: 11(12)-EHC076-EF. AHRQ Comparative Effectiveness Reviews. https://europepmc.org/books/n/cer38/pdf/ |
| Chou R. Low back pain (chronic). BMJ clinical evidence. 2010;2010. https://www.ncbi.nlm.nih.gov/pmc/articles/PMC3217809/pdf/2010-1116.pdf |
| Christie A, Jamtvedt G, Dahm KT, Moe RH, Haavardsholm EA, Hagen KB. Effectiveness of nonpharmacological and nonsurgical interventions for patients with rheumatoid arthritis: an overview of systematic reviews. Physical therapy. 2007 Dec 1;87(12):1697-715. https://www.ncbi.nlm.nih.gov/pubmed/17906290 |
| Chung VC, Wu X, Hui EP, Ziea ET, Ng BF, Ho RS, Tsoi KK, Wong SY, Wu JC. Effectiveness of Chinese herbal medicine for cancer palliative care: overview of systematic reviews with meta-analyses. Scientific reports. 2015 Dec 16;5:18111. https://www.ncbi.nlm.nih.gov/pubmed/26669761 |
| Churuangsuk C, Kherouf M, Combet E, Lean M. Low‐carbohydrate diets for overweight and obesity: a systematic review of the systematic reviews. Obesity reviews. 2018 Dec;19(12):1700-18. https://www.ncbi.nlm.nih.gov/pubmed/30194696 |
| Ciapponi A, Lewin S, Herrera CA, Opiyo N, Pantoja T, Paulsen E, Rada G, Wiysonge CS, Bastias G, Dudley L, Flottorp S. Delivery arrangements for health systems in low‐income countries: an overview of systematic reviews. Cochrane Database of Systematic Reviews. 2017(9). |
| Cipriani A, Geddes JR, Furukawa TA, Barbui C. Metareview on short-term effectiveness and safety of antidepressants for depression: an evidence-based approach to inform clinical practice. The Canadian Journal of Psychiatry. 2007 Sep;52(9):553-62. https://www.ncbi.nlm.nih.gov/pubmed/17953159 |
| Clarkson P, Hughes J, Xie C, Larbey M, Roe B, Giebel CM, Jolley D, Challis D, HoSt‐D (Home Support in Dementia) Programme Management Group. Overview of systematic reviews: Effective home support in dementia care, components and impacts—Stage 1, psychosocial interventions for dementia. Journal of advanced nursing. 2017 Dec;73(12):2845-63. https://www.ncbi.nlm.nih.gov/pubmed/28621017 |
| Clifton PM, Keogh JB. A systematic review of the effect of dietary saturated and polyunsaturated fat on heart disease. Nutrition, Metabolism and Cardiovascular Diseases. 2017 Dec 1;27(12):1060-80. https://www.ncbi.nlm.nih.gov/pubmed/29174025 |
| Collins DR, Tompson AC, Onakpoya IJ, Roberts N, Ward AM, Heneghan CJ. Global cardiovascular risk assessment in the primary prevention of cardiovascular disease in adults: systematic review of systematic reviews. BMJ open. 2017 Mar 1;7(3):e013650. https://www.ncbi.nlm.nih.gov/pmc/articles/PMC5372072/ |
| Conn VS, Sells TG. Effectiveness of interventions to increase physical activity among minority populations: an umbrella review. Journal of the National Medical Association. 2016 Feb 1;108(1):54-68. https://www.ncbi.nlm.nih.gov/pubmed/26928489 |
| Connolly B, O'Neill B, Salisbury L, Blackwood B. Physical rehabilitation interventions for adult patients during critical illness: an overview of systematic reviews. Thorax. 2016 Oct 1;71(10):881-90.https://thorax.bmj.com/content/71/10/881?trendmd-shared=1&utm_source=trendmd&utm_medium=cpc&utm_campaign=thorax&utm_content=consumer&utm_term=1-AConnolly%20B,%20O%27neill%20B,%20Salisbury%20L,%20Blackwood%20B.%20Physical%20rehabilitation%20interventions%20for%20adult%20patients%20during%20critical%20illness:%20an%20overview%20of%20systematic%20reviews.%20Thorax.%202016%20Oct%201;71(10):881-90. |
| Corry M, While A, Neenan K, Smith V. A systematic review of systematic reviews on interventions for caregivers of people with chronic conditions. Journal of advanced nursing. 2015 Apr;71(4):718-34. https://onlinelibrary.wiley.com/doi/abs/10.1111/jan.12523 |
| Corsi O, Pérez-Cruz PE. Is it useful to add acetaminophen to high-potency opioids in cancer-related pain?. Medwave. 2017 May 4;17(Suppl2):e6944. https://www.medwave.cl/link.cgi/English/Updates/Epistemonikos/6945?ver=sindiseno |
| Costa AA, Vasconcellos IM, Pacheco RL, Bella ZI, Riera R. What do Cochrane systematic reviews say about non-surgical interventions for urinary incontinence in women?. Sao Paulo Medical Journal. 2018 Feb;136(1):73-83. http://www.scielo.br/scielo.php?pid=S1516-31802018000100073&script=sci_arttext |
| Costa MB, Melnik T. Effectiveness of psychosocial interventions in eating disorders: an overview of Cochrane systematic reviews. Einstein (Sao Paulo). 2016 Jun;14(2):235-77. http://www.scielo.br/scielo.php?pid=S1679-45082016000200020&script=sci_arttext |
| Cundiff DK. A systematic review of Cochrane anticoagulation reviews. The Medscape Journal of Medicine. 2009;11(1):5. https://www.ncbi.nlm.nih.gov/pmc/articles/PMC2654677/ |
| Curry N, Stanworth S, Hopewell S, Dorée C, Brohi K, Hyde C. Trauma-induced coagulopathy—a review of the systematic reviews: is there sufficient evidence to guide clinical transfusion practice?. Transfusion medicine reviews. 2011 Jul 1;25(3):217-31. https://www.sciencedirect.com/science/article/abs/pii/S0887796311000022 |
| Curtis S, Wingert A, Ali S. The Cochrane Library and procedural pain in children: an overview of reviews. Evidence‐Based Child Health: A Cochrane Review Journal. 2012 Sep;7(5):1363-99. https://onlinelibrary.wiley.com/doi/abs/10.1002/ebch.1864 |
| da Silva Lopes K, Ota E, Shakya P, Dagvadorj A, Balogun OO, Peña-Rosas JP, et al. Effects of nutrition interventions during pregnancy on low birth weight: an overview of systematic reviews. BMJ Global Health. 2017;2(3):e000389. https://gh.bmj.com/content/2/3/e000389?cpetoc=&utm_source=trendmd&utm_medium=cpc&utm_campaign=bmjcr&utm_content=consumer&utm_term=1-B |
| Daka Q, Trkulja V. Efficacy and tolerability of monocompound topical treatments for reduction of intraocular pressure in patients with primary open angle glaucoma or ocular hypertension: an overview of reviews. Croatian medical journal. 2014 Oct 15;55(5):468-80. file:///Users/student/Downloads/cmj_55_5_daka_25358880.pdf |
| Damery S, Flanagan S, Combes G. Does integrated care reduce hospital activity for patients with chronic conditions? An umbrella review of systematic reviews. BMJ Open. 2016;6:e011952. |
| Das JK, Salam RA, Arshad A, Finkelstein Y, Bhutta ZA. Interventions for adolescent substance abuse: An overview of systematic reviews. Journal of Adolescent Health. 2016 Oct 1;59(4):S61-75. https://www.sciencedirect.com/science/article/pii/S1054139X16301677 |
| Davari P, Hsiao HH, Fazel N. Mucosal lichen planus: an evidence-based treatment update. American journal of clinical dermatology. 2014 Jul 1;15(3):181-95. https://link.springer.com/content/pdf/10.1007/s40257-014-0068-6.pdf |
| David AS, Adams C. Depot antipsychotic medication in the treatment of patients with schizophrenia: (1) meta-review; (2) patient and nurse attitudes. Health Technol Assess. 2001;5(34):1-61. https://www.ncbi.nlm.nih.gov/pubmed/11809126 |
| Dawson-Hahn EE, Mickan S, Onakpoya I, Roberts N, Kronman M, Butler CC, Thompson MJ. Short-course versus long-course oral antibiotic treatment for infections treated in outpatient settings: a review of systematic reviews. Family practice. 2017 Sep 1;34(5):511-9. https://academic.oup.com/fampra/article/34/5/511/3806591 |
| de Almeida CG, Moura MD, Barberato-Filho S, Del Fiol FD, Motta RH, de Cássia Bergamaschi C. Rapid Tranquilization for Psychiatric Patients with Psychomotor Agitation: What is Known About it?. Psychiatric Quarterly. 2017 Dec 1;88(4):885-95. https://link.springer.com/article/10.1007/s11126-017-9504-0 |
| de Rezende LF, de Sá TH, Markozannes G, Rey-López JP, Lee IM, Tsilidis KK, Ioannidis JP, Eluf-Neto J. Physical activity and cancer: an umbrella review of the literature including 22 major anatomical sites and 770 000 cancer cases. Br J Sports Med. 2018 Jul 1;52(13):826-33. https://bjsm.bmj.com/content/bjsports/52/13/826.full.pdf?casa_token=2Rhk2rAnHf0AAAAA:-oiqFW5IMsRw_SxiBdyuPObZWktwaxSvJ7bd6_ryJSJxizJhVTNzs-AuI3A0cEpNC1kLx-_IfA |
| de Rezende LF, Lopes MR, Rey-Lopez JP, Matsudo VK, do Carmo Luiz O. Sedentary behavior and health outcomes: an overview of systematic reviews. PloS one. 2014;9(8). https://www.ncbi.nlm.nih.gov/pmc/articles/PMC4140795/ |
| De Spiegeleer A, Beckwée D, Bautmans I, Petrovic M; Sarcopenia Guidelines Development group of the Belgian Society of Gerontology and Geriatrics (BSGG). Pharmacological Interventions to Improve Muscle Mass, Muscle Strength and Physical Performance in Older People: An Umbrella Review of Systematic Reviews and Meta-analyses. Drugs Aging. 2018 Aug;35(8):719-734. https://lirias.kuleuven.be/retrieve/523483 |
| Derry CJ, Derry S, Moore RA. Sumatriptan (all routes of administration) for acute migraine attacks in adults‐overview of Cochrane reviews. Cochrane Database of Systematic Reviews. 2014(5). https://www.cochranelibrary.com/cdsr/doi/10.1002/14651858.CD009108.pub2/abstract |
| Derry S, Wiffen PJ, Kalso EA, Bell RF, Aldington D, Phillips T, et al. Topical analgesics for acute and chronic pain in adults - an overview of Cochrane Reviews. The Cochrane database of systematic reviews. 2017;5:CD008609- |
| Dickinson C, Dow J, Gibson G, Hayes L, Robalino S, Robinson L. Psychosocial intervention for carers of people with dementia: What components are most effective and when? A systematic review of systematic reviews. International psychogeriatrics. 2017 Jan;29(1):31-43. https://www.cambridge.org/core/journals/international-psychogeriatrics/article/psychosocial-intervention-for-carers-of-people-with-dementia-what-components-are-most-effective-and-when-a-systematic-review-of-systematic-reviews/68FBC544D2EDE2A85C08FDBA8BDC8A26 |
| Diepgen TL, Andersen KE, Chosidow O, Coenraads PJ, Elsner P, English J, Fartasch M, Gimenez‐Arnau A, Nixon R, Sasseville D, Agner T. Guidelines for diagnosis, prevention and treatment of hand eczema. JDDG: Journal der Deutschen Dermatologischen Gesellschaft. 2015 Jan;13(1):e1-22. https://onlinelibrary.wiley.com/doi/pdf/10.1111/ddg.12510_1?casa_token=rZI3D3TBqyUAAAAA:IbrQaPVRBBQ_NDC_zVur7maJYoMcRcOAjHyCOP7vg8Fvd0YJUDxPuBWPUhm3pGxjWiWu2MdXbNu3eao |
| Doherty C, Bleakley C, Delahunt E, Holden S. Treatment and prevention of acute and recurrent ankle sprain: an overview of systematic reviews with meta-analysis. Br J Sports Med. 2017 Jan 1;51(2):113-25. https://bjsm.bmj.com/content/51/2/113.short |
| Doll MK, Winters N, Boikos C, Kraicer-Melamed H, Gore G, Quach C. Safety and effectiveness of neuraminidase inhibitors for influenza treatment, prophylaxis, and outbreak control: a systematic review of systematic reviews and/or meta-analyses. Journal of Antimicrobial Chemotherapy. 2017 Nov 1;72(11):2990-3007. https://watermark.silverchair.com/dkx271.pdf?token=AQECAHi208BE49Ooan9kkhW_Ercy7Dm3ZL_9Cf3qfKAc485ysgAAAj0wggI5BgkqhkiG9w0BBwagggIqMIICJgIBADCCAh8GCSqGSIb3DQEHATAeBglghkgBZQMEAS4wEQQMLQxB_aniNnz2x2XoAgEQgIIB8H9Pamy0Vo3-KyY-9lqqzPeEqIp0koBkVJsTy73HQ79t5SJ6PlS9vimlkLw00A9p5s9_NVE3gtYNdXssiDZF4w989oHoaZ3yyseTf7wAYfJ-kRtJM28QYSOgi-KeVcSRM1baja72E6-mfpIRQvQUgCedLnVzrXVjkydxz08U9xGawZURKl7mMedkdn64behASEG7iitjjkvgMUGzBTpI3Zx9cYWyX1ielsTo5FjBdWmoPSY3-buMJ4gDCLN9ThnDV-1aHKnRgHEwUnH9GvTP0OJVOViRJThbA2wlyuThN43Cg-aVn9zgGxZXVfoppTE5uWCeqf8hq61xhtGjvi-Hi2xwxqP8Vc1frugEKvXRhQZaNncE47sjCjrSFbcrvif0nVW7K4r_NTTYKYd3_Hl_J40QMNyYRLcidSeworwF7pYB95Zv_6XwaUFiFCdBjvhHC7JG30axkXOPq53nWAG6i8s2SjQxJ9_me-FguGDnnI35FVLR4eVlKK2rpXKvhJLR7J941_S2rjTYxKtH72slLJh5xgUKVenc5Mw3svpDgcOAHBbq4-0etvEe9bO2kbL8BGyJyqRssTC67aYb_ZkISHDpAAjiBXbHCu73Rw9erLKoq_NnJRNXP-wsTgFTk0s6MS7WxPR9w1L7IeBN4x65MEw |
| Donnan J, Walsh S, Fortin Y, Gaskin J, Sikora L, Morrissey A, Collins K, MacDonald D. Factors associated with the onset and progression of neurotrauma: A systematic review of systematic reviews and meta-analyses. Neurotoxicology. 2017 Jul 1;61:234-41. https://doi.org/10.1016/j.neuro.2016.03.006 |
| Dosenovic S, Jelicic Kadic A, Miljanovic M, Biocic M, Boric K, Cavar M, Markovina N, Vucic K, Puljak L. Interventions for neuropathic pain: an overview of systematic reviews. Anesthesia & Analgesia. 2017 Aug 1;125(2):643-52. https://www.ingentaconnect.com/content/wk/ane/2017/00000125/00000002/art00041 |
| Dragioti E, Evangelou E, Larsson B, Gerdle B, Medicinska fakulteten, Region Östergötland, et al. Effectiveness of multidisciplinary programmes for clinical pain conditions: An umbrella review. Journal of rehabilitation medicine. 2018;50(9):779-91 |
| Dragioti E, Karathanos V, Gerdle B, Evangelou E. Does psychotherapy work? An umbrella review of meta‐analyses of randomized controlled trials. Acta Psychiatrica Scandinavica. 2017 Sep;136(3):236-46. https://onlinelibrary.wiley.com/doi/abs/10.1111/acps.12713 |
| Driot D, Bismuth M, Maurel A, Soulie-Albouy J, Birebent J, Oustric S, et al. Management of first depression or generalized anxiety disorder episode in adults in primary care: A systematic metareview. La Presse Médicale. 2017;46(12):1124-38. https://www.researchgate.net/profile/Damien_Driot/publication/321122574_Management_of_first_depression_or_generalized_anxiety_disorder_episode_in_adults_in_primary_care_A_systematic_metareview/links/5a1f27a80f7e9b9d5e027d3e/Management-of-first-depression-or-generalized-anxiety-disorder-episode-in-adults-in-primary-care-A-systematic-metareview.pdf |
| Duncan M, Moschopoulou E, Herrington E, Deane J, Roylance R, Jones L, Bourke L, Morgan A, Chalder T, Thaha MA, Taylor SC. Review of systematic reviews of non-pharmacological interventions to improve quality of life in cancer survivors. BMJ open. 2017 Nov 1;7(11):e015860. https://bmjopen.bmj.com/content/7/11/e015860?rss=1&int_source=trendmd&int_medium=trendmd&int_campaign=trendmd |
| Dyer SM, Harrison SL, Laver K, Whitehead C, Crotty M. An overview of systematic reviews of pharmacological and non-pharmacological interventions for the treatment of behavioral and psychological symptoms of dementia. International psychogeriatrics. 2018 Mar;30(3):295-309. https://www.cambridge.org/core/journals/international-psychogeriatrics/article/an-overview-of-systematic-reviews-of-pharmacological-and-nonpharmacological-interventions-for-the-treatment-of-behavioral-and-psychological-symptoms-of-dementia/DCA87B8BC78047977CB92427BF3F4FC3 |
| Eden JA. Phytoestrogens for menopausal symptoms: a review. Maturitas. 2012 Jun 1;72(2):157-9. |
| El Dib RP, Atallah ÁN. Evidence-based speech, language and hearing therapy and the Cochrane Library's systematic reviews. Sao Paulo Medical Journal. 2006;124(2):51-4. http://dx.doi.org/10.1590/S1516-31802006000200001 |
| Els C, Jackson TD, Hagtvedt R, Kunyk D, Sonnenberg B, Lappi VG, Straube S. High‐dose opioids for chronic non‐cancer pain: an overview of Cochrane Reviews. Cochrane Database of Systematic Reviews. 2017(10). https://www.cochranelibrary.com/cdsr/doi/10.1002/14651858.CD012299.pub2/abstract |
| Els C, Jackson TD, Kunyk D, Lappi VG, Sonnenberg B, Hagtvedt R, Sharma S, Kolahdooz F, Straube S. Adverse events associated with medium‐and long‐term use of opioids for chronic non‐cancer pain: an overview of Cochrane Reviews. Cochrane Database of Systematic Reviews. 2017(10). https://www.cochranelibrary.com/cdsr/doi/10.1002/14651858.CD012509.pub2/abstract |
| Erickson BJ, Mascarenhas R, Sayegh ET, Saltzman B, Verma NN, Bush-Joseph CA, Cole BJ, Bach Jr BR. Does operative treatment of first-time patellar dislocations lead to increased patellofemoral stability? A systematic review of overlapping meta-analyses. Arthroscopy: The Journal of Arthroscopic & Related Surgery. 2015 Jun 1;31(6):1207-15. https://www.semanticscholar.org/paper/Does-Operative-Treatment-of-First-Time-Patellar-to-Erickson-Mascarenhas/7ba56950b577c286f91aab097b9e712fe5860c7c |
| Ernst E, Lee MS, Choi TY. Acupuncture for depression? A systematic review of systematic reviews. Evaluation & the health professions. 2011 Dec;34(4):403-12. https://journals.sagepub.com/doi/abs/10.1177/0163278710386109 |
| Ernst E, Lee MS, Choi TY. Acupuncture for insomnia? An overview of systematic reviews. The European journal of general practice. 2011 Jun 1;17(2):116-23. https://www.tandfonline.com/doi/full/10.3109/13814788.2011.568475 |
| Ernst E, Lee MS, Choi TY. Acupuncture in obstetrics and gynecology: an overview of systematic reviews. The American journal of Chinese medicine. 2011;39(03):423-31. https://www.worldscientific.com/doi/abs/10.1142/S0192415X11008920 |
| Ernst E, Lee MS, Choi TY. Acupuncture: does it alleviate pain and are there serious risks? A review of reviews. PAIN®. 2011 Apr 1;152(4):755-64. https://www.sciencedirect.com/science/article/pii/S0304395910006895 |
| Ernst E, Lee MS. Acupressure: An Overview of Systematic Reviews. Journal of Pain and Symptom Management. 2010;40(4). https://www.jpsmjournal.com/article/S0885-3924(10)00539-7/fulltext. |
| Ernst E, Lee MS. Acupuncture for rheumatic conditions: an overview of systematic reviews. Rheumatology. 2010 Jun 29;49(10):1957-61. https://academic.oup.com/rheumatology/article/49/10/1957/1775761 |
| Ernst E, Posadzki P, Lee MS. Complementary and alternative medicine (CAM) for sexual dysfunction and erectile dysfunction in older men and women: an overview of systematic reviews. Maturitas. 2011 Sep 1;70(1):37-41. https://www.sciencedirect.com/science/article/abs/pii/S0378512211002106 |
| Ernst E, Posadzki P. Complementary and alternative medicine for rheumatoid arthritis and osteoarthritis: an overview of systematic reviews. Current pain and headache reports. 2011 Dec 1;15(6):431-7. https://link.springer.com/article/10.1007/s11916-011-0227-x |
| Ernst E., Lee MS. Acupuncture for palliative and supportive cancer care: a systematic review of systematic reviews. 2010;40(1):3-5. https://www.jpsmjournal.com/article/S0885-3924(10)00321-0/fulltext. |
| Eskicioglu C, Forbes SS, Fenech DS, McLeod RS, Best Practice in General Surgery Committee. Preoperative bowel preparation for patients undergoing elective colorectal surgery: a clinical practice guideline endorsed by the Canadian Society of Colon and Rectal Surgeons. Canadian Journal of Surgery. 2010 Dec;53(6):385. https://www.ncbi.nlm.nih.gov/pmc/articles/PMC2993029/ |
| Ezzo J, Streitberger K, Schneider A. Cochrane systematic reviews examine P6 acupuncture-point stimulation for nausea and vomiting. Journal of Alternative & Complementary Medicine. 2006 Jun 1;12(5):489-95. https://www.liebertpub.com/doi/abs/10.1089/acm.2006.12.489 |
| F Galvao T, Araujo EA, P Penha A, T Silva M. Statins for early stage chronic kidney disease: An overview of reviews. Cardiovascular & Haematological Disorders-Drug Targets (Formerly Current Drug Targets-Cardiovascular & Hematological Disorders). 2014 Dec 1;14(3):205-11. https://www.ncbi.nlm.nih.gov/pubmed/24720455 |
| Farquhar C, Marjoribanks J. Assisted reproductive technology: an overview of Cochrane Reviews. Cochrane Database of Systematic Reviews. 2018(8). https://www.cochranelibrary.com/cdsr/doi/10.1002/14651858.CD010537.pub5/abstract |
| Favejee MM, Huisstede BM, Koes BW. Frozen shoulder: the effectiveness of conservative and surgical interventions—systematic review. British journal of sports medicine. 2011 Jan 1;45(1):49-56. https://bjsm.bmj.com/content/bjsports/45/1/49.full.pdf?casa_token=ZmY3-ckx2tIAAAAA:h82cmL92bTCcqC1YPTc56CbnNtSesGbKlEmZ3qUj9F0eVWzuRZOQiKD2-ot6h0j3enMVmbO3vD7wpw |
| Felder-Puig, R., Piso, B., Guba, B., Gartlehner, G. Kyphoplasty and vertebroplasty for the management of osteoporotic vertebral compression fractures: a systematic review. Orthopäde. 2009;38(7):606-615. https://link-springer-com.ezproxy.library.ubc.ca/content/pdf/10.1007/s00132-009-1446-2.pdf |
| Filiatreault S, Hodgins M, Witherspoon R. An umbrella review of clinical practice guidelines for the management of patients with hip fractures and a synthesis of recommendations for the pre-operative period. J Adv Nurs. 2018 Jun;74(6):1278-1288. https://onlinelibrary.wiley.com/doi/pdf/10.1111/jan.13550?casa_token=QlhlI7tjjq0AAAAA:fnJzS8wusOpToJzgMiCkcTKBZyUeqZWDHeCNISonYYSMWjFl7u7nquNeDcgU8T0RXUeFFazmZdxhldk |
| Finelli A, Ismaila N, Bro B, Durack J, Eggener S, Evans A, Gill I, Graham D, Huang W, Jewett MA, Latcha S. Management of small renal masses: American Society of Clinical Oncology clinical practice guideline. Journal of Clinical Oncology. 2017 Jan 17;35(6):668-80. https://ascopubs.org/doi/full/10.1200/jco.2016.69.9645 |
| Flanagan S, Damery S, Combes G. The effectiveness of integrated care interventions in improving patient quality of life (QoL) for patients with chronic conditions. An overview of the systematic review evidence. Health and Quality of Life Outcomes. 2017;15(1). https://hqlo.biomedcentral.com/articles/10.1186/s12955-017-0765-y |
| Flodgren G, Eccles MP, Shepperd S, Scott A, Parmelli E, Beyer FR. An overview of reviews evaluating the effectiveness of financial incentives in changing healthcare professional behaviours and patient outcomes. Cochrane database of systematic reviews. 2011(7). |
| Flume, P.A. et al. Cystic fibrosis pulmonary guidelines: chronic medications for maintenance of lung health. American Journal of Respiratory and Critical Care Medicine. 2007;176(10):957-969. https://www.atsjournals.org/doi/pdf/10.1164/rccm.200705-664OC |
| Fong, K. M., Yang, I. A., Zimmerman, P. V. and Bowman, R. V. Cochrane systematic reviews of treatments for lung cancer. Respiratory Medicine. 2005;99(9):1071-1078. https://pdf.sciencedirectassets.com/272432/1-s2.0-S0954611105X01627/1-s2.0-S0954611105002027/main.pdf?X-Amz-Security-Token=AgoJb3JpZ2luX2VjEFoaCXVzLWVhc3QtMSJIMEYCIQCbFTu92pv5aP2DqMJCBTUk6Hqe3WsT60y6oy5PRplIHwIhAMPk4h3ehXJtGTVxmbU2TmxZaFX8SNGHN1COAihNWuKmKuMDCOP%2F%2F%2F%2F%2F%2F%2F%2F%2F%2FwEQAhoMMDU5MDAzNTQ2ODY1IgxLwIHZEZOTOtbZEogqtwP2qhNHfMQwlKxqHaqLjgxCrb7Uw%2F%2B0Vexpcu9rA8RmRRTr4mpeyMYeHAa7Fv%2B5GiST00jTVrdIIPuigCGUqO7LL%2F%2BrcZVi%2F6ZfeYcGzDNgYYA3d0oYEcg1qdsMSRk09dN6hIeRl8RUAjceZOCupRE8upymfXuQ7DbxkgR1JU05rRyS4zF9Qifd9sURcLAwx7fnZU4Kn6kTx0fYqJeN541EBWLrKndoxaF%2BeFJyjh1sqgR3or%2B5X4ccoM%2F0P6Dvrdyvl4lhC4RRH5JNVI3WBh%2FLgIQn3%2Bt8LWNaI4vJXoJPesmqUrnQWgyHsQPu%2FpY6FLWSnnBq7tOcl1YXuH1p0%2FDENS0JRU%2FQ9nv4Z2LBTR8GnmpIIUW1SM2pzLqI3ZtWdwraM7D9F8fxEOkEmQ3NVEW%2BbAvqutTknJLUlQFsKFMDZPKdEJ7L6jxV0BxNpvP12kJpGDuSQQ1vmtpsCaaNaHyF4qGkhbQ8wJ1NWDZfK5GPflj%2FMUt6NRZIITzceAn1hacPDSuVODl12%2F3TCllT0QWNeqyCkrG%2BUDNKG621ne0%2FRPRlUeziuXPVIl8yRFWTgd3DrxGKRPWoMJqpyOoFOrMBJtqYMV5S6ZGO4Eg82%2FpkqLSSm%2FkvAJktMAzXO%2F%2BhsfFJr4%2BRfPvIKKD4EnxSskI6eqFq3GEzSh5QtSi1onm86W9VucR7mvIM9eJ2T5dZfz%2F4h8JVmPYA4wAa5ntWYXQ1CyLKJR6emUO59jE4HQ4GwxZmTTIyrccn%2B4oQIdOgW8RrgvhLz6djLaspdHqxtL4axvM%2F%2Bl0xsp7OQl6EhWL9YhpREbJvJFuMGuieDo%2B9j5WVKXE%3D&X-Amz-Algorithm=AWS4-HMAC-SHA256&X-Amz-Date=20190813T024631Z&X-Amz-SignedHeaders=host&X-Amz-Expires=300&X-Amz-Credential=ASIAQ3PHCVTYQN5J2CCH%2F20190813%2Fus-east-1%2Fs3%2Faws4_request&X-Amz-Signature=c7f1354bd4bc246ac8f4ab9491afad34ef3c7fc211e6c8eefd62436a3f800087&hash=d3db9082269997a6d8c98c30b4eec37a81d8dccdf3267259a69b51ad9f99c443&host=68042c943591013ac2b2430a89b270f6af2c76d8dfd086a07176afe7c76c2c61&pii=S0954611105002027&tid=spdf-b8c6707d-2f16-4744-92d3-3596db3f98fe&sid=8d1ff7d09397a24a3d48ae59e0546065b089gxrqa&type=client |
| Fornaro M, De Berardis D, Perna G, Solmi M, Veronese N, Orsolini L, Buonaguro EF, Iasevoli F, Köhler CA, Carvalho AF, De Bartolomeis A. Lurasidone in the treatment of bipolar depression: systematic review of systematic reviews. BioMed research international. 2017;2017. http://downloads.hindawi.com/journals/bmri/2017/3084859.pdf |
| Foroushani PS, Schneider J, Assareh N. Meta-review of the effectiveness of computerised CBT in treating depression. BMC psychiatry. 2011 Dec;11(1):131. https://bmcpsychiatry.biomedcentral.com/articles/10.1186/1471-244X-11-131 |
| Fortin PM, Hopewell S, Estcourt LJ. Red blood cell transfusion to treat or prevent complications in sickle cell disease: an overview of Cochrane reviews. Cochrane Database of Systematic Reviews. 2018(8). https://www.cochranelibrary.com/cdsr/doi/10.1002/14651858.CD012082.pub2/abstract |
| Freedman SB, Ali S, Oleszczuk M, Gouin S, Hartling L. Treatment of acute gastroenteritis in children: an overview of systematic reviews of interventions commonly used in developed countries. Evidence‐Based Child Health: A Cochrane Review Journal. 2013 Jul;8(4):1123-37. https://onlinelibrary.wiley.com/doi/abs/10.1002/ebch.1932 |
| French DP, Cameron E, Benton JS, Deaton C, Harvie M. Can communicating personalised disease risk promote healthy behaviour change? A systematic review of systematic reviews. Annals of Behavioral Medicine. 2017 Mar 13;51(5):718-29. https://academic.oup.com/abm/article/51/5/718/4648590 |
| Fu L, Hu Y, Lu HZ. Overviews of reviews on patient compliance with medication protocols used in highly active antiretroviral therapy. International Journal of Nursing Sciences. 2015 Mar 1;2(1):61-5. https://www.sciencedirect.com/science/article/pii/S2352013215000216 |
| Furlan, A. D., Clarke, J., Esmail, R., Sinclair, S., Irvin, E. and Bombardier, C. A critical review of reviews on the treatment of chronic low back pain. Spine. 2001;26:E155–E162 https://ovidsp-dc2-ovid-com.ezproxy.library.ubc.ca/sp-4.01.0a/ovidweb.cgi?WebLinkFrameset=1&S=ENGJFPHKMNEBNKMJJPCKJEHGCCDLAA00&returnUrl=ovidweb.cgi%3fMain%2bSearch%2bPage%3d1%26S%3dENGJFPHKMNEBNKMJJPCKJEHGCCDLAA00&directlink=https%3a%2f%2fovidsp.dc2.ovid.com%2fovftpdfs%2fFPEBJPHGJEMJMN00%2ffs035%2fovft%2flive%2fgv010%2f00007632%2f00007632-200104010-00018.pdf&filename=A+Critical+Review+of+Reviews+on+the+Treatment+of+Chronic+Low+Back+Pain.&link_from=S.sh.27%7c1&pdf_key=FPEBJPHGJEMJMN00&pdf_index=/fs035/ovft/live/gv010/00007632/00007632-200104010-00018&D=ovft |
| Gamble JM, Clarke A, Myers KJ, Agnew MD, Hatch K, Snow MM, Davis EM. Incretin‐based medications for type 2 diabetes: an overview of reviews. Diabetes, Obesity and Metabolism. 2015 Jul;17(7):649-58. http://www.siditalia.it/images/Gamble-Incretin_rev.pdf |
| Gartlehner G, Wagner G, Matyas N, Titscher V, Greimel J, Lux L, Gaynes BN, Viswanathan M, Patel S, Lohr KN. Pharmacological and non-pharmacological treatments for major depressive disorder: review of systematic reviews. BMJ open. 2017 Jun 1;7(6):e014912. |
| Geneen LJ, Moore RA, Clarke C, Martin D, Colvin LA, Smith BH. Physical activity and exercise for chronic pain in adults: an overview of Cochrane Reviews. Cochrane Database of Systematic Reviews. 2017(4). https://www.cochranelibrary.com/cdsr/doi/10.1002/14651858.CD011279.pub3/abstract |
| George PP, Molina JA, Heng BH. The methodological quality of systematic reviews comparing intravitreal bevacizumab and alternates for neovascular age related macular degeneration: A systematic review of reviews. Indian journal of ophthalmology. 2014 Jul;62(7):761. https://dx.doi.org/10.4103%2F0301-4738.138615 |
| Goldstein KM, Shepherd-Banigan M, Coeytaux RR, McDuffie JR, Adam S, Befus D, Goode AP, Kosinski AS, Masilamani V, Williams Jr JW. Use of mindfulness, meditation and relaxation to treat vasomotor symptoms. Climacteric. 2017 Mar 4;20(2):178-82. |
| Grassi, A., Compagnoni, R., Ferrua, P., Zaffagnini, S., Berruto, M., Samuelsson, K., Svantesson, E. and Randelli, P., 2018. Patellar resurfacing versus patellar retention in primary total knee arthroplasty: a systematic review of overlapping meta-analyses. Knee Surgery, Sports Traumatology, Arthroscopy, 26(11), pp.3206-3218. https://link.springer.com/article/10.1007/s00167-018-4831-8 |
| Griffiths, P. The role of cranberry juice in the treatment of urinary tract infections. British Journal of Community Nursing. 2003; 8(12):557-561. https://www.researchgate.net/profile/Peter_Griffiths3/post/What_are_the_health_benefits_of_cranberries/attachment/59d6388ac49f478072ea5779/AS%3A273701675700227%401442266972858/download/cranberry.pdf |
| Grimes DA, Lopez LM, Manion C, Schulz KF. Cochrane systematic reviews of IUD trials: lessons learned. Contraception. 2007;75(6). https://www.sciencedirect.com/science/article/abs/pii/S0010782407000054 |
| Grosso G, Godos J, Galvano F, Giovannucci EL. Coffee, caffeine, and health outcomes: an umbrella review. Annual review of nutrition. 2017 Aug 21;37:131-56. https://www.annualreviews.org/doi/abs/10.1146/annurev-nutr-071816-064941 |
| Guay J, Choi P, Suresh S, Albert N, Kopp S, Pace NL. Neuraxial blockade for the prevention of postoperative mortality and major morbidity: an overview of Cochrane systematic reviews. Cochrane Database of Systematic Reviews. 2014(1). https://www.cochranelibrary.com/cdsr/doi/10.1002/14651858.CD010108.pub2/abstract |
| Guay J, Choi PT, Suresh S, Albert N, Kopp S, Pace NL. Neuraxial anesthesia for the prevention of postoperative mortality and major morbidity: an overview of cochrane systematic reviews. Anesthesia & Analgesia. 2014 Sep 1;119(3):716-25. https://doi.org/10.1213/ANE.0000000000000339 |
| Guo X-W, Hu N-D, Sun G-Z, Li M, Zhang P-T. Shenyi Capsule (参一胶囊) plus Chemotherapy versus Chemotherapy for Non-Small Cell Lung Cancer: A Systematic Review of Overlapping Meta-Analyses. Chinese Journal of Integrative Medicine. 2018;24(3):227–31. http://web.a.ebscohost.com.ezproxy.library.ubc.ca/ehost/pdfviewer/pdfviewer?vid=1&sid=ef55f59f-1b53-40f0-b656-4a682ce387c3%40sdc-v-sessmgr01 |
| Hagen KB, Dagfinrud H, Moe RH, Østerås N, Kjeken I, Grotle M, Smedslund G. Exercise therapy for bone and muscle health: an overview of systematic reviews. BMC medicine. 2012 Dec;10(1):167. https://doi.org/10.1186/1741-7015-10-167 |
| Halliday, H. L. What interventions facilitate weaning from the ventilator? A review of the evidence from systematic reviews. Paediatric Respiratory Reviews.2004;5:S347 - S352. https://www-sciencedirect-com.ezproxy.library.ubc.ca/science/article/pii/S1526054204900607 |
| Hamill JK, Rahiri JL, Hill AG. Analgesic effect of intraperitoneal local anesthetic in surgery: an overview of systematic reviews. Journal of Surgical Research. 2017 May 15;212:167-77. https://www.sciencedirect.com/science/article/pii/S0022480417300410 |
| Hasuike A, Iguchi S, Suzuki D, Kawano E, Sato S. Systematic review and assessment of systematic reviews examining the effect of periodontal treatment on glycemic control in patients with diabetes. Medicina oral, patologia oral y cirugia bucal. 2017 Mar;22(2):e167. https://www.ncbi.nlm.nih.gov/pmc/articles/PMC5359698/pdf/medoral-22-e167.pdf |
| Häuser W, Bock F, Engeser P, Tölle T, Willweber-Strumpf A, Petzke F. Long-term opioid use in non-cancer pain. Deutsches Ärzteblatt International. 2014 Oct;111(43):732. https://www.ncbi.nlm.nih.gov/pmc/articles/PMC4238316/pdf/Dtsch_Arztebl_Int-111-0732.pdf |
| Häuser W, Petzke F, Fitzcharles MA. Efficacy, tolerability and safety of cannabis‐based medicines for chronic pain management–An overview of systematic reviews. European Journal of Pain. 2018 Mar;22(3):455-70. https://onlinelibrary.wiley.com/doi/pdf/10.1002/ejp.1118?casa_token=E6Kazeku5nYAAAAA:T78WK7_GSewpUrlBy7j-h63kxgv0_lbreBosD1xD-8ZPSasYcvY_kuvjlytqgcxRCj0pqfeemtkl |
| Health Evidence Team of the Piripiri Municipal Health Secretariat (MHS). Prevention and control of dengue fever in urban areas. 2011; MUNICIPALITY OF PIRIPIRI, MUNICIPAL HEALTH SECRETARIAT, HEALTH EVIDENCE TEAM, WHO Brasil http://www.who.int/evidence/PBPIRIPIRIDENGUE2012.pdf |
| Health Quality Ontario. Oral appliances for obstructive sleep apnea: an evidence-based analysis. Ontario health technology assessment series. 2009;9(5):1. https://www.ncbi.nlm.nih.gov/pmc/articles/PMC3377505/ |
| Health Quality Ontario. Vitamin B12 and cognitive function: an evidence-based analysis. Ontario health technology assessment series. 2013;13(23):1. |
| Heimer KA, Hart AM, Martin LG, Rubio-Wallace S. Examining the evidence for the use of vitamin C in the prophylaxis and treatment of the common cold. Journal of the American Academy of Nurse Practitioners. 2009;21(5):295–300. https://onlinelibrary-wiley-com.ezproxy.library.ubc.ca/doi/pdf/10.1111/j.1745-7599.2009.00409.x |
| Herrera CA, Lewin S, Paulsen E, Ciapponi A, Opiyo N, Pantoja T, Rada G, Wiysonge CS, Bastías G, Marti SG, Okwundu CI. Governance arrangements for health systems in low‐income countries: an overview of systematic reviews. Cochrane Database of Systematic Reviews. 2017(9). https://www.cochranelibrary.com/cdsr/doi/10.1002/14651858.CD011085.pub2/abstract |
| Hindocha, Akshay, et al. "Adhesion prevention agents for gynaecological surgery: an overview of Cochrane reviews." Cochrane Database of Systematic Reviews 1 (2015). https://www.cochranelibrary.com/cdsr/doi/10.1002/14651858.CD011254.pub2/abstract |
| Hocevar BJ, Robinson B, Gray M. Does chewing gum shorten the duration of postoperative ileus in patients undergoing abdominal surgery and creation of a stoma?. Journal of Wound Ostomy & Continence Nursing. 2010 Mar 1;37(2):140-6. https://journals.lww.com/jwocnonline/fulltext/2010/03000/Does_Chewing_Gum_Shorten_the_Duration_of.6.aspx?casa_token=SyRzZNnKcigAAAAA:mEc1i7-2LgT5d2EYtGQAr8dlkXflMD-lOPRVE4rctzaZwfrkijhhihNq29KJiECVDqFez_pi4DaVZ6WRDid8Knl1 |
| Hodgson SH, Angus BJ. Malaria: fluid therapy in severe disease. BMJ clinical evidence. 2016;2016. https://www.ncbi.nlm.nih.gov/pmc/articles/PMC4725623/pdf/2016-0913.pdf |
| Hopton A, MacPherson H. Acupuncture for chronic pain: is acupuncture more than an effective placebo? A systematic review of pooled data from meta‐analyses. Pain Practice. 2010 Mar 1;10(2):94-102. https://onlinelibrary.wiley.com/doi/abs/10.1111/j.1533-2500.2009.00337.x |
| Horkan L, Stansfield G, Miller M. An analysis of systematic reviews undertaken on standard advanced wound dressings in the last 10 years. Journal of Wound Care. 2009;18(7):298–304. http://web.b.ebscohost.com.ezproxy.library.ubc.ca/ehost/pdfviewer/pdfviewer?vid=1&sid=faa4f057-cbec-47c6-a7be-89a4386bfbd7%40pdc-v-sessmgr02 |
| Houck DA, Kraeutler MJ, Schuette HB, Mccarty EC, Bravman JT. Early Versus Delayed Motion After Rotator Cuff Repair: A Systematic Review of Overlapping Meta-analyses. The American Journal of Sports Medicine. 2017;45(12):2911–5. https://journals-sagepub-com.ezproxy.library.ubc.ca/doi/pdf/10.1177/0363546517692543 |
| Houze B, El-Khatib H, Arbour C. Efficacy, tolerability, and safety of non-pharmacological therapies for chronic pain: an umbrella review on various CAM approaches. Progress in Neuro-Psychopharmacology and Biological Psychiatry. 2017 Oct 3;79:192-205. https://www.sciencedirect.com/science/article/pii/S0278584617300866 |
| Hoving JL, Gross AR, Gasner D, Kay T, Kennedy C, Hondras MA, Haines T, Bouter LM. A critical appraisal of review articles on the effectiveness of conservative treatment for neck pain. Spine. 2001 Jan 15;26(2):196-205. https://journals.lww.com/spinejournal/Abstract/2001/01150/A_Critical_Appraisal_of_Review_Articles_on_the.15.aspx |
| Huguet A, McGrath PJ, Stinson J, Tougas ME, Doucette S. Efficacy of psychological treatment for headaches: an overview of systematic reviews and analysis of potential modifiers of treatment efficacy. The Clinical journal of pain. 2014 Apr 1;30(4):353-69. https://journals.lww.com/clinicalpain/Fulltext/2014/04000/Efficacy_of_Psychological_Treatment_for_Headaches_.11.aspx?casa_token=I51FPsim1LIAAAAA:TPPzpptzqlyUPUhkz5vbwqZYJ4vHxE2mGsYe_sipRf9FWVhm-vkGTXKjS27D2obdwI-XEtPxAX_Vc_x-QUZxbQ |
| Huhn M, Tardy M, Spineli LM, Kissling W, Förstl H, Pitschel-Walz G, Leucht C, Samara M, Dold M, Davis JM, Leucht S. Efficacy of pharmacotherapy and psychotherapy for adult psychiatric disorders: a systematic overview of meta-analyses. JAMA psychiatry. 2014 Jun 1;71(6):706-15. https://jamanetwork.com/journals/jamapsychiatry/fullarticle/1865002?casa_token=KKWqMFGxfkYAAAAA:aT2kBUdizMS1SifXRaerdqfuqE2aKCxUu4O2QtmCJwg-E4hZP4GFuJtNHg5uGypnLE-f2u9Q |
| Hunt K, Ernst E. The evidence-base for complementary medicine in children: a critical overview of systematic reviews. Archives of disease in childhood. 2011 Aug 1;96(8):769-76. https://adc.bmj.com/content/96/8/769.short |
| Ip, S., Chung, M., Raman, G., Chew, P., Magula, N., DeVine, D., Trikalinos, T. and Lau, J. Breastfeeding and maternal and infant health outcomes in developed countries. Evidence Report/Technology Assessment . 2007;153(1):1530-4396 https://www.ncbi.nlm.nih.gov/pubmed/17764214 |
| Jacobs WC, Rubinstein SM, Willems PC, Moojen WA, Pellisé F, Oner CF, Peul WC, van Tulder MW. The evidence on surgical interventions for low back disorders, an overview of systematic reviews. European Spine Journal. 2013 Sep 1;22(9):1936-49. https://idp.springer.com/authorize/casa?redirect_uri=https://link.springer.com/article/10.1007/s00586-013-2823-4&casa_token=pMrBPYSRdOAAAAAA:80EjyXbPq6P3T2xBXZw24mhF8QvX43EXc3OFF0507d2xb_IdKpcqSlT7DRud7lBCAtjTRp0ymU2fLN0 |
| Jafari M, Mousavi SM, Asgharzadeh A, Yazdani N. Coenzyme Q10 in the treatment of heart failure: A systematic review of systematic reviews. Indian heart journal. 2018 Jul 1;70:S111-7. https://www.sciencedirect.com/science/article/pii/S0019483217308696 |
| Jamtvedt G, Dahm KT, Christie A, Moe RH, Haavardsholm E, Holm I, Hagen KB. Physical therapy interventions for patients with osteoarthritis of the knee: an overview of systematic reviews. Physical therapy. 2008 Jan 1;88(1):123-36. https://academic.oup.com/ptj/article/88/1/123/2747242 |
| Jaschinski T, Mosch C, Eikermann M, Neugebauer EA. Laparoscopic versus open appendectomy in patients with suspected appendicitis: a systematic review of meta-analyses of randomised controlled trials. BMC gastroenterology. 2015 Dec;15(1):48. http://doi.org/10.1186/s12876-015-0277-3 |
| Jin L, Guo-yi G, Ji-yao J, Gui-e L. Is management of acute traumatic brain injury effective? A literature review of published Cochrane Systematic Reviews. Chinese Journal of Traumatology. 2012 Feb 1;15(1):17-22. https://www.sciencedirect.com/science/article/pii/S1008127515302571 |
| Jobst BC, Cascino GD. Resective epilepsy surgery for drug-resistant focal epilepsy: a review. Jama. 2015 Jan 20;313(3):285-93 |
| Johal A, Fleming PS, Manek S, Marinho VC. Mandibular advancement splint (MAS) therapy for obstructive sleep apnoea—an overview and quality assessment of systematic reviews. Sleep and Breathing. 2015 Sep 1;19(3):1101-8. https://doi.org/10.1007/s11325-015-1148-4 |
| Johnson R, Bryant S, Huntley AL. Green tea and green tea catechin extracts: An overview of the clinical evidence. Maturitas. 2012;73(4):280–7. https://www.maturitas.org/article/S0378-5122(12)00270-8/fulltext |
| Jones EB, Sharpe L. Cognitive bias modification: A review of meta-analyses. Journal of Affective Disorders. 2017 Dec 1;223:175-83. https://www.sciencedirect.com/science/article/abs/pii/S0165032717310960 |
| Jones L, Othman M, Dowswell T, Alfirevic Z, Gates S, Newburn M, Jordan S, Lavender T, Neilson JP. Pain management for women in labour: an overview of systematic reviews. Cochrane database of systematic reviews. 2012(3). https://www.cochranelibrary.com/cdsr/doi/10.1002/14651858.CD009234.pub2/abstract |
| Jung XT, Newton R. Cochrane Reviews of non-medication-based psychotherapeutic and other interventions for schizophrenia, psychosis, and bipolar disorder: A systematic literature review. International Journal of Mental Health Nursing. 2009;18(4):239–49. https://onlinelibrary-wiley-com.ezproxy.library.ubc.ca/doi/pdf/10.1111/j.1447-0349.2009.00613.x |
| Kamioka H, Tsutani K, Okuizumi H, Mutoh Y, Ohta M, Handa S, et al. Effectiveness of Aquatic Exercise and Balneotherapy: A Summary of Systematic Reviews Based on Randomized Controlled Trials of Water Immersion Therapies. Journal of Epidemiology. 2010;20(1):2–12. https://www.jstage.jst.go.jp/article/jea/advpub/0/advpub_JE20090030/_pdf |
| Kamper SJ, Yamato TP, Williams CM. The prevalence, risk factors, prognosis and treatment for back pain in children and adolescents: an overview of systematic reviews. Best Practice & Research Clinical Rheumatology. 2016 Dec 1;30(6):1021-36. https://www.sciencedirect.com/science/article/abs/pii/S1521694217300037 |
| Kang HS, Jeong D, Kim DI, Lee MS. The use of acupuncture for managing gynaecologic conditions: An overview of systematic reviews. Maturitas. 2011 Apr 1;68(4):346-54. sci-hub.tw/10.1016/j.maturitas.2011.02.001 |
| Karmali KN, Lloyd-Jones DM, Berendsen MA, Goff DC, Sanghavi DM, Brown NC, Korenovska L, Huffman MD. Drugs for primary prevention of atherosclerotic cardiovascular disease: an overview of systematic reviews. JAMA cardiology. 2016 Jun 1;1(3):341-9. https://jamanetwork.com/journals/jamacardiology/fullarticle/2517393 |
| Kelley GA, Kelley KS. Exercise and cancer-related fatigue in adults: a systematic review of previous systematic reviews with meta-analyses. BMC cancer. 2017 Dec 1;17(1):693. https://doi.org/10.1186/s12885-017-3687-5 |
| Kelley GA, Kelley KS. Exercise and sleep: a systematic review of previous meta‐analyses. Journal of Evidence‐Based Medicine. 2017 Feb;10(1):26-36. https://doi.org/10.1111/jebm.12236 |
| Keus F, Gooszen HG, van Laarhoven CJ. Open, small‐incision, or laparoscopic cholecystectomy for patients with symptomatic cholecystolithiasis. An overview of Cochrane Hepato‐Biliary Group reviews. Cochrane database of systematic reviews. 2010(1). https://www.cochranelibrary.com/cdsr/doi/10.1002/14651858.CD008318/abstract |
| Khan BA, Zawahiri M, Campbell NL, Fox GC, Weinstein EJ, Nazir A, Farber MO, Buckley JD, MacLullich A, Boustani MA. Delirium in hospitalized patients: implications of current evidence on clinical practice and future avenues for research—a systematic evidence review. Journal of Hospital Medicine. 2012 Sep;7(7):580-9. https://www.ncbi.nlm.nih.gov/pmc/articles/PMC3640527/pdf/452459.pdf |
| Khan F, Amatya B. Rehabilitation in multiple sclerosis: a systematic review of systematic reviews. Archives of physical medicine and rehabilitation. 2017 Feb 1;98(2):353-67. |
| Kim TH, Kang JW, Lee TH. Therapeutic options for aromatase inhibitor-associated arthralgia in breast cancer survivors: a systematic review of systematic reviews, evidence mapping, and network meta-analysis. Maturitas. 2018 Dec 1;118:29-37. https://www.ncbi.nlm.nih.gov/pubmed/30415752 |
| Kim TH, Lim HJ, Kim MS, Lee MS. Dietary supplements for benign prostatic hyperplasia: An overview of systematic reviews. Maturitas. 2012 Nov 1;73(3):180-5. https://www.sciencedirect.com/science/article/abs/pii/S037851221200240X |
| Kosseim M, Rein R, McShane C. Implementing evidence-based physiotherapy practice for treating children with low back pain: are we there yet?. Pediatric Physical Therapy. 2008 Jul 1;20(2):179-84. http://doi.org/10.1097/PEP.0b013e318172479e |
| Krishnan JA, Segal JB, Streiff MB, Bolger DT, Eng J, Jenckes MW, Tamariz LJ, Bass EB. Treatment of venous thromboembolism with low-molecular-weight heparin: a synthesis of the evidence published in systematic literature reviews. Respiratory medicine. 2004 May 1;98(5):376-86. https://www.sciencedirect.com/science/article/pii/S095461110400085X |
| Kumar A, Galeb S, Djulbegovic B. Treatment of patients with multiple myeloma: an overview of systematic reviews. Acta haematologica. 2011;125(1-2):8-22. https://www.karger.com/Article/PDF/318880 |
| Kurz, A. and Van Baelen, B. Ginkgo biloba compared with cholinesterase inhibitors in the treatment of dementia: a review based on meta-analyses by the cochrane collaboration. Dement Geriatr Cogn Disord. 2004;18:217–226. https://mediatum.ub.tum.de/doc/1218696/file.pdf |
| Kuukasjärvi, P., Malmivaara, A., Halinen, M., Hartikainen, J., Keto, P. E., Talvensaari, T., … Mäkelä, M. (2006). Overview of systematic reviews on invasive treatment of stable coronary artery disease. International Journal of Technology Assessment in Health Care, 22(02), 219–234. sci-hub.tw/10.1017/S026646230605104X |
| Kwok CS, Pang CL, Yeong JK, Loke YK. Measures used to treat contrast-induced nephropathy: overview of reviews. The British journal of radiology. 2013 Jan;86(1021):20120272-. https://doi.org/10.1259/bjr.20120272 |
| Labre M, Phd P, Mph M, Herman E, Md M, Mph M, et al. Public Health Interventions for Asthma, An Umbrella Review, 1990–2010. SciVee. 2012; https://www.ajpmonline.org/article/S0749-3797%2812%2900023-2/fulltext. |
| Lake R, Georgiou A, Li J, Li L, Byrne M, Robinson M, et al. The quality, safety and governance of telephone triage and advice services – an overview of evidence from systematic reviews. BMC Health Services Research. 2017;17(1). https://bmchealthservres.biomedcentral.com/articles/10.1186/s12913-017-2564-x |
| Langhorst J, Heldmann P, Henningsen P, Kopke K, Krumbein L, Lucius H, Winkelmann A, Wolf B, Haeuser W. Complementary and alternative procedures for fibromyalgia syndrome: Updated guidelines 2017 and overview of systematic review articles. Schmerz (Berlin, Germany). 2017 Jun;31(3):289-95. https://europepmc.org/abstract/med/28493227 |
| Laranjeira FO, de Andrade KR, Figueiredo AC, Silva EN, Pereira MG. Long-acting insulin analogues for type 1 diabetes: An overview of systematic reviews and meta-analysis of randomized controlled trials. PloS one. 2018 Apr 12;13(4):e0194801. https://journals.plos.org/plosone/article?id=10.1371/journal.pone.0194801 |
| Lassi, Z. S., Moin, A., Das, J. K., Salam, R. A. and Bhutta, Z. A. Systematic review on evidence-based adolescent nutrition interventions. https://nyaspubs.onlinelibrary.wiley.com/doi/full/10.1111/nyas.13335 |
| Latthe PM, Foon R, Khan K. Nonsurgical treatment of stress urinary incontinence (SUI): grading of evidence in systematic reviews. BJOG: An International Journal of Obstetrics & Gynaecology. 2008 Mar;115(4):435-44. |
| Lee C, Crawford C, Wallerstedt D, York A, Duncan A, Smith J, Sprengel M, Welton R, Jonas W. The effectiveness of acupuncture research across components of the trauma spectrum response (tsr): a systematic review of reviews. Systematic reviews. 2012 Dec;1(1):46. https://systematicreviewsjournal.biomedcentral.com/articles/10.1186/2046-4053-1-46 |
| Lee MS, Choi J, Posadzki P, Ernst E. Aromatherapy for health care: an overview of systematic reviews. Maturitas. 2012 Mar 1;71(3):257-60. https://www.sciencedirect.com/science/article/abs/pii/S0378512212000060 |
| Lee MS, Ernst E. Acupuncture for pain: an overview of Cochrane reviews. Chinese Journal of Integrative Medicine. 2011 Mar 1;17(3):187-9. https://link.springer.com/article/10.1007/s11655-011-0665-7 |
| Lee MS, Ernst E. Acupuncture for surgical conditions: an overview of systematic reviews. International journal of clinical practice. 2014 Jun;68(6):783-9. https://onlinelibrary.wiley.com/doi/pdf/10.1111/ijcp.12372 |
| Lee MS, Kim J-I, Ernst E. Is Cupping an Effective Treatment? An Overview of Systematic Reviews. Journal of Acupuncture and Meridian Studies. 2011;4(1):1–4. https://www.sciencedirect.com/science/article/pii/S2005290111600010 |
| Lee-anne SC, Lithander FE, Gruen RL, Williams LT. Nutrition therapy in the optimisation of health outcomes in adult patients with moderate to severe traumatic brain injury: findings from a scoping review. Injury. 2014 Dec 1;45(12):1834-41. file:///C:/Users/Stephen/Documents/Costello%202014.pdf |
| Legere LE, McNeill S, Schindel Martin L, Acorn M, An D. Nonpharmacological approaches for behavioural and psychological symptoms of dementia in older adults: A systematic review of reviews. Journal of clinical nursing. 2018 Apr;27(7-8):e1360-76. https://onlinelibrary.wiley.com/doi/pdf/10.1111/jocn.14007?casa_token=80LiECZbWhAAAAAA:miOHFLSB2vru_LCpWwqcVigAooYrl9uplEWwMyV65SehkHWQTzpj1UsIotSt3q7l3IuIt8XF5V6I |
| Leibovici L, Soares-Weiser K, Paul M, Goldberg E, Herxheimer A, Garner P. Considering resistance in systematic reviews of antibiotic treatment. J Antimicrob Chemother. 2003 Oct;52(4):564-71. https://academic.oup.com/jac/article/52/4/564/713752 |
| Lemmens V, Oenema A, Knut IK, Brug J. Effectiveness of smoking cessation interventions among adults: a systematic review of reviews. European journal of cancer prevention. 2008 Nov 1;17(6):535-44. https://journals.lww.com/eurjcancerprev/Abstract/2008/11000/Effectiveness_of_smoking_cessation_interventions.6.aspx |
| Lepore SJ, Coyne JC. Psychological interventions for distress in cancer patients: a review of reviews. Annals of Behavioral Medicine. 2006 Oct 1;32(2):85-92. http://www.academia.edu/download/35932252/Paper_2_Lepore___Coyne_Argument_1.pdf |
| Leucht S, Hierl S, Kissling W, Dold M, Davis JM. Putting the efficacy of psychiatric and general medicine medication into perspective: review of meta-analyses. The British Journal of Psychiatry. 2012 http://m.ecnp-congress.eu/~/media/Files/ecnp/communication/talk-of-the-month/Leucht/Leucht%20Br%20J%20Psychiatry%20Putting%20the%20efficacy%20in%20perspective%202012.pdfFeb;200(2):97-106. |
| Lewin S, Lavis JN, Oxman AD, Bastías G, Chopra M, Ciapponi A, Flottorp S, Martí SG, Pantoja T, Rada G, Souza N. Supporting the delivery of cost-effective interventions in primary health-care systems in low-income and middle-income countries: an overview of systematic reviews. The Lancet. 2008 Sep 13;372(9642):928-39. https://www.sciencedirect.com/science/article/pii/S0140673608614038 |
| Li H, Xing D, Ke Y, Lin J. Safety of intra‐articular steroid injections prior to arthroplasty: Best evidence selection and risk of bias considerations. International journal of rheumatic diseases. 2018 May;21(5):982-91. https://onlinelibrary.wiley.com/doi/pdf/10.1111/1756-185X.13314?casa_token=jGTmm95eohYAAAAA:41O5VujTa8V4SX5sSvhBL81pbVJg1eOvuzmRZJOzoDR2JkCrZHiXGNXITlRS22ENaUROoiOI8bop |
| Li W, Wan Y, Ren J, Li T, Li C. Appraisal of the methodological quality and summary of the findings of systematic reviews on the relationship between SSRIs and suicidality. Shanghai archives of psychiatry. 2014;26(5):248. |
| Li X, Meng X, Timofeeva M, Tzoulaki I, Tsilidis KK, Ioannidis JP, Campbell H, Theodoratou E. Serum uric acid levels and multiple health outcomes: umbrella review of evidence from observational studies, randomised controlled trials, and Mendelian randomisation studies. Bmj. 2017 Jun 7;357:j2376. https://www.bmj.com/content/357/bmj.j2376 |
| Lindenmeyer LP, Hegele V, Caregnato JP, Wüst D, Grazziotin L, Stoll P. Follow-up of patients receiving rituximab for diffuse large B cell lymphoma: an overview of systematic reviews. Annals of hematology. 2013 Nov 1;92(11):1451-9. |
| Liu XL, Shi Y, Willis K, Wu CJ, Johnson M. Health education for patients with acute coronary syndrome and type 2 diabetes mellitus: an umbrella review of systematic reviews and meta-analyses. BMJ open. 2017 Oct 1;7(10):e016857. http://dx.doi.org/10.1136/bmjopen-2017-016857 |
| Liu Z, Dumville JC, Norman G, Westby MJ, Blazeby J, McFarlane E, Welton NJ, O'Connor L, Cawthorne J, George RP, Crosbie EJ, Rithalia AD, Cheng HY. Intraoperative interventions for preventing surgical site infection: an overview of Cochrane Reviews. Cochrane Database Syst Rev. 2018 Feb 6;2:CD012653  https://www.ncbi.nlm.nih.gov/pmc/articles/PMC6491077/ |
| Lockwood C, Stern C. Interventions for the treatment of trachoma: An overview of Cochrane systematic reviews: Trachoma: Overview of Cochrane evidence. International Journal of Nursing Practice. 2014;20(6):709-21. |
| Lodge C, Allen K, Lowe A, Dharmage S. Overview of evidence in prevention and aetiology of food allergy: a review of systematic reviews. International journal of environmental research and public health. 2013 Nov;10(11):5781-806. https://www.mdpi.com/1660-4601/10/11/5781 |
| Loh SY, Musa AN. Methods to improve rehabilitation of patients following breast cancer surgery: a review of systematic reviews. Breast Cancer: Targets and Therapy. 2015;7:81. https://www.ncbi.nlm.nih.gov/pmc/articles/PMC4360828/ |
| Long H, Lin Z, Lu B, Zhao R, Sun B, Cheng L, Zhao S, Zhu Y. Percutaneous compression plate versus dynamic hip screw for treatment of intertrochanteric hip fractures: a overview of systematic reviews and update meta-analysis of randomized controlled trials. International Journal of Surgery. 2016 Sep 1;33:1-7. https://www.sciencedirect.com/science/article/pii/S1743919116302126 |
| Long J, Briggs M, Astin F. Overview of Systematic Reviews of Mindfulness Meditation-based Interventions for People With Long-term Conditions. Advances in mind-body medicine. 2017;31(4):26-36. https://www.researchgate.net/publication/322315725_Overview_of_Systematic_Reviews_of_Mindfulness_Meditation-based_Interventions_for_People_With_Long-term_Conditions/link/5aab90f2a6fdccd3b9bc70e9/download |
| Long L, Briscoe S, Cooper C, Hyde C, Crathorne L. What is the clinical effectiveness and cost-effectiveness of conservative interventions for tendinopathy? An overview of systematic reviews of clinical effectiveness and systematic review of economic evaluations. Health Technol Assess. 2015;19(8):1-134. https://www.ncbi.nlm.nih.gov/books/NBK269589/ |
| López NJ, Uribe S, Martinez B. Effect of periodontal treatment on preterm birth rate: a systematic review of meta‐analyses. Periodontology 2000. 2015 Feb;67(1):87-130. https://www.ncbi.nlm.nih.gov/pubmed/25494599 |
| Lorenc A, Feder G, MacPherson H, Little P, Mercer SW, Sharp D. Scoping review of systematic reviews of complementary medicine for musculoskeletal and mental health conditions. BMJ open. 2018 Oct 1;8(10). https://bmjopen.bmj.com/content/8/10/e020222?cpetoc= |
| Lorimer K, Kidd L, Lawrence M, McPherson K, Cayless S, Cornish F. Systematic review of reviews of behavioural HIV prevention interventions among men who have sex with men. AIDS care. 2013 Feb 1;25(2):133-50. https://www.tandfonline.com/doi/abs/10.1080/09540121.2012.699672 |
| Lou S, Carstensen K, Jørgensen CR, Nielsen CP. Stroke patients’ and informal carers’ experiences with life after stroke: an overview of qualitative systematic reviews. Disability and rehabilitation. 2017 Jan 30;39(3):301-13. https://www.tandfonline.com/doi/full/10.3109/09638288.2016.1140836?casa_token=2w5usamjo3MAAAAA:Gt-btSjAqw_ast3aA8wM2uAyR2bj4ePSWof2MT97G4BkPlzyPownaqTP8USI27rDNR3Q7nSeFyo |
| Lozano-Montoya I, Correa-Pérez A, Abraha I, Soiza RL, Cherubini A, O’Mahony D, Cruz-Jentoft AJ. Nonpharmacological interventions to treat physical frailty and sarcopenia in older patients: a systematic overview–the SENATOR Project ONTOP Series. Clinical interventions in aging. 2017;12:721. https://www.ncbi.nlm.nih.gov/pmc/articles/PMC5413484/ |
| Luo J, Shang Q, Han M, Chen K, Xu H. Traditional Chinese medicine injection for angina pectoris: an overview of systematic reviews. The American journal of Chinese medicine. 2014;42(01):37-59. https://www.worldscientific.com/doi/abs/10.1142/S0192415X14500037 |
| Luo J, Song W, Yang G, Xu H, Chen K. Compound Danshen (Salvia miltiorrhiza) Dripping Pill for Coronary Heart Disease: An Overview of Systematic Reviews. The American Journal of Chinese Medicine. 2015;43(1):25-43. |
| Luo J, Xu H, Yang G, Qiu Y, Liu J, Chen K. Oral Chinese proprietary medicine for angina pectoris: an overview of systematic reviews/meta-analyses. Complementary therapies in medicine. 2014 Aug 1;22(4):787-800. https://www.sciencedirect.com/science/article/abs/pii/S096522991400096X |
| Luo J, Xu H. Outcome measures of Chinese herbal medicine for coronary heart disease: an overview of systematic reviews. Evidence-Based Complementary and Alternative Medicine. 2012;2012. https://www.hindawi.com/journals/ecam/2012/927392/abs/ |
| Lyra, L., What do Cochrane systematic reviews say about interventions for autism spectrum disorders? Sao Paulo Medical Journal. 2017;135(2):192-201. http://www.scielo.br/pdf/spmj/v135n2/1806-9460-spmj-135-02-00192.pdf |
| Mackenzie H, Drahota A, Pallikadavath S, Stones W, Dean T, Fogg C, Stores R, Kilburn S, Dewey A, Ogollah R. What is the impact of contraceptive methods and mixes of contraceptive methods on contraceptive prevalence, unmet need for family planning, and unwanted and unintended pregnancies? EPPI Centre, Social Science Research Unit. Institute of Education, University of London. https://researchportal.port.ac.uk/portal/en/publications/what-is-the-impact-of-contraceptive-methods-and-mixes-of-contraceptive-methods-on-contraceptive-prevalence-unmet-need-for-family-planning-and-unwanted-and-unintended-pregnancies(a880c960-d4c3-45a1-a47e-123890ede484).html |
| Mac-Namara M, Rada G. Is rituximab effective for induction of remission in lupus nephritis?. https://europepmc.org/abstract/med/25334007 |
| Main C, Thomas S, Ogilvie D, Stirk L, Petticrew M, Whitehead M, Sowden A. Population tobacco control interventions and their effects on social inequalities in smoking: placing an equity lens on existing systematic reviews. BMC public health. 2008 Dec;8(1):178. https://bmcpublichealth.biomedcentral.com/articles/10.1186/1471-2458-8-178 |
| Manheimer E, Wieland S, Kimbrough E, Cheng K, Berman BM. Evidence from the Cochrane Collaboration for traditional Chinese medicine therapies. The Journal of Alternative and Complementary Medicine. 2009 Sep 1;15(9):1001-14. https://www.ncbi.nlm.nih.gov/pmc/articles/PMC2856612/ |
| Manzoli L, Ioannidis JP, Flacco ME, De Vito C, Villari P. Effectiveness and harms of seasonal and pandemic influenza vaccines in children, adults and elderly: a critical review and re-analysis of 15 meta-analyses. Hum Vaccin Immunother. 2012 Jul;8(7):851-62. https://www.tandfonline.com/doi/pdf/10.4161/hv.19917 |
| March S, Torres E, Ramos M, Ripoll J, García A, Bulilete O, et al. Adult community health-promoting interventions in primary health care: A systematic review. Preventive Medicine. 2015;76:S94-S104 |
| Marcolino MS, Oliveira JA, D'Agostino M, Ribeiro AL, Alkmim MB, Novillo-Ortiz D. The impact of mHealth interventions: systematic review of systematic reviews. JMIR mHealth and uHealth. 2018;6(1):e23. https://mhealth.jmir.org/2018/1/e23/ |
| Marinho VC. Cochrane reviews of randomized trials of fluoride therapies for preventing dental caries. Eur Arch Paediatr Dent. 2009 Sep;10(3):183-91 |
| Markozannes G, Aretouli E, Rintou E, Dragioti E, Damigos D, Ntzani E, et al. An umbrella review of the literature on the effectiveness of psychological interventions for pain reduction. BMC psychology. 2017;5(1):31-16. https://bmcpsychology.biomedcentral.com/articles/10.1186/s40359-017-0200-5 |
| Marrone M, Stewart A, Dotson WD. Clinical utility of gene-expression profiling in women with early breast cancer: an overview of systematic reviews. Genetics in medicine : official journal of the American College of Medical Genetics. 2015;17(7):519-32. |
| Martel G, Duhaime S, Barkun JS, Boushey RP, Ramsay CR, Fergusson DA. The quality of research synthesis in surgery: the case of laparoscopic surgery for colorectal cancer. Systematic reviews. 2012 Dec;1(1):14.https://systematicreviewsjournal.biomedcentral.com/articles/10.1186/2046-4053-1-14 |
| Martin EK, Beckmann MM, Barnsbee LN, Halton KA, Merollini KM, Graves N. Best practice perioperative strategies and surgical techniques for preventing caesarean section surgical site infections: a systematic review of reviews and meta‐analyses. BJOG: An International Journal of Obstetrics & Gynaecology. 2018 Jul 1;125(8):956-64.https://www.ncbi.nlm.nih.gov/pubmed/29336106 |
| Martin Ginis KA, Ma JK, Latimer-Cheung AE, Rimmer JH. A systematic review of review articles addressing factors related to physical activity participation among children and adults with physical disabilities. Health psychology review. 2016 Oct 1;10(4):478-94. https://www.tandfonline.com/doi/abs/10.1080/17437199.2016.1198240 |
| Martineau F, Tyner E, Lorenc T, Petticrew M, Lock K. Population-level interventions to reduce alcohol-related harm: an overview of systematic reviews. Preventive medicine. 2013 Oct 1;57(4):278-96. https://www.sciencedirect.com/science/article/pii/S0091743513002119 |
| Martins DE, Astur N, Kanas M, Ferretti M, Lenza M, Wajchenberg M. Quality assessment of systematic reviews for surgical treatment of low back pain: an overview. The Spine Journal. 2016 May 1;16(5):667-75. https://www.sciencedirect.com/science/article/abs/pii/S1529943016002989 |
| Martis R, Crowther CA, Shepherd E, Alsweiler J, Downie MR, Brown J. Treatments for women with gestational diabetes mellitus: an overview of Cochrane systematic reviews. Cochrane Database Syst Rev. 2018 Aug 14;8:CD012327. https://www.ncbi.nlm.nih.gov/pmc/articles/PMC6513179/ |
| Mascarenhas R, Cvetanovich GL, Sayegh ET, Verma NN, Cole BJ, Bush-Joseph C, Bach Jr BR. Does double-bundle anterior cruciate ligament reconstruction improve postoperative knee stability compared with single-bundle techniques? A systematic review of overlapping meta-analyses. Arthroscopy: The Journal of Arthroscopic & Related Surgery. 2015 Jun 1;31(6):1185-96. https://www.sciencedirect.com/science/article/pii/S0749806314009074 |
| Mascarenhas, Randy, M.D., F.R.C.S.C., Erickson BJ, M.D., Sayegh ET, B.S., Verma NN, M.D., Cole, Brian J., M.D., M.B.A., Bush-Joseph C, M.D., et al. Is There a Higher Failure Rate of Allografts Compared With Autografts in Anterior Cruciate Ligament Reconstruction: A Systematic Review of Overlapping Meta-analyses. Arthroscopy: The Journal of Arthroscopic and Related Surgery. 2015;31(2):364-72 |
| Mateussi MV, Latorraca CD, Daou JP, Martimbianco AL, Riera R, Pacheco RL, Pachito DV. What do Cochrane systematic reviews say about interventions for vitamin D supplementation?. Sao Paulo Medical Journal. 2017 Oct;135(5):497-507. |
| Matsui Y, Satoi S, Hirooka S, Kosaka H, Kawaura T, Kitawaki T. Reappraisal of previously reported meta-analyses on antibiotic prophylaxis for low-risk laparoscopic cholecystectomy: an overview of systematic reviews. BMJ open. 2018 Mar 1;8(3):e016666. https://bmjopen.bmj.com/content/8/3/e016666?int_source=trendmd&int_medium=cpc&int_campaign=usage-042019 |
| Matwiejczyk L, Mehta K, Scott J, Tonkin E, Coveney J. Characteristics of Effective Interventions Promoting Healthy Eating for Pre-Schoolers in Childcare Settings: An Umbrella Review. Nutrients. 2018 Mar 1;10(3):293. https://www.ncbi.nlm.nih.gov/pmc/articles/PMC5872711/ |
| Mavedzenge SN, Luecke E, Ross DA. Effective approaches for programming to reduce adolescent vulnerability to HIV infection, HIV risk, and HIV-related morbidity and mortality: a systematic review of systematic reviews. JAIDS Journal of Acquired Immune Deficiency Syndromes. 2014 Jul 1;66:S154-69. |
| Mayo-Wilson E, Ng SM, Chuck RS, Li T. The quality of systematic reviews about interventions for refractive error can be improved: a review of systematic reviews. BMC ophthalmology. 2017 Dec;17(1):164. **https://bmcophthalmol.biomedcentral.com/articles/10.1186/s12886-017-0561-9** |
| Mazuquin BF, Wright AC, Russell S, Monga P, Selfe J, Richards J. Effectiveness of early compared with conservative rehabilitation for patients having rotator cuff repair surgery: an overview of systematic reviews. Br J Sports Med. 2018 Jan 1;52(2):111-21. https://bjsm.bmj.com/content/bjsports/52/2/111.full.pdf |
| Mbemba G, Gagnon M, Paré G, Côté J. Interventions for supporting nurse retention in rural and remote areas: an umbrella review. Human resources for health. 2013;11(1):44- |
| Mbuagbaw L, Mursleen S, Lytvyn L, Smieja M, Dolovich L, Thabane L. Mobile phone text messaging interventions for HIV and other chronic diseases: an overview of systematic reviews and framework for evidence transfer. BMC health services research. 2015 Dec;15(1):33. https://bmchealthservres.biomedcentral.com/articles/10.1186/s12913-014-0654-6 |
| McBain H, Shipley M, Newman S. The impact of self-monitoring in chronic illness on healthcare utilisation: a systematic review of reviews. BMC health services research. 2015;15(1):565 |
| McDermott O, Charlesworth G, Hogervorst E, Stoner C, Moniz-Cook E, Spector A, Csipke E, Orrell M. Psychosocial interventions for people with dementia: a synthesis of systematic reviews. Aging & mental health. 2018 Jan 3;23(4):393-403. https://www.tandfonline.com/doi/full/10.1080/13607863.2017.1423031 |
| McNeill J, Lynn F, Alderdice F. Public health interventions in midwifery: a systematic review of systematic reviews. BMC Public Health. 2012 Dec;12(1):955. |
| McRae MP. Dietary fiber intake and type 2 diabetes mellitus: an umbrella review of meta-analyses. Journal of chiropractic medicine. 2018 Mar 1;17(1):44-53. |
| McRae MP. Health benefits of dietary whole grains: an umbrella review of meta-analyses. Journal of chiropractic medicine. 2017 Mar 1;16(1):10-8. https://www.sciencedirect.com/science/article/abs/pii/S1556370716300736 |
| McRae MP. Therapeutic benefits of l-arginine: an umbrella review of meta-analyses. Journal of chiropractic medicine. 2016 Sep 1;15(3):184-9. |
| Mead A, Atkinson G, Albin D, Alphey D, Baic S, Boyd O, Cadigan L, Clutton L, Craig L, Flanagan C, Greene P, Griffiths E, Lee NJ, Li M, McKechnie L, Ottaway J, Paterson K, Perrin L, Rigby P, Stone D, Vine R, Whitehead J, Wray L, Hooper L; UK Heart Health Group; Thoracic Dietitians Interest Group (Specialist group of the British Dietetic Association). Dietetic guidelines on food and nutrition in the secondary prevention of cardiovascular disease - evidence from systematic reviews of randomized controlled trials (second update, January 2006) https://onlinelibrary.wiley.com/doi/pdf/10.1111/j.1365-277X.2006.00726.x?casa_token=TlJO9ubfI9AAAAAA:bY1Am6XZH_mlonoiLn7x52y0EQDr5OlTaW7hm5kLVo06M9B9_2vvz2iEUy17vxoftDpSNEJg93EcuDT4 |
| Medley N, Vogel JP, Care A, Alfirevic Z. Interventions during pregnancy to prevent preterm birth: an overview of Cochrane systematic reviews. Cochrane Database Syst Rev. 2018 Nov 14;11:CD012505. |
| Meinshausen M, Rieckert A, Renom-Guiteras A, Kröger M, Sommerauer C, Kunnamo I, Martinez YV, Esmail A, Sönnichsen A. Effectiveness and patient safety of platelet aggregation inhibitors in the prevention of cardiovascular disease and ischemic stroke in older adults - a systematic review. BMC Geriatr. 2017 Oct 16;17(Suppl 1):225. https://bmcgeriatr.biomedcentral.com/articles/10.1186/s12877-017-0572-7 |
| Melchiors AC, Correr CJ, Venson R, Pontarolo R. An analysis of quality of systematic reviews on pharmacist health interventions. Int J Clin Pharm. 2012 Feb;34(1):32-42. |
| Melnik T, Soares BG, Puga ME, Atallah ÁN. Efficacy and safety of atypical antipsychotic drugs (quetiapine, risperidone, aripiprazole and paliperidone) compared with placebo or typical antipsychotic drugs for treating refractory schizophrenia: overview of systematic reviews. Sao Paulo Medical Journal. 2010 May;128(3):141-66. http://dx.doi.org/10.1590/S1516-31802010000300007 |
| Melzer J, Saller R. Clinical studies in peripheral arterial occlusive disease: update from the aspects of a meta-narrative review. Forschende Komplementarmedizin (2006), 13 Jun 2013, 20 Suppl 2:17-21. https://europepmc.org/abstract/med/23860108 |
| Mewton L, Visontay R, Chapman C, Newton N, Slade T, Kay‐Lambkin F, Teesson M. Universal prevention of alcohol and drug use: An overview of reviews in an Australian context. Drug and alcohol review. 2018 Apr;37:S435-69. |
| Meyer S, Gortner L, Larsen A, Kutschke G, Gottschling S, Gräber S, Schroeder N. Complementary and alternative medicine in paediatrics: a systematic overview/synthesis of Cochrane Collaboration reviews. Swiss medical weekly. 2013 May 27;143(2122). |
| Michiels B, Van Puyenbroeck K, Verhoeven V, Vermeire E, Coenen S. The value of neuraminidase inhibitors for the prevention and treatment of seasonal influenza: a systematic review of systematic reviews. PloS one. 2013 Apr 2;8(4):e60348. https://journals.plos.org/plosone/article?id=10.1371/journal.pone.0060348 |
| Mickenautsch S, Yengopal V. Extent and quality of systematic review evidence related to minimum intervention in dentistry: essential oils, powered toothbrushes, triclosan, xylitol. International dental journal. 2011 Aug;61(4):179-92. https://doi.org/10.1111/j.1875-595X.2011.00055.x |
| Mikton C, Butchart A. Child maltreatment prevention: a systematic review of reviews. Bull World Health Organ. 2009 May;87(5):353-61. https://www.scielosp.org/scielo.php?pid=S0042-96862009000500012&script=sci_abstract&tlng=fr |
| Miller M, Wood L. Effectiveness of smoking cessation interventions: review of evidence and implications for best practice in Australian health care settings. Aust N Z J Public Health. 2003;27(3):300-9. https://onlinelibrary.wiley.com/doi/pdf/10.1111/j.1467-842X.2003.tb00399.x |
| Mistiaen P, Francke AL, Poot E. Interventions aimed at reducing problems in adult patients discharged from hospital to home: a systematic meta-review. BMC health services research. 2007 Dec;7(1):47. https://bmchealthservres.biomedcentral.com/articles/10.1186/1472-6963-7-47 |
| Moe RH, Haavardsholm EA, Christie A, Jamtvedt G, Dahm KT, Hagen KB. Effectiveness of nonpharmacological and nonsurgical interventions for hip osteoarthritis: an umbrella review of high-quality systematic reviews. Phys Ther. 2007 Dec;87(12):1716-27.  https://academic.oup.com/ptj/article/87/12/1716/2747276 |
| Moe RH, Kjeken I, Uhlig T, Hagen KB. There is inadequate evidence to determine the effectiveness of nonpharmacological and nonsurgical interventions for hand osteoarthritis: an overview of high-quality systematic reviews. Physical therapy. 2009 Dec 1;89(12):1363-70. https://academic.oup.com/ptj/article/89/12/1363/2737662 |
| Momsen AM, Rasmussen JO, Nielsen CV, Iversen MD, Lund H. Multidisciplinary team care in rehabilitation: an overview of reviews. Journal of rehabilitation medicine. 2012 Nov 5;44(11):901-12. https://www.ingentaconnect.com/contentone/mjl/sreh/2012/00000044/00000011/art00001?crawler=true&mimetype=application/pdf |
| Moore RA, Derry S, Aldington D, Wiffen PJ. Adverse events associated with single dose oral analgesics for acute postoperative pain in adults ‐ an overview of Cochrane reviews. Cochrane Database Syst Rev 2015. 13;(10):CD011407 https://www.ncbi.nlm.nih.gov/pmc/articles/PMC6485338/ |
| Moore RA, Derry S, Aldington D, Wiffen PJ. Single dose oral analgesics for acute postoperative pain in adults‐an overview of Cochrane reviews. Cochrane Database of Systematic Reviews. 2015(9). https://www.cochranelibrary.com/cdsr/doi/10.1002/14651858.CD008659.pub3/abstract |
| Moore RA, Derry S, Wiffen PJ, Straube S, Aldington DJ. Overview review: Comparative efficacy of oral ibuprofen and paracetamol (acetaminophen) across acute and chronic pain conditions. European Journal of Pain. 2015 Oct;19(9):1213-23. https://onlinelibrary.wiley.com/doi/full/10.1002/ejp.649 |
| Moore RA, Wiffen PJ, Derry S, Maguire T, Roy YM, Tyrrell L. Non‐prescription (OTC) oral analgesics for acute pain ‐ an overview of Cochrane reviews. Cochrane Database Syst Rev. 2015 Nov 4;(11):CD010794. https://www.ncbi.nlm.nih.gov/pmc/articles/PMC6485506/ |
| Morche J, Mathes T, Pieper D. Relationship between surgeon volume and outcomes: a systematic review of systematic reviews. Systematic reviews. 2016 Dec;5(1):204. https://systematicreviewsjournal.biomedcentral.com/articles/10.1186/s13643-016-0376-4 |
| Moreira, Maria Stella, DDS, MSc, PhD, Anuar, Anuar Sadat Neres-Santiago, DDS, Tedesco, Tamara Kerber, DDS, MSc, PhD, dos Santos, Marcelo, DDS, MSc, PhD, Morimoto, Susana, DDS, MSc, PhD. Endodontic Treatment in Single and Multiple Visits: An Overview of Systematic Reviews. Journal of Endodontics. 2017;43(6):864-70 |
| Morris RK, Oliver EA, Malin G, Khan KS, Meads C. Effectiveness of interventions for the prevention of small‐for‐gestational age fetuses and perinatal mortality: a review of systematic reviews. Acta obstetricia et gynecologica Scandinavica. 2013 Feb;92(2):143-51. https://obgyn.onlinelibrary.wiley.com/doi/pdf/10.1111/aogs.12029 |
| Morrison DS, Petticrew M, Thomson H. What are the most effective ways of improving population health through transport interventions? Evidence from systematic reviews. Journal of Epidemiology & Community Health. 2003 May 1;57(5):327-33. |
| Mourad S, Brown J, Farquhar C. Interventions for the prevention of OHSS in ART cycles: an overview of Cochrane reviews. Cochrane Database Syst Rev. 2017 Jan 23;1:CD012103. |
| MozeticI, V., DaouII, J.P., MartimbiancoIII, A.L.C., & RieraI, R. What do Cochrane systematic reviews say about diabetic retinopathy? Sao Paulo Medical Journal. 2017;135(1):79-87. http://web.b.ebscohost.com.ezproxy.library.ubc.ca/ehost/pdfviewer/pdfviewer?vid=1&sid=6995af72-b616-45d4-9ee3-ef664a1f44bc%40sessionmgr101 |
| Munoz-Solomando A, Kendall T, Whittington CJ. Cognitive behavioural therapy for children and adolescents. Curr Opin Psychiatr 2008;21:332-337 |
| Murphy LA, Harrington P, Taylor SJ, Teljeur C, Smith SM, Pinnock H, Ryan M. Clinical-effectiveness of self-management interventions in chronic obstructive pulmonary disease: An overview of reviews. Chron Respir Dis. 2017 Aug;14(3):276-288 |
| Nardelli AA, Stafinski T, Motan T, Klein K, Menon D. Assisted reproductive technologies (ARTs): evaluation of evidence to support public policy development. Reproductive health. 2014 Dec;11(1):76. |
| Nasser M, Binsbergen JV, Javaheri H, Yassiny K. Diet and nutrition advice from the Cochrane Library: is it useful for the consumers and family physicians? Family Practice 2008;25(Supplement 1):i60-i66 https://academic.oup.com/fampra/article/25/suppl_1/i60/544044 |
| Negrini S, Imperio G, Villafane JH, Negrini F, Zaina F. Systematic reviews of physical and rehabilitation medicine Cochrane contents. Part 1. Disabilities due to spinal disorders and pain syndromes in adults. Eur J Phys Rehabil Med. 2013 Aug 1;49(4):597-609. https://pdfs.semanticscholar.org/973a/c5f6557d04c572886bfbb3d78a69d6bafd9b.pdf |
| Ng L, Khan F, Young CA, Galea M. Symptomatic treatments for amyotrophic lateral sclerosis/motor neuron disease. Cochrane Database Syst Rev. 2017 Jan 10;1:CD011776 https://www.ncbi.nlm.nih.gov/pmc/articles/PMC6469543/ |
| Ng SS, Lai NM, Nathisuwan S, Chaiyakunapruk N. Interventions and Strategies to Improve Oral Anticoagulant Use in Patients with Atrial Fibrillation: A Systematic Review of Systematic Reviews. Clinical drug investigation. 2018 Jul 1;38(7):579-91. sci-hub.tw/10.1007/s40261-018-0641-5 |
| Nielsen S, Germanos R, Weier M, Pollard J, Degenhardt L, Hall W, Buckley N, Farrell M. The use of cannabis and cannabinoids in treating symptoms of multiple sclerosis: a systematic review of reviews. Current neurology and neuroscience reports. 2018 Feb 1;18(2):8. https://link.springer.com/article/10.1007/s11910-018-0814-x |
| Niet GJD, Tiemens BG, Kloos MW, Hutschemaekers GJM. Review of systematic reviews about the efficacy of non-pharmacological interventions to improve sleep quality in insomnia. International Journal of Evidence-Based Healthcare. 2009;7(4):233-42 |
| Novak I, Morgan C, Adde L, Blackman J, Boyd RN, Brunstrom-Hernandez J, Cioni G, Damiano D, Darrah J, Eliasson AC, de Vries LS, Einspieler C, Fahey M, Fehlings D, Ferriero DM, Fetters L, Fiori S, Forssberg H, Gordon AM, Greaves S, Guzzetta A, Hadders-Algra M, Harbourne R, Kakooza-Mwesige A, Karlsson P, Krumlinde-Sundholm L, Latal B, Loughran-Fowlds A, Maitre N, McIntyre S, Noritz G, Pennington L, Romeo DM, Shepherd R, Spittle AJ, Thornton M, Valentine J, Walker K, White R, Badawi N. Early, Accurate Diagnosis and Early Intervention in Cerebral Palsy: Advances in Diagnosis and Treatment. JAMA Pediatr. 2017 Sep 1;171(9):897-907. https://www.zora.uzh.ch/id/eprint/149328/ |
| Nurmatov U, Dhami S, Arasi S, Roberts G, Pfaar O, Muraro A, Ansotegui IJ, Calderon M, Cingi C, Durham S, Van Wijk RG. Allergen immunotherapy for allergic rhinoconjunctivitis: a systematic overview of systematic reviews. Clinical and translational allergy. 2017 Dec;7(1):24. https://ctajournal.biomedcentral.com/articles/10.1186/s13601-017-0159-6 |
| O’Malley, N., Blauth, M., Suhm, N. & Kates, S. Hip fracture management, before and beyond surgery and medication: a synthesis of the evidence. Archives of Orthopaedic and Trauma Surgery. 2011;131(11):1519-1527. https://link-springer-com.ezproxy.library.ubc.ca/content/pdf/10.1007/s00402-011-1341-2.pdf |
| Oaklander AL, Lunn MP, Hughes RA, van Schaik IN, Frost C, Chalk. Treatments for chronic inflammatory demyelinating polyradiculoneuropathy (CIDP): an overview of systematic reviews. Cochrane Database Syst Rev. 2017 Jan 13;1:CD010369. https://www.ncbi.nlm.nih.gov/pmc/articles/PMC5468847/ |
| O'Connell NE, Wand BM, McAuley J, Marston L, Moseley GL. Interventions for treating pain and disability in adults with complex regional pain syndrome‐an overview of systematic reviews. Cochrane Database of Systematic Reviews. 2013(4). https://www.cochranelibrary.com/cdsr/doi/10.1002/14651858.CD009416.pub2/full |
| O'Donnell A, Anderson P, Newbury-Birch D, Schulte B, Schmidt C, Reimer J, et al. The impact of brief alcohol interventions in primary healthcare: a systematic review of reviews. Alcohol and alcoholism (Oxford, Oxfordshire). 2014;49(1):66-78. |
| Oestergaard S, Møldrup C. Improving outcomes for patients with depression by enhancing antidepressant therapy with non-pharmacological interventions: a systematic review of reviews. Public Health. 2011 Jun 1;125(6):357-67. https://doi.org/10.1016/j.puhe.2011.02.001 |
| Olaithe M, Bucks RS, Hillman DR, Eastwood PR. Cognitive deficits in obstructive sleep apnea: Insights from a meta-review and comparison with deficits observed in COPD, insomnia, and sleep deprivation. Sleep Med Rev. 2018 Apr;38:39-49. http://tinyurl.com/y6jxw7dc |
| Oliveira RD, Mazzucca AC, Pachito DV, Riera R, Baptista-Silva JC. Evidence for varicose vein treatment: an overview of systematic reviews. Sao Paulo Medical Journal. 2018 Aug;136(4):324-32. http://dx.doi.org/10.1590/1516-3180.2018.0003240418 |
| Onakpoya IJ, Walker AS, Tan PS, Spencer EA, Gbinigie OA, Cook J, Llewelyn MJ, Butler CC. Overview of systematic reviews assessing the evidence for shorter versus longer duration antibiotic treatment for bacterial infections in secondary care. PloS one. 2018 Mar 28;13(3):e0194858. |
| Onakpoya IJ, Wider B, Pittler MH, Ernst E. Food supplements for body weight reduction: a systematic review of systematic reviews. Obesity. 2011 Feb 1;19(2):239. https://doi.org/10.1038/oby.2010.185 |
| Onasanya O, MBChB, Iyer G, MBBS, Lucas E, BA, Lin D, MHS, Singh S, MD, Alexander GC, Dr. Association between exogenous testosterone and cardiovascular events: an overview of systematic reviews. Lancet Diabetes & Endocrinology, The. 2016;4(11):943-56. |
| Ooi SL, Giovino M, Pak SC. Transcendental meditation for lowering blood pressure: An overview of systematic reviews and meta-analyses. Complementary Therapies in Medicine. 2017;34:26-34. https://www.sciencedirect.com/science/article/abs/pii/S0965229917302285https://www.researchgate.net/profile/Soo_Liang_Ooi/publication/318655835_Transcendental_meditation_for_lowering_blood_pressure_An_overview_of_systematic_reviews_and_meta-analyses/links/597d824fa6fdcc1a9aca4873/Transcendental-meditation-for-lowering-blood-pressure-An-overview-of-systematic-reviews-and-meta-analyses.pdf |
| Ouwens M, Wollersheim H, Hermens R, Hulscher M, Grol R. Integrated care programmes for chronically ill patients: a review of systematic reviews. International journal for quality in health care. 2005 Jan 21;17(2):141-6. https://academic.oup.com/intqhc/article/17/2/141/1794384 |
| Pacheco RL, Hosni ND, Latorraca CD, Martimbianco AL, Pachito DV, Yarak S, Riera R. What do Cochrane systematic reviews say about interventions for treating psoriasis?. Sao Paulo Medical Journal. 2018 Aug;136(4):354-60. http://www.scielo.br/scielo.php?pid=S1516-31802018000400354&script=sci_arttext |
| Pacheco RL, Trevizo J, Souza CA, Alves G, Sakaya B, Thiago L, Góis AF, Riera R. What do Cochrane systematic reviews say about cardiac arrest management?. Sao Paulo Medical Journal. 2018 Mar;136(2):170-6.http://www.scielo.br/scielo.php?pid=S1516-31802018000200170&script=sci_arttext |
| Pantoja T, Opiyo N, Lewin S, Paulsen E, Ciapponi A, Wiysonge CS, Herrera CA, Rada G, Peñaloza B, Dudley L, Gagnon MP, Garcia Marti S, Oxman AD. Implementation strategies for health systems in low‐income countries: an overview of systematic reviews. Cochrane Database Syst Rev. 2017 Sep 12;9:CD011086 |
| Papageorgiou PN, Deschner J, Papageorgiou SN. Effectiveness and Adverse Effects of Deep Brain Stimulation: Umbrella Review of Meta-Analyses. Journal of Neurological Surgery. Part A: Central European Neurosurgery, 78(02):180-190.  https://www.zora.uzh.ch/id/eprint/133150/1/p14_OK.pdf |
| Parisod H, Pakarinen A, Kauhanen L, Aromaa M, Leppänen V, Liukkonen TN, Smed J, Salanterä S. Promoting children's health with digital games: A review of reviews. Games for Health: Research, Development, and Clinical Applications. 2014 Jun 1;3(3):145-56. https://www.liebertpub.com/doi/full/10.1089/g4h.2013.0086 |
| Parke HL, Epiphaniou E, Pearce G, Taylor SJ, Sheikh A, Griffiths CJ, Greenhalgh T, Pinnock H. Self-management support interventions for stroke survivors: a systematic meta-review. PloS one. 2015;10(7). https://www.ncbi.nlm.nih.gov/pmc/articles/PMC4512724/ |
| Parker EA, Roy T, D'Adamo CR, Wieland LS. Probiotics and gastrointestinal conditions: An overview of evidence from the Cochrane Collaboration. Nutrition. 2018 Jan 1;45:125-34. |
| Parkes J, Bryant J, Milne R. Implantable cardioverter defibrillators: arrhythmias. A rapid and systematic review. Health Technol Assess. 2000;4(26):1-69. |
| Pasanen T, Tolvanen S, Heinonen A, Kujala UM. Exercise therapy for functional capacity in chronic diseases: an overview of meta-analyses of randomised controlled trials. Br J Sports Med. 2017 Oct 1;51(20):1459-65. http://dx.doi.org/10.1136/bjsports-2016-097132 |
| Patnode CD, Henderson JT, Thompson JH, NCBI Bookshelf. Behavioral Counseling and Pharmacotherapy Interventions for Tobacco Cessation in Adults, Including Pregnant Women: A Review of Reviews for the U.S. Preventive Services Task Force. Agency for Healthcare Research and Quality (US); 2015. https://annals.org/aim/fullarticle/2443059 |
| Payne C, Wiffen PJ, Martin S. Interventions for fatigue and weight loss in adults with advanced progressive illness. Cochrane Database Syst Rev. 2017 Apr 7;4(4):CD008427. https://www.ncbi.nlm.nih.gov/pmc/articles/PMC6478103/ |
| Pearce G, Pinnock H, Epiphaniou E, Parke HL, Heavey E, Griffiths CJ, Greenhalgh T, Sheikh A, Taylor SJ. Experiences of self-management support following a stroke: a meta-review of qualitative systematic reviews. PloS one. 2015 Dec 14;10(12):e0141803. https://journals.plos.org/plosone/article?id=10.1371/journal.pone.0141803 |
| Pelsser LM, Frankena K, Toorman J, Rodrigues Pereira R. Diet and ADHD, Reviewing the Evidence: A Systematic Review of Meta-Analyses of Double-Blind Placebo-Controlled Trials Evaluating the Efficacy of Diet Interventions on the Behavior of Children with ADHD. PLoS One. 2017 Jan 25;12(1):e0169277.  https://www.ncbi.nlm.nih.gov/pmc/articles/PMC5266211/ |
| Penate W, Fumero A. A meta-review of Internet computer-based psychological treatments for anxiety disorders. Journal of telemedicine and telecare. 2016 Jan;22(1):3-11. https://journals.sagepub.com/doi/abs/10.1177/1357633X15586491 |
| **Perry R, Leach V, Davies P, Penfold C, Ness A, Churchill R. An overview of systematic reviews of complementary and alternative therapies for fibromyalgia using both AMSTAR and ROBIS as quality assessment tools. Syst Rev. 2017 May 15;6(1):97.** |
| Peters LW, Kok G, Ten Dam GT, Buijs GJ, Paulussen TG. Effective elements of school health promotion across behavioral domains: a systematic review of reviews. BMC Public Health. 2009 Jun 12;9:182.  https://www.ncbi.nlm.nih.gov/pmc/articles/PMC2702385/ |
| Phillips-Reed LD, Austin PN, Rodriguez RE. Pediatric tonsillectomy and ketorolac. Journal of PeriAnesthesia Nursing. 2016 Dec 1;31(6):485-94. https://www.sciencedirect.com/science/article/abs/pii/S1089947216000356 |
| Piane GM. Evidence-based practices to reduce maternal mortality: a systematic review. Journal of Public Health. 2008 Sep 9;31(1):26-31. https://doi.org/10.1093/pubmed/fdn074 |
| Pieper D, Mathes T, Neugebauer E, Eikermann M. State of evidence on the relationship between high-volume hospitals and outcomes in surgery: a systematic review of systematic reviews. Journal of the American College of Surgeons. 2013 May 1;216(5):1015-25. https://www.journalacs.org/article/S1072-7515(13)00046-X/abstract |
| Pierron A, Fond-Harmant L, Laurent A, Alla F. Supporting parenting to address social inequalities in health: a synthesis of systematic reviews. BMC public health. 2018 Dec;18(1):1087. https://bmcpublichealth.biomedcentral.com/articles/10.1186/s12889-018-5915-6 |
| Pilkington K, Boshnakova A. Complementary medicine and safety: a systematic investigation of design and reporting of systematic reviews. Complement Ther Med. 2012 Feb-Apr;20(1-2):73-82. http://tinyurl.com/y58wy2ho |
| Pinnock H, Parke HL, Panagioti M, Daines L, Pearce G, Epiphaniou E, Bower P, Sheikh A, Griffiths CJ, Taylor SJ. Systematic meta-review of supported self-management for asthma: a healthcare perspective. BMC medicine. 2017 Dec;15(1):64. https://bmcmedicine.biomedcentral.com/articles/10.1186/s12916-017-0823-7 |
| Pittler MH, Ernst E. Dietary supplements for body-weight reduction: a systematic review. The American journal of clinical nutrition. 2004;79(4):529-36. https://academic.oup.com/ajcn/article/79/4/529/4690126 |
| Płaszewski M, Bettany-Saltikov J. Non-surgical interventions for adolescents with idiopathic scoliosis: an overview of systematic reviews. PloS one. 2014 Oct 29;9(10):e110254. https://journals.plos.org/plosone/article?id=10.1371/journal.pone.0110254 |
| Poder, T. G., Fisette, J.-F., & Déry, V. (2018). Speech Recognition for Medical Dictation: Overview in Quebec and Systematic Review. Journal of Medical Systems, 42(5).  sci-hub.tw/10.1007/s10916-018-0947-0 |
| Pollock A, Farmer SE, Brady MC, Langhorne P, Mead GE, Mehrholz J, van Wijck F. Interventions for improving upper limb function after stroke. Cochrane Database Syst Rev. 2014 Nov 12;(11):CD010820. https://www.ncbi.nlm.nih.gov/pmc/articles/PMC6469541/ |
| Pollock M, Sinha IP, Hartling L, Rowe BH, Schreiber S, Fernandes RM. Inhaled short‐acting bronchodilators for managing emergency childhood asthma: an overview of reviews. Allergy. 2017 Feb;72(2):183-200. https://onlinelibrary.wiley.com/doi/pdf/10.1111/all.13039?casa_token=87EiIFdHk8cAAAAA:UZj92wxocIbDPRHZZpIsLIc4a08d7AFPzxPvLYiFWaqtDK_pEgGbFxSbEF0o6MiwdwatGZAqE29H |
| Poole R, Kennedy OJ, Roderick P, Fallowfield JA, Hayes PC, Parkes J. Coffee consumption and health: umbrella review of meta-analyses of multiple health outcomes. BMJ 2017;359:j5024  https://www.bmj.com/content/359/bmj.j5024 |
| Poolman RW, Abouali JA, Conter HJ, Bhandari M. Overlapping systematic reviews of anterior cruciate ligament reconstruction comparing hamstring autograft with bone-patellar tendon-bone autograft: why are they different?. JBJS. 2007 Jul 1;89(7):1542-52. https://journals.lww.com/jbjsjournal/Fulltext/2007/07000/Overlapping_Systematic_Reviews_of_Anterior.19.aspx?casa_token=TpnDDiHwGxsAAAAA:jgk6Czs31yrZPEB5XB4PSfJmuDNqHv5z6ijwhzA_h8F9ddOs5uysmoezDwAtH1Cdm50mh6dqvi8YeGApWQPp5Q |
| Posadzki P, AlBedah AM, Khalil MM, AlQaed MS, Lee MS, Ernst E, et al. Complementary and alternative medicine for the prevention and treatment of migraine headache: an overview of systematic reviews. Focus on Alternative and Complementary Therapies. 2015;20(2):58-73 |
| Posadzki P, Ernst E. Spinal manipulation: an update of a systematic review of systematic reviews. NZ Med J. 2011 Aug 12;124(1340):55-71. https://www.nzma.org.nz/journal/read-the-journal/all-issues/2010-2019/2011/vol-124-no-1340/article-posadzki |
| Posadzki P, Ernst E. Systematic reviews of spinal manipulations for headaches: an attempt to clear up the confusion. Headache: The Journal of Head and Face Pain. 2011 Oct;51(9):1419-25. https://headachejournal.onlinelibrary.wiley.com/doi/epdf/10.1111/j.1526-4610.2011.01888.x |
| Posadzki P, Lee MS, Ernst E. Complementary and alternative medicine for diabetes mellitus: an overview of systematic reviews: Review. Focus on Alternative and Complementary Therapies. 2012;17(3):142-8. https://onlinelibrary.wiley.com/doi/abs/10.1111/j.2042-7166.2012.01159.x |
| Posadzki P, PhD, AlBedah, Abdullah M.N., MBBS, FFCM (KSU), Khalil, Mohamed M.K., MBBCh, MPH, MSc, MD, AlQaed MS, MBBS. Complementary and alternative medicine for lowering blood lipid levels: a systematic review of systematic reviews. Complementary Therapies in Medicine. 2016;29:141-51. |
| Posadzki P, Watson L, Ernst E. Contamination and adulteration of herbal medicinal products (HMPs): an overview of systematic reviews. European Journal of Clinical Pharmacology. 2013;69(3):295-307. |
| Posadzki P, Watson L, Ernst E. Herb-drug interactions: an overview of systematic reviews: Herb−drug interactions. British Journal of Clinical Pharmacology. 2013;75(3):603-18. |
| Posadzki P. Is spinal manipulation effective for pain? An overview of systematic reviews. Pain Medicine. 2012 Jun 1;13(6):754-61. https://academic.oup.com/painmedicine/article/13/6/754/1839832 |
| Posadzki PP, Bajpai R, Kyaw BM, Roberts NJ, Brzezinski A, Christopoulos GI, Divakar U, Bajpai S, Soljak M, Dunleavy G, Jarbrink K. Melatonin and health: an umbrella review of health outcomes and biological mechanisms of action. BMC medicine. 2018 Dec;16(1):18. https://bmcmedicine.biomedcentral.com/articles/10.1186/s12916-017-1000-8 |
| Pöttgen S, Samkange-Zeeb F, Brand T, Steenbock B, Pischke CR. Effectiveness of School-based Interventions to Prevent and/or Reduce Substance Use among Primary and Secondary School Pupils: A Review of Reviews. Gesundheitswesen (Bundesverband der Arzte des Offentlichen Gesundheitsdienstes (Germany)), 24 Mar 2015, 78(4):230-236. DOI: 10.1055/s-0035-1547275 PMID: 25806506. https://europepmc.org/abstract/med/25806506 |
| Prasad K, Karlupia N, Kumar A. Treatment of bacterial meningitis: An overview of Cochrane systematic reviews. Respiratory Medicine. 2009;103(7):945-50. https://www.sciencedirect.com/science/article/pii/S0954611109001036 |
| Prothero L, Barley E, Galloway J, Georgopoulou S, Sturt J. The evidence base for psychological interventions for rheumatoid arthritis: A systematic review of reviews. International journal of nursing studies. 2018 Jun 1;82:20-9. https://doi.org/10.1016/j.ijnurstu.2018.03.008 |
| Puggina A, Aleksovska K, Buck C, Burns C, Cardon G, Carlin A, Chantal S, Ciarapica D, Condello G, Coppinger T, Cortis C. Policy determinants of physical activity across the life course: a ‘DEDIPAC’ umbrella systematic literature review. The European Journal of Public Health. 2017 Oct 18;28(1):105-18. https://academic.oup.com/eurpub/article/28/1/105/4557560 |
| Purcell R, McInnes S, Halcomb EJ. Telemonitoring can assist in managing cardiovascular disease in primary care: a systematic review of systematic reviews. BMC family practice. 2014 Dec;15(1):43. https://bmcfampract.biomedcentral.com/articles/10.1186/1471-2296-15-43 |
| Puschner B, Born A, Giessler A, Helm H, Becker T, Angermeyer MC. Effects of interventions to improve compliance with antipsychotic medication in people suffering from schizophrenia-results of recent reviews. Psychiatr Prax. 2005 Mar;32(2):62-7. https://sci-hub.tw/10.1055/s-2004-828332 |
| Raichand S, Dunn AG, Ong MS, Bourgeois FT, Coiera E, Mandl KD. Conclusions in systematic reviews of mammography for breast cancer screening and associations with review design and author characteristics. Syst Rev. 2017; 6: 105 https://www.ncbi.nlm.nih.gov/pmc/articles/PMC5441061/ |
| Ranney L, Melvin C, Lux L, McClain E, Lohr KN. Systematic review: smoking cessation intervention strategies for adults and adults in special populations. Annals of Internal Medicine. 2006 Dec 5;145(11):845-56. https://annals.org/aim/fullarticle/730874 |
| Re, L. G. and Fusetti, V. [Effectiveness of blood pressure home monitoring. Synopsis of systematic reviews.] Prof Inferm. 2017 Jan-Mar;70(1):3-11. doi: 10.7429/pi.2017.701003. http://www.profinf.net/pro3/index.php/IN/article/view/327 |
| Reckley LK, Fernandez-Salvador C, Camacho M. The effect of tonsillectomy on obstructive sleep apnea: an overview of systematic reviews. Nature and science of sleep. 2018;10:105. |
| Recommendations for the management of rheumatoid arthritis in the Eastern Mediterranean region: an adolopment of the 2015 American College of Rheumatology guidelines https://link.springer.com/article/10.1007/s10067-018-4245-5 |
| Reid WD, Yamabayashi C, Goodridge D, Chung F, Hunt MA, Marciniuk DD, Brooks D, Chen YW, Hoens AM, Camp PG. Exercise prescription for hospitalized people with chronic obstructive pulmonary disease and comorbidities: a synthesis of systematic reviews. International journal of chronic obstructive pulmonary disease. 2012;7:297. https://dx.doi.org/10.2147%2FCOPD.S29750 |
| Reif K, de Vries U, Petermann F. What does really help against cancer-related fatigue? An overview of systematic reviews. Pflege. 2012 Dec;25(6):439-57. https://doi.org/10.1024/1012-5302/a000246. |
| Reinhart K, Perner A, Sprung CL, Jaeschke R, Schortgen F, Groeneveld ABJ, et al. Consensus statement of the ESICM task force on colloid volume therapy in critically ill patients. Intensive Care Medicine. 2012;38(3):368-83. |
| Rejnmark L, Bislev LS, Cashman KD, Eiríksdottir G, Gaksch M, Grübler M, Grimnes G, Gudnason V, Lips P, Pilz S, Van Schoor NM. Non-skeletal health effects of vitamin D supplementation: A systematic review on findings from meta-analyses summarizing trial data. PLoS One. 2017 Jul 7;12(7):e0180512. https://journals.plos.org/plosone/article?id=10.1371/journal.pone.0180512 |
| Rensink M, Schuurmans M, Lindeman E, Hafsteinsdottir T. Task‐oriented training in rehabilitation after stroke: systematic review. Journal of advanced nursing. 2009 Apr;65(4):737-54. https://onlinelibrary.wiley.com/doi/pdf/10.1111/j.1365-2648.2008.04925.x |
| Ribeiro EL, de Mendonça Lima T, Vieira ME, Storpirtis S, Aguiar PM. Efficacy and safety of aripiprazole for the treatment of schizophrenia: An overview of systematic reviews. European journal of clinical pharmacology. 2018 Oct 1;74(10):1215-33. https://doi.org/10.1007/s00228-018-2498-1 |
| Riera, R. et al. What do Cochrane systematic reviews say about new practices on integrative medicine? Sao Paulo Medical Journal. 2018;136(3):252-261 http://www.scielo.br/scielo.php?script=sci_arttext&pid=S1516-31802018000300251&lng=en&tlng=en |
| Rimland JM, Abraha I, Dell’Aquila G, Cruz-Jentoft A, Soiza R, Gudmusson A, Petrovic M, O’Mahony D, Todd C, Cherubini A. Effectiveness of non-pharmacological interventions to prevent falls in older people: a systematic overview. The SENATOR Project ONTOP Series. PloS one. 2016 Aug 25;11(8):e0161579. https://journals.plos.org/plosone/article?id=10.1371/journal.pone.0161579 |
| Roberston-Malt, S. (2010). Overview of review: Non-medical management of asthma in adults. International Journal of Nursing Practice, 16(2), 208–219. sci-hub.tw/10.1111/j.1440-172X.2010.01835.x |
| Robinson KA, Saldanha IJ, McKoy NA. Management of infants with cystic fibrosis: a summary of the evidence for the cystic fibrosis foundation working group on care of infants with cystic fibrosis. The Journal of pediatrics. 2009 Dec 1;155(6):S94-105. https://www.sciencedirect.com/science/article/abs/pii/S0022347609008828 |
| Rodgers M, Fayter D, Richardson G, Lewin R, Sowden AJ, Ritchie G. The effects of psychosocial interventions in cancer and heart disease: a review of systematic reviews. York, UK: University of York, 2005. 178 p. (CRD Report). https://pdfs.semanticscholar.org/2db2/d2e5f6f4d0e7d903e99b460811fe127d9a04.pdf |
| Rodriguez-Merchan EC. Evidence-based ACL reconstruction. Archives of Bone and Joint Surgery. 2015 Jan;3(1):9. https://www.ncbi.nlm.nih.gov/pmc/articles/PMC4322130/ |
| Roe B, Flanagan L, Maden M. Systematic review of systematic reviews for the management of urinary incontinence and promotion of continence using conservative behavioural approaches in older people in care homes. Journal of advanced nursing. 2015 Jul;71(7):1464-83. https://onlinelibrary.wiley.com/doi/pdf/10.1111/jan.12613?casa_token=d2KVuViEhjMAAAAA:zc7iQmug_kwx_TSPfQS9Z_IOOL4WRRhLOMcz1UvjX8E2CTGiguW75bqYk3YgMU6EFuBhBu0xMdYyKJI |
| Rosato NS, Correll CU, Pappadopulos E, Chait A, Crystal S, Jensen PS, Treatment of Maladaptive Aggressive in Youth Steering Committee. Treatment of maladaptive aggression in youth: CERT guidelines II. Treatments and ongoing management. Pediatrics. 2012 Jun 1;129(6):e1577-86. https://pediatrics.aappublications.org/content/pediatrics/129/6/e1577.full.pdf?casa_token=3hK27Bifc80AAAAA:EZ_WBKaxjh_aPu97gb27uiul_VM16OzVgMqgMsHkNuLdFh1zp-8mUHV0NgTXCBdCLexoJvRGIg |
| Ruddy R, House A. Meta-review of high-quality systematic reviews of interventions in key areas of liaison psychiatry. The British Journal of Psychiatry. 2005 Aug;187(2):109-20. https://doi.org/10.1192/bjp.187.2.109 |
| Rütten A, Abu-Omar K, Burlacu I, Schätzlein V, Suhrcke M. [Are preventive measures to promote physical activity cost-effective ? A systematic review of overview papers]. Das Gesundheitswesen. 2017 Mar;79(S 01):S51-9. http://doi.org/10.1055/s-0042-123702 |
| Ryan R, Santesso N, Lowe D, Hill S, Grimshaw JM, Prictor M, Kaufman C, Cowie G, Taylor M. Interventions to improve safe and effective medicines use by consumers: an overview of systematic reviews. Cochrane Database of Systematic Reviews. 2014(4). https://doi.org/10.1002/14651858.CD007768.pub3 |
| Safari R, Van der Linden ML, Mercer TH. Effect of exercise interventions on perceived fatigue in people with multiple sclerosis: synthesis of meta-analytic reviews. Neurodegenerative disease management. 2017 Jun;7(3):219-30. https://doi.org/10.2217/nmt-2017-0009 |
| Safron M, Cislak A, Gaspar T, Luszczynska A. Effects of school-based interventions targeting obesity-related behaviors and body weight change: a systematic umbrella review. Behavioral Medicine. 2011 Feb 24;37(1):15-25. https://doi.org/10.1080/08964289.2010.543194 |
| Salleh S, Thokala P, Brennan A, Hughes R, Booth A. Simulation modelling in healthcare: an umbrella review of systematic literature reviews. PharmacoEconomics. 2017 Sep 1;35(9):937-49. https://doi.org/10.1007/s40273-017-0523-3 |
| Sälzer S, Alkilzy M, Slot DE, Dörfer CE, Schmoeckel J, Splieth CH, Chairs of Working Group 3, ORCA. Socio‐behavioural aspects in the prevention and control of dental caries and periodontal diseases at an individual and population level. Journal of clinical periodontology. 2017 Mar;44:S106-15. https://doi.org/10.1111/jcpe.12673 |
| Sälzer S, Slot DE, Van der Weijden FA, Dörfer CE. Efficacy of inter‐dental mechanical plaque control in managing gingivitis–a meta‐review. Journal of clinical periodontology. 2015 Apr;42:S92-105. https://onlinelibrary.wiley.com/doi/pdf/10.1111/jcpe.12363 |
| Sanchez A, Bully P, Martinez C, Grandes G. Effectiveness of physical activity promotion interventions in primary care: A review of reviews. Preventive medicine. 2015 Jul 1;76:S56-67. https://pdf.sciencedirectassets.com/272375/1-s2.0-S0091743515X00089/1-s2.0-S009174351400348X/main.pdf?X-Amz-Security-Token=IQoJb3JpZ2luX2VjEGUaCXVzLWVhc3QtMSJGMEQCIDRHq8IxVgorgqKTyvF1HpetSsZpLebJEZJKJq1B1W02AiBLfz2cWCuof00RSocHTK%2BgTaIhTJBGJHfyva%2FzYmXA%2Fyq9Awj%2B%2F%2F%2F%2F%2F%2F%2F%2F%2F%2F8BEAIaDDA1OTAwMzU0Njg2NSIMXAU3l24fvs0LLnJ0KpEDdBtN92%2Bzb5nLsUQwU9pRd%2FYwQgg3WoNlCD5UogA2Uovm6z6BqW%2FVmHYJ97gqB6zspXPvkdpE1MZyeQKYrHzohRQvpk7rYgkBqUqc5be%2FKcHLH5J%2Fq7%2Fj2nTrFbse3SarTcFh50iS%2BqCToqbnAGlbmzgbqHXDGtqDH1BFJnvX3b01GI3PxWdzav6SP9ZZtlNbAOi2DPmvfUHph1qUT5ga%2FRWZrd2IYcVcYZdK5l0X9GRcUDZcyo%2FtxxrG5a69n3xlFeRwSZnOp3dMGZAqb80FFd%2Bt7KzoHGUt2fzujbZrIEfY8mXOEE72RHRhDlt%2Ffqwbbt6XMBuM0ZBYvKpvfyFbIi2AXqbotfiCM2evtUMGZso6LLaupQpawJuQUZ94vtCrs9prWV13jEizdZUs4sddMWp4CRZfihxxmqDK0ipK8A7lDg%2BYCSrm9c8WS%2F4XovsWumkdzfnfJddg6Ryr3u5uQGuOinPegpPCcOcgReK0DL8FIMrvvIj%2BWIkji743l7aI3ndXYJfKYkjI%2BS6eZMDBiEswj%2BnO8QU67AGTT7J6a2zHj6cZ2%2FTJ5avVI3J2Rl6fR7tgy3AO8QBEvWIIDymeMeJ4h2Z7%2Bbc0xcJIt%2FPuT9TSCB3VP0locpWTWmnEtmHaTJqbWxARG8SivHovjnD5fzdabMkv%2BVXKby7w9WZJqfGsFONILgf0DveLGNGiLIWuWbL%2BhTwUaYRYE0nRPVhTn9BZR1qhWgih2ktqdZ%2B0lOekR%2B9fdYeMGeopX701SZntM13Pl9kLBJjXc7mUY6CZ7fJhgU4IxuMMpO9bzKHMjVEna74AreDDLDWmmy%2B1B9UDjW%2FJijqeFjvPq1W%2F8DQIu2O0vraQpg%3D%3D&X-Amz-Algorithm=AWS4-HMAC-SHA256&X-Amz-Date=20200131T063233Z&X-Amz-SignedHeaders=host&X-Amz-Expires=300&X-Amz-Credential=ASIAQ3PHCVTYT5SN7BHK%2F20200131%2Fus-east-1%2Fs3%2Faws4_request&X-Amz-Signature=1492eb4d4a71edfe2be507c72b6d745121c8b0bf921e91a100cfc024245c7a4c&hash=76283a47cadb32cfc720a8ed97f3f82db695351422997910cfb8ce092b99c220&host=68042c943591013ac2b2430a89b270f6af2c76d8dfd086a07176afe7c76c2c61&pii=S009174351400348X&tid=spdf-f5a4c1ee-a490-4287-8cfe-799c037b6c85&sid=ab1ceb4e2494434c11390e89012b933cfdefgxrqa&type=client |
| Sarkees, M. L., & Bavry, A. A. Acute coronary syndrome (unstable angina and non-ST elevation MI). BMJ clinical evidence, 2009, 0209. https://www.ncbi.nlm.nih.gov/pmc/articles/PMC2907796/pdf/2009-0209.pdf |
| Saunders-Hastings P, Reisman J, Krewski D. Assessing the state of knowledge regarding the effectiveness of interventions to contain pandemic influenza transmission: a systematic review and narrative synthesis. PloS one. 2016;11(12). https://www.ncbi.nlm.nih.gov/pmc/articles/PMC5158032/ |
| Schiltenwolf M, Eidmann U, Köllner V, Kuehn T, Offenbächer M, Petzke F, Sarholz M, Weigl M, Wolf B, Haeuser W. Multimodal therapy of fibromyalgia syndrome: updated guidelines 2017 and overview of systematic review articles. Schmerz (Berlin, Germany). 2017 Jun;31(3):285-8. https://europepmc.org/abstract/med/28493228 |
| Schultz TJ, Roupas P, Wiechula R, Krause D, Gravier S, Tuckett A, Hines S, Kitson A. Nutritional interventions for optimizing healthy body composition in older adults in the community: an umbrella review of systematic reviews. JBI database of systematic reviews and implementation reports. 2016 Aug 1;14(8):257-308. https://doi.org/10.11124/JBISRIR-2016-003063 |
| Schwingshackl L, Hoffmann G, Missbach B, Stelmach-Mardas M, Boeing H. An umbrella review of nuts intake and risk of cardiovascular disease. Current pharmaceutical design. 2017 Feb 1;23(7):1016-27. |
| Schwingshackl L, Hoffmann G. Monounsaturated fatty acids and risk of cardiovascular disease: synopsis of the evidence available from systematic reviews and meta-analyses. Nutrients. 2012;4(12):1989-2007. https://doi.org/10.3390/nu4121989 |
| Secretariat MA. Intra-articular viscosupplementation with hylan gf 20 to treat osteoarthritis of the knee: an evidence-based analysis. Ontario health technology assessment series. 2005;5(10):1. https://www.ncbi.nlm.nih.gov/pmc/articles/PMC3382385/pdf/ohtas-05-66.pdf |
| Segal JB, Streiff MB, Hofmann LV, Thornton K, Bass EB. Management of venous thromboembolism: a systematic review for a practice guideline. Annals of internal medicine. 2007 Feb 6;146(3):211-22. file:///C:/Users/Stephen/Downloads/0000605-200702060-00150%20(1).pdf |
| Seida JK, Ospina MB, Karkhaneh M, Hartling L, Smith V, Clark B. Systematic reviews of psychosocial interventions for autism: an umbrella review. Developmental Medicine & Child Neurology. 2009 Feb;51(2):95-104. https://onlinelibrary.wiley.com/doi/pdf/10.1111/j.1469-8749.2008.03211.x |
| Shamliyan TA, Staal JB, Goldmann D, Sands-Lincoln M. Epidural steroid injections for radicular lumbosacral pain: a systematic review. Physical Medicine and Rehabilitation Clinics. 2014 May 1;25(2):471-89. https://www.academia.edu/download/44175407/Epidural_Steroid_Injections_for_Radicula20160328-14439-r8l510.pdf |
| Shelton TP, Ridgard L, Owens DW. What is the role of sporting or occupational activity in the development of osteoarthritis of the knee or hip? Systematic review of reviews. Pain and Rehabilitation-the Journal of Physiotherapy Pain Association. 2016 Jan 1;2016(40):16-27. https://www.ingentaconnect.com/content/ppa/pr/2016/00002016/00000040/art00005 |
| Shepherd AM, Laurens KR, Matheson SL, Carr VJ, Green MJ. Systematic meta-review and quality assessment of the structural brain alterations in schizophrenia. Neuroscience & Biobehavioral Reviews. 2012 Apr 1;36(4):1342-56. https://www.sciencedirect.com/science/article/pii/S0149763411002223 |
| Shepherd E, Salam RA, Middleton P, Han S, Makrides M, McIntyre S, Badawi N, Crowther CA. Neonatal interventions for preventing cerebral palsy: an overview of Cochrane Systematic Reviews. Cochrane Database of Systematic Reviews. 2018(6). https://doi.org/10.1002/14651858.CD012409.pub2 |
| Shepherd E, Salam RA, Middleton P, Makrides M, McIntyre S, Badawi N, Crowther CA. Antenatal and intrapartum interventions for preventing cerebral palsy: an overview of Cochrane systematic reviews. Cochrane Database of Systematic Reviews. 2017(8). https://www.ncbi.nlm.nih.gov/pmc/articles/PMC6483544/ |
| Shepherd-Banigan M, Goldstein KM, Coeytaux RR, McDuffie JR, Goode AP, Kosinski AS, Van Noord MG, Befus D, Adam S, Masilamani V, Nagi A. Improving vasomotor symptoms; psychological symptoms; and health-related quality of life in peri-or post-menopausal women through yoga: An umbrella systematic review and meta-analysis. Complementary therapies in medicine. 2017 Oct 1;34:156-64. https://doi.org/10.1016/j.ctim.2017.08.011 |
| Sinclair, J. C., et al. (2003). "Cochrane neonatal systematic reviews: a survey of the evidence for neonatal therapies." Clinics in Perinatology 30(2): 285-304. sci-hub.tw/10.1016/s0095-5108(03)00025-3 |
| Singh JA, Christensen R, Wells GA, Suarez‐Almazor ME, Buchbinder R, Lopez‐Olivo MA, Ghogomu ET, Tugwell P. Biologics for rheumatoid arthritis: an overview of Cochrane reviews. Cochrane Database of Systematic Reviews. 2009(4). http://www.scielo.br/pdf/spmj/v128n5/a13v1285.pdf |
| Slev VN, Mistiaen P, Pasman HR, Verdonck-de Leeuw IM, van Uden-Kraan CF, Francke AL. Effects of eHealth for patients and informal caregivers confronted with cancer: a meta-review. International journal of medical informatics. 2016 Mar 1;87:54-67. http://postprint.nivel.nl/PPpp5745.pdf |
| Smidt N, de Vet HC, Bouter LM, Dekker J. Effectiveness of exercise therapy: a best-evidence summary of systematic reviews. Australian Journal of Physiotherapy. 2005 Jan 1;51(2):71-85. https://doi.org/10.1016/S0004-9514(05)70036-2 |
| Smith CA, Carmady B. Acupuncture to treat common reproductive health complaints: An overview of the evidence. Autonomic Neuroscience. 2010 Oct 28;157(1-2):52-6. https://doi.org/10.1016/j.autneu.2010.03.013 |
| Smith KA, Cipriani A. Lithium and suicide in mood disorders: updated meta‐review of the scientific literature. Bipolar disorders. 2017 Nov;19(7):575-86. https://doi.org/10.1111/bdi.12543 |
| **Solloway MR, Taylor SL, Shekelle PG, Miake-Lye IM, Beroes JM, Shanman RM, Hempel S. An evidence map of the effect of Tai Chi on health outcomes. Syst Rev. 2016 Jul 27;5(1):126** |
| Somani N, Turvy D. Hirsutism: an evidence-based treatment update. American journal of clinical dermatology. 2014 Jul 1;15(3):247-66. https://link.springer.com/content/pdf/10.1007/s40257-014-0078-4.pdf |
| Sommer C, Alten R, Bär KJ, Bernateck M, Brückle W, Friedel E, Henningsen P, Petzke F, Tölle T, Üçeyler N, Winkelmann A. Medikamentöse Therapie des Fibromyalgiesyndroms. Der Schmerz. 2017 Jun 1;31(3):274-84. https://doi.org/10.1007/s00482-017-0207-0 |
| Sommerauer C, Schlender L, Krause M, Weissbach S, Rieckert A, Martinez YV, Reeves D, Renom-Guiteras A, Kunnamo I, Soennichsen A. Effectiveness and safety of vitamin K antagonists and new anticoagulants in the prevention of thromboembolism in atrial fibrillation in older adults–a systematic review of reviews and the development of recommendations to reduce inappropriate prescribing. BMC geriatrics. 2017 Oct;17(1):223. https://doi.org/10.1186/s12877-017-0573-6 |
| Song X, Wang S, Hu Y, Yue M, Zhang T, Liu Y, Tian J, Shang K. Impact of ambient temperature on morbidity and mortality: an overview of reviews. Science of the Total Environment. 2017 May 15;586:241-54. https://doi.org/10.1016/j.scitotenv.2017.01.212 |
| Sorgente A, Pietrabissa G, Manzoni GM, Re F, Simpson S, Perona S, Rossi A, Cattivelli R, Innamorati M, Jackson JB, Castelnuovo G. Web-based interventions for weight loss or weight loss maintenance in overweight and obese people: a systematic review of systematic reviews. Journal of medical Internet research. 2017;19(6):e229. https://www.jmir.org/2017/6/e229/pdf |
| Stein DJ, Ipser J, McAnda N. Pharmacotherapy of posttraumatic stress disorder: a review of meta-analyses and treatment guidelines. Cns spectr. 2009 Jan 1;14(1 Suppl 1):25-31. |
| Stephens SK, Cobiac LJ, Veerman JL. Improving diet and physical activity to reduce population prevalence of overweight and obesity: An overview of current evidence. Preventive medicine. 2014 May 1;62:167-78. |
| Steultjens EM, Dekker J, Bouter LM, Leemrijse CJ, van den Ende CH. Evidence of the efficacy of occupational therapy in different conditions: an overview of systematic reviews. Clinical rehabilitation. 2005 May;19(3):247-54. https://doi.org/10.1191%2F0269215505cr870oa |
| Stinson J, Yamada J, Dickson A, Lamba J, Stevens B. Review of systematic reviews on acute procedural pain in children in the hospital setting. Pain Research and Management. 2008;13(1):51-7. http://dx.doi.org/10.1155/2008/465891 |
| Stockley L, Lund V. Use of folic acid supplements, particularly by low-income and young women: a series of systematic reviews to inform public health policy in the UK. Public health nutrition. 2008 Aug;11(8):807-21. https://pdfs.semanticscholar.org/b804/bdb0be52e27b9db57729dfa365d470f60d22.pdf |
| Sutcliffe K, Caird J, Kavanagh J, Rees R, Oliver K, Dickson K, Woodman J, Barnett‐Paige E, Thomas J. Comparing midwife‐led and doctor‐led maternity care: a systematic review of reviews. Journal of advanced nursing. 2012 Nov;68(11):2376-86. https://doi.org/10.1111/j.1365-2648.2012.05998.x |
| Sutcliffe, K., Rees, R., Dickson, K., Hargreaves, K., Schucan-Bird, K., Kwan, I., Kavanagh, J., Woodman, J., Gibson, K. and Thomas, J., 2012. The adult social care outcomes framework: A systematic review of systematic reviews to support its use and development. London: EPPI-Centre, Social Science Research Unit, Institute of Education, University of London. https://pdfs.semanticscholar.org/5c17/82260037011fc27ee1701191f77fd23ce3c6.pdf |
| Suttle CM, Lawrenson JG, Conway ML. Efficacy of coloured overlays and lenses for treating reading difficulty: an overview of systematic reviews. Clinical and Experimental Optometry. 2018 Jul;101(4):514-20. https://onlinelibrary.wiley.com/doi/pdf/10.1111/cxo.12676 |
| Sweeney K, Silver N, Javadpour M. Subarachnoid haemorrhage (spontaneous aneurysmal). BMJ clinical evidence. 2016;2016. https://www.ncbi.nlm.nih.gov/pmc/articles/PMC4794735/pdf/2016-1213.pdf |
| Sweet SN, Fortier MS. Improving physical activity and dietary behaviours with single or multiple health behaviour interventions? A synthesis of meta-analyses and reviews. International journal of environmental research and public health. 2010 Apr;7(4):1720-43. |
| Swinkels A, Cochrane K, Burt A, Johnson L, Lunn T, Rees AS. Exercise interventions for non-specific low back pain: an overview of systematic reviews. Physical Therapy Reviews. 2009 Aug 1;14(4):247-59. https://www.tandfonline.com/doi/pdf/10.1179/174328809X452917 |
| Szajewska H. Probiotics and prebiotics in preterm infants: where are we? Where are we going?. Early human development. 2010 Jul 1;86(1):81-6. |
| Tafelski S, Häuser W, Schäfer M. Efficacy, tolerability, and safety of cannabinoids for chemotherapy-induced nausea and vomiting—a systematic review of systematic reviews. Der Schmerz. 2016 Feb 1;30(1):14-24. https://idp.springer.com/authorize/casa?redirect_uri=https://link.springer.com/article/10.1007/s00482-015-0092-3&casa_token=ALY_TVFvAuYAAAAA:WGtU8rL5RZls3GhcLGNtVcz0x_qzlHIIM5k43h4qPai1OflWAR0q3HdDAeFVmNNnNK7SIrfyxaIC84c |
| Tamayo-Velazquez MI, Simon-Lorda P, Villegas-Portero R, Higueras-Callejón C, García-Gutiérrez JF, Martínez-Pecino F, Barrio-Cantalejo IM. Interventions to promote the use of advance directives: an overview of systematic reviews. Patient education and counseling. 2010 Jul 1;80(1):10-20. https://doi.org/10.1016/j.pec.2009.09.027 |
| Tan SK, Leung WK, Tang AT, Zwahlen RA. How does mandibular advancement with or without maxillary procedures affect pharyngeal airways? An overview of systematic reviews. PloS one. 2017 Jul 27;12(7):e0181146. https://doi.org/10.1371/journal.pone.0181146 |
| Taylor NF, Dodd KJ, Damiano DL. Progressive resistance exercise in physical therapy: a summary of systematic reviews. Physical therapy. 2005 Nov 1;85(11):1208-23. https://doi.org/10.1093/ptj/85.11.1208 |
| Taylor NF, Dodd KJ, Shields N, Bruder A. Therapeutic exercise in physiotherapy practice is beneficial: a summary of systematic reviews 2002–2005. Australian Journal of Physiotherapy. 2007 Jan 1;53(1):7-16. https://pdf.sciencedirectassets.com/280586/1-s2.0-S0004951407X70041/1-s2.0-S0004951407700570/main.pdf?X-Amz-Security-Token=AgoJb3JpZ2luX2VjEHwaCXVzLWVhc3QtMSJIMEYCIQD8hU6HKs5jM5AdE4yljopd%2F6N%2BnS5LqC7MuN%2BQqyapUwIhAPvjpFMSyaR0PXRgbb9hiEiGZY6SmB2kUVExZNUhriGDKtoDCGUQAhoMMDU5MDAzNTQ2ODY1IgyU7lg3NUMBJD8BjcQqtwNyGcAUjrBTUPDrobulDr1eCtwbjEYCoFrpYCX2hw2cGX3s2q7JqmgjPdADzDHU3b26OWFD%2FnaaNKTnQqwoFIrS0yUFJIEowGSzBEx%2BNOChoojinNrl6pusn%2B%2BxPtK6KAYhsZfwH%2BIW3ler4vcIUc%2FbQDzO68zsvUl2zB4%2FjxXrvAYl%2B77VRZ1WJtLo4xEi70cDC1GXHyQ6z7T1QGtD0Gm3LbWtbia0FVEqqfAd%2FmSqd9sYxvVmbOfQ7QbYYIOewTHbHzpBpNGHA7LMRQPn3tsvIOcnEAPX9b7MAWHw5OZt4u%2FxvHdMe%2F472ehg327oPV4nhDN32%2B2DCGojd3dIurlXsJI3MYUSAhniTKjkHFctlRpA4vGlDxRBVhkYtlZ3u0ZhOslg6KkEZgTOa14JDqxTufzhx3WMLW49wLSBZPC1me7fDkN%2FiOnIMhmn2LramMGORLuZAN6rwgWUHMNjqm1XRAPguhXFrwUhkFVmbM3EbfWqYcNuWxbk%2BrzUdA2R%2FLFdZVRWdur8ukSzb76g78I2aPSfQgHpqyhClBCq4KmW58ly2xpkKQz%2FsJTR7JKP7y4R9lEhOU4LMJz%2F6OwFOrMBVSv8xWqbUbZxAEtgfL0NLAFl6ul7Bk3a2AvUwatBUk%2BitM5GcMdQY96taNJFgQ%2FsZweK26V0pg2IHagaHMoGrfZZFP6Dtq7hjlWR9DtwOPdey%2BFRxlSSngHbpNmfU%2BsGeA1hO2MOiM68giUV%2FCLrx79oKI3g6kWTcG5I46D%2FuRiUKRkONECdbm0e0LeE0SK7NOYLFtnVXu9%2FJkOLl2WpM9iJknMFnP1hlHUnr8%2B3EZx0dbk%3D&X-Amz-Algorithm=AWS4-HMAC-SHA256&X-Amz-Date=20191006T203633Z&X-Amz-SignedHeaders=host&X-Amz-Expires=300&X-Amz-Credential=ASIAQ3PHCVTYQYTUV2LE%2F20191006%2Fus-east-1%2Fs3%2Faws4_request&X-Amz-Signature=2f3861aabbbeabe50fc79098937a818cfad37106f7392a9e32447c6c832dfaef&hash=a37a163f23956fad97db69c7e5aa37e3e59b8abe51a8af9b7ae3628597342b64&host=68042c943591013ac2b2430a89b270f6af2c76d8dfd086a07176afe7c76c2c61&pii=S0004951407700570&tid=spdf-6b8d3f9f-652f-4cc5-a622-a79e986c9f47&sid=46e95c8d2154374f799a087289b669675f7bgxrqa&type=client |
| Teasdale CA, Marais BJ, Abrams EJ. HIV: prevention of mother-to-child transmission. BMJ clinical evidence. 2011;2011. https://www.ncbi.nlm.nih.gov/pmc/articles/PMC3217724/pdf/2011-0909.pdf |
| Terry R, Perry R, Ernst E. An overview of systematic reviews of complementary and alternative medicine for fibromyalgia. Clinical rheumatology. 2012 Jan 1;31(1):55-66. https://doi.org/10.1007/s10067-011-1783-5 |
| Theodoratou E, Tzoulaki I, Zgaga L, Ioannidis JP. Vitamin D and multiple health outcomes: umbrella review of systematic reviews and meta-analyses of observational studies and randomised trials. Bmj. 2014 Apr 1;348:g2035. https://doi.org/10.1136/bmj.g2035 |
| Thieme K, Mathys M, Turk DC. Evidenced-based guidelines on the treatment of fibromyalgia patients: Are they consistent and if not, why not? Have effective psychological treatments been overlooked?. The Journal of Pain. 2017 Jul 1;18(7):747-56. https://doi.org/10.1016/j.jpain.2016.12.006 |
| Thornicroft G, Tansella M. Components of a modern mental health service: a pragmatic balance of community and hospital care: overview of systematic evidence. The British journal of psychiatry. 2004 Oct;185(4):283-90. https://doi.org/10.1192/bjp.185.4.283 |
| Thulliez M, Angoulvant D, Pisella PJ, Bejan-Angoulvant T. Overview of systematic reviews and meta-analyses on systemic adverse events associated with intravitreal anti–vascular endothelial growth factor medication use. JAMA ophthalmology. 2018 May 1;136(5):557-66. https://jamanetwork.com/journals/jamaophthalmology/fullarticle/2676023?casa_token=FTmpCkE3XDgAAAAA:cIPlrIZNORXdvsPKKAg_f3q78Tty7V5Uvu2NO_h48_OJe1JeaGt_0b7i33u_uYevNrf9tyt0bcc |
| Ting M, Rice JG, Braid SM, Lee CY, Suzuki JB. Maxillary sinus augmentation for dental implant rehabilitation of the edentulous ridge: a comprehensive overview of systematic reviews. Implant dentistry. 2017 Jun 1;26(3):438-64. https://doi.org/10.1097/ID.0000000000000606 |
| Ting M, Tadepalli NS, Kondaveeti R, Braid SM, Lee CYS, et al. (2018) Intra-Oral Applications of Platelet Concentrates: A Comprehensive Overview of Systematic Reviews. J Interdiscipl Med Dent Sci 6: 233. https://doi.org/10.4172/2376-032X.1000233 |
| Ting M, Tenaglia MS, Jones GH, Suzuki JB. Surgical and patient factors affecting marginal bone levels around dental implants: a comprehensive overview of systematic reviews. Implant dentistry. 2017 Apr 1;26(2):303-15. https://journals.lww.com/implantdent/fulltext/2017/04000/Surgical_and_Patient_Factors_Affecting_Marginal.22.aspx |
| Tol JA, Van Gulik TM, Busch OR, Gouma DJ. Centralization of highly complex low-volume procedures in upper gastrointestinal surgery. A summary of systematic reviews and meta-analyses. Digestive surgery. 2012;29(5):374-83. https://doi.org/10.1159/000343929 |
| Tonkin-Crine SKG, Tan PS, van Hecke O, Wang K, Roberts NW, McCullough A, Hansen MP, Butler CC, Del Mar CB. Clinician-targeted interventions to influence antibiotic prescribing behaviour for acute respiratory infections in primary care: an overview of systematic reviews. Cochrane Database of Systematic Reviews 2017, Issue 9. Art. No.: CD012252. DOI: 10.1002/14651858.CD012252.pub2. https://www.cochranelibrary.com/cdsr/doi/10.1002/14651858.CD012252.pub2/epdf/full |
| Torres, A., Tennant, B., Ribeiro-Lucas, I., Vaux-Bjerke, A., Piercy, K., & Bloodgood, B. (2018). Umbrella and Systematic Review Methodology to Support the 2018 Physical Activity Guidelines Advisory Committee. Journal of Physical Activity and Health, 1–6. sci-hub.tw/10.1123/jpah.2018-0372 |
| Towler P, Molassiotis A, Brearley SG. What is the evidence for the use of acupuncture as an intervention for symptom management in cancer supportive and palliative care: an integrative overview of reviews. Supportive Care in Cancer. 2013 Oct 1;21(10):2913-23. https://doi.org/10.1007/s00520-013-1882-8 |
| Tricco AC, Antony J, Vafaei A, Khan PA, Harrington A, Cogo E, Wilson C, Perrier L, Hui W, Straus SE. Seeking effective interventions to treat complex wounds: an overview of systematic reviews. BMC medicine. 2015 Dec;13(1):89. https://www.ncbi.nlm.nih.gov/pmc/articles/PMC4406332/ |
| Tritschler T, Kraaijpoel N, Le Gal G, Wells PS. Venous thromboembolism: advances in diagnosis and treatment. Jama. 2018 Oct 16;320(15):1583-94. https://jamanetwork.com/journals/jama/fullarticle/2707465?casa_token=3E9yHGbEavIAAAAA%3aJdwWb5l_LxGmcI0HNIXvlvaNZrh3OEAPfSjUo7_zJIVDjMLEScRjVA_-YMPdpVs_1ab56bv0LQ8 |
| Ubbink DT, Santema TB, Stoekenbroek RM. Systemic wound care: a meta-review of cochrane systematic reviews. Surg Technol Int. 2014 Mar;24(99):111. https://pure.uva.nl/ws/files/11863111/02.pdf |
| Uslar T, Anabalón J. Is percutaneous closure of the left atrial appendage comparable to anticoagulants for atrial fibrillation?. Medwave. 2015 Aug 17;15(Suppl 2):e6218. https://www.medwave.cl/link.cgi/English/Updates/Epistemonikos/6219?ver=sindiseno |
| Van Achterberg T, Huisman-de Waal GG, Ketelaar NA, Oostendorp RA, Jacobs JE, Wollersheim HC. How to promote healthy behaviours in patients? An overview of evidence for behaviour change techniques. Health promotion international. 2011 Jun 1;26(2):148-62. |
| van de Griendt EJ, Tuut MK, de Groot H, Brand PL. Applicability of evidence from previous systematic reviews on immunotherapy in current practice of childhood asthma treatment: a GRADE (Grading of Recommendations Assessment, Development and Evaluation) systematic review. BMJ open. 2017 Dec 1;7(12):e016326. http://doi.org/10.1136/bmjopen-2017-016326 |
| Van de Laar FA, Akkermans RP, Van Binsbergen JJ. Limited evidence for effects of diet for type 2 diabetes from systematic reviews. European journal of clinical nutrition. 2007 Aug;61(8):929. https://doi.org/10.1038/sj.ejcn.1602611 |
| Van der Feltz-Cornelis CM, Sarchiapone M, Postuvan V, Volker D, Roskar S, Grum AT, Carli V, McDaid D, O’Connor R, Maxwell M, Ibelshäuser A. Best practice elements of multilevel suicide prevention strategies. Crisis. 2011 Sep 26; 32(6), pp. 319-333. http://doi.org/10.1027/0227-5910/a000109 |
| Van Der Heijden, E.H., Casal, R.F., Trisolini, R., Steinfort, D.P., Hwangbo, B., Nakajima, T., Guldhammer-Skov, B., Rossi, G., Ferretti, M., Herth, F.F. and Yung, R., 2014. Guideline for the acquisition and preparation of conventional and endobronchial ultrasound-guided transbronchial needle aspiration specimens for the diagnosis and molecular testing of patients with known or suspected lung cancer. Respiration, 88(6), pp.500-517. https://www.karger.com/Article/PDF/368857 |
| Van der Weijden FA, Slot DE. Efficacy of homecare regimens for mechanical plaque removal in managing gingivitis a meta review. Journal of clinical periodontology. 2015 Apr;42:S77-91. https://onlinelibrary.wiley.com/doi/pdf/10.1111/jcpe.12359 |
| Van der Wouden JC, Bueving HJ, Poole P. Preventing influenza: an overview of systematic reviews. Respiratory medicine. 2005 Nov 1;99(11):1341-9. https://doi.org/10.1016/j.rmed.2005.07.001 |
| van Sluijs EM, Kriemler S, McMinn AM. The effect of community and family interventions on young people's physical activity levels: a review of reviews and updated systematic review. British journal of sports medicine. 2011 Sep 1;45(11):914-22. https://www.ncbi.nlm.nih.gov/pmc/articles/PMC3736309/ |
| van Tulder MW, Koes B, Seitsalo S, Malmivaara A. Outcome of invasive treatment modalities on back pain and sciatica: an evidence-based review. European Spine Journal. 2006 Jan 1;15(1):S82-92. https://dx.doi.org/10.1007%2Fs00586-005-1049-5 |
| Veerman SR, Schulte PF, de Haan L. Treatment for negative symptoms in schizophrenia: a comprehensive review. Drugs. 2017 Sep 1;77(13):1423-59. https://www.ncbi.nlm.nih.gov/pubmed/28776162 |
| Vélez-Díaz-Pallarés M, Lozano-Montoya I, Abraha I, Cherubini A, Soiza RL, O'Mahony D, Montero-Errasquín B, Cruz-Jentoft AJ. Nonpharmacologic interventions to heal pressure ulcers in older patients: an overview of systematic reviews (the SENATOR-ONTOP series). Journal of the American Medical Directors Association. 2015 Jun 1;16(6):448-69. https://pdf.sciencedirectassets.com/273309/1-s2.0-S1525861014X00071/1-s2.0-S1525861015000845/main.pdf?X-Amz-Security-Token=IQoJb3JpZ2luX2VjEP3%2F%2F%2F%2F%2F%2F%2F%2F%2F%2FwEaCXVzLWVhc3QtMSJHMEUCIGN%2Fj7%2FDNrhwuaqzU0y4kBTlnpFLekupmFjCeNttH0HjAiEAgbph4cG0b947SbrrAOVDUYJ7n4U84JK69qK%2BN8LKr1IqvQMI9v%2F%2F%2F%2F%2F%2F%2F%2F%2F%2FARACGgwwNTkwMDM1NDY4NjUiDPw%2Ffre4sjX3hJpP7yqRAzpMO1nTcgSJwuAlP19wcN9NGt6qRtXAE026LHvWappMvvuTPxao9Bu4kMYHscVxSEKPOh8qyPNYNujHnM20Tl1WWZb6JyKC3g271dRUpX%2FMmOjTYUVnEwu%2FU5FJ1N4ygnlUGRSYQZR9Hqxr8KTp4xJs5CgwL8S1SrbvfwmmuDwI2wpN9Nb4jqu4%2BQGwbm85Kr8R91Dqt5s2ovZit5fdIm0Z7rQhD5IxuiFOdY985827lju0LxmLiRJKxU4cmYNIvu8kQDR%2FHhnolV%2FQupIgSPO3iwLMf1zrrf1DchE5iKh4Y269aI9YydPfha1WMZa96ZxBujT01xXdfhh0FDxUMYxHLyVluiEYyOsidIZzojmNPaoMd8vMzQs3WF6366B9eJd6feO5CZ5l1kRDL0DABDShWImjLqy6EW4oWVfcxGIXz92YgEfX67UwSeJ93dZt%2BUYJxbFD1t1WqVv6dHJNbx9osalydd9XTpCEQJHD2Cf3GJiK1gtUCPeplAWmKhyzwkOPPPD742lEbrWmutJSXotYMIKxifQFOusBym2vyLHd5nd71c4apLHltSGc1H6OAz84h4pf3YsknfMnNITj19Ui%2FlTwc4hX5yYs8j9g%2F49jfxdz9bfGre8Lsf1Spnvyzq0JUJtH7ppczDNudLltrlZ6x7iFTKC8TzGB4WHPJuHlyxPq2%2Byb6DJnR%2F6UrWoDkxsYXPBZk9Dj3rSHAIgubikr6XhMULRlScZfml9F2dgycByqxY7pi4a2Y8Aq8j8X4U9qoClahTIR%2FsQ2IiaIR%2F8uIj5oY9oBaquODC2%2Ba8JnsiGSeEM4%2Bdm%2FtGsWApbb0ATrLPG%2BEoG%2Fz9SmiiZzo8EymmsA5A%3D%3D&X-Amz-Algorithm=AWS4-HMAC-SHA256&X-Amz-Date=20200330T212751Z&X-Amz-SignedHeaders=host&X-Amz-Expires=300&X-Amz-Credential=ASIAQ3PHCVTYRIDX2CAC%2F20200330%2Fus-east-1%2Fs3%2Faws4_request&X-Amz-Signature=9fe519bdd1dd2c9596c23c1feff75924379ffbedb1f1bca2e4035a269273be9d&hash=ab0876ee19cdcf988af22d5c8e42f090c351268448f1fdc643d91880cff004bd&host=68042c943591013ac2b2430a89b270f6af2c76d8dfd086a07176afe7c76c2c61&pii=S1525861015000845&tid=spdf-c6fc40ba-150e-4a53-a154-851dd8e5aea4&sid=96f70fdf68a65245ec6a1304ea3a8bf63484gxrqa&type=client |
| Veronese N, Solmi M, Caruso MG, Giannelli G, Osella AR, Evangelou E, Maggi S, Fontana L, Stubbs B, Tzoulaki I. Dietary fiber and health outcomes: an umbrella review of systematic reviews and meta-analyses. The American journal of clinical nutrition. 2018 Mar 1;107(3):436-44. https://academic.oup.com/ajcn/article/107/3/436/4939351 |
| Vidal L, Gafter-Gvili A, Shpilberg O. Immunotherapy for patients with follicular lymphoma: the contribution of systematic reviews. Acta haematologica. 2011;125(1-2):23-31. https://doi.org/10.1159/000318883 |
| Vieira A, Reis AM, Matos LC, Machado J, Moreira A. Does auriculotherapy have therapeutic effectiveness? An overview of systematic reviews. Complementary therapies in clinical practice. 2018 Nov 1;33:61-70. https://doi.org/10.1016/j.ctcp.2018.08.005 |
| Vieira de Melo BB, Trigueiro MJ, Rodrigues PP. Systematic overview of neuroanatomical differences in ADHD: Definitive evidence. Developmental neuropsychology. 2018 Jan 2;43(1):52-68. https://www.tandfonline.com/doi/full/10.1080/87565641.2017.1414821 |
| Vilela VC, Pacheco RL, Latorraca CO, Pachito DV, Riera R. What do Cochrane systematic reviews say about non-pharmacological interventions for treating cognitive decline and dementia?. Sao Paulo Medical Journal. 2017 Jun;135(3):309-20. http://www.scielo.br/pdf/spmj/v135n3/1806-9460-spmj-135-03-00309.pdf |
| Visser J, McLachlan MH, Maayan N, Garner P. Community-based supplementary feeding for food insecure, vulnerable and malnourished populations - an overview of systematic reviews. Cochrane Database of Systematic Reviews 2018, Issue 11. Art. No.: CD010578. DOI: 10.1002/14651858.CD010578.pub2. https://www.cochranelibrary.com/cdsr/doi/10.1002/14651858.CD010578.pub2/full |
| Vivares-Builes AM, Rangel-Rincón LJ, Botero JE, Agudelo-Suárez AA. Gaps in knowledge about the association between maternal periodontitis and adverse obstetric outcomes: an umbrella review. Journal of Evidence Based Dental Practice. 2018 Mar 1;18(1):1-27. |
| Wagner SL, Koehn C, White MI, et al. Mental health interventions in the workplace and work outcomes: A best-evidence synthesis of systematic reviews. Int J Occup Environ Med 2016;7:1-14. https://doi.org/10.15171/ijoem.2016.607 |
| Walsh NE, Pearson J, Healey EL. Physiotherapy management of lower limb osteoarthritis. British medical bulletin. 2017 May 3;122(1):151-61. https://doi.org/10.1093/bmb/ldx012 |
| Walter JE, Lovatsis D, Easton W, Epp A, Farrell SA, Girouard L, Gupta CK, Harvey MA, Larochelle A, Robert M, Ross S. Transvaginal mesh procedures for pelvic organ prolapse. Journal of Obstetrics and Gynaecology Canada. 2011 Feb 1;33(2):168-74. https://www.sciencedirect.com/science/article/abs/pii/S1701216316348046 |
| Warburton DE, Bredin SS. Health benefits of physical activity: a systematic review of current systematic reviews. Current opinion in cardiology. 2017 Sep 1;32(5):541-56. https://doi.org/10.1097/HCO.0000000000000437 |
| Weare K, Nind M. Mental health promotion and problem prevention in schools: what does the evidence say?. Health promotion international. 2011 Dec 1;26(suppl_1):i29-69. https://doi.org/10.1093/heapro/dar075 |
| Wells C, Kolt GS, Marshall P, Hill B, Bialocerkowski A. Effectiveness of Pilates exercise in treating people with chronic low back pain: a systematic review of systematic reviews. BMC medical research methodology. 2013 Dec;13(1):7. |
| Welsh EJ, Evans DJ, Fowler SJ, Spencer S. Interventions for bronchiectasis: an overview of Cochrane systematic reviews. Cochrane Database of Systematic Reviews. 2015(7). https://doi.org/10.1002/14651858.CD010337.pub2 |
| West R, McNeill A, Raw M. Smoking cessation guidelines for health professionals: an update. Thorax. 2000 Dec 1;55(12):987-99. http://dx.doi.org/10.1136/thorax.55.12.987 |
| White AP, Arnold PM, Norvell DC, Ecker E, Fehlings MG. Pharmacologic management of chronic low back pain: synthesis of the evidence. Spine. 2011 Oct 1;36:S131-43. https://doi.org/10.1097/BRS.0b013e31822f178f |
| Wiffen PJ, Derry S, Moore RA, Aldington D, Cole P, Rice AS, Lunn MP, Hamunen K, Haanpaa M, Kalso EA. Antiepileptic drugs for neuropathic pain and fibromyalgia‐an overview of Cochrane reviews. Cochrane Database of Systematic Reviews. 2013(11). https://www.ncbi.nlm.nih.gov/pmc/articles/PMC6469538/ |
| Wiffen PJ, Wee B, Derry S, Bell RF, Moore RA. Opioids for cancer pain‐an overview of Cochrane reviews. Cochrane Database of Systematic Reviews. 2017(7). https://doi.org/10.1002/14651858.CD012592.pub2 |
| Wilby J, Kainth A, Hawkins N, et al. Clinical effectiveness, tolerability and cost-effectiveness of newer drugs for epilepsy in adults: a systematic review and economic evaluation. Health Technol Assess. 2005;9(15):1-iv. https://doi.org/10.3310/hta9150 |
| Williams G, Craig JC. Prevention of recurrent urinary tract infection in children. Current opinion in infectious diseases. 2009 Feb 1;22(1):72-6. https://doi.org/10.1097/QCO.0b013e328320a885 |
| Williams Jr JW, Ranney L, Morgan LC, Whitener L. How reviews covered the unfolding scientific story of gabapentin for bipolar disorder. General hospital psychiatry. 2009 May 1;31(3):279-87. https://doi.org/10.1016/j.genhosppsych.2009.02.006 |
| Winkelmann A, Bork H, Brückle W, Dexl C, Heldmann P, Henningsen P, Krumbein L, Pullwitt V, Schiltenwolf M, Häuser W. Physiotherapie, Ergotherapie und physikalische Verfahren beim Fibromyalgiesyndrom. Der Schmerz. 2017 Jun 1;31(3):255-65. |
| Wisanskoonwong P, Fahy K, Hastie C. The effectiveness of medical interventions aimed at preventing preterm birth: A literature review. Women and Birth. 2011 Dec 1;24(4):141-7. https://pdf.sciencedirectassets.com/273543/1-s2.0-S1871519211X00069/1-s2.0-S1871519210000855/main.pdf?X-Amz-Security-Token=IQoJb3JpZ2luX2VjEGUaCXVzLWVhc3QtMSJHMEUCIELUP%2BYaquiAWttoSIGc%2Bm%2FRgf6mFjS76e7sVRvWiN2HAiEAr%2BGIkMEahpfEoq%2B%2Ba0hE6dQdXTTmwacJXUenRa0zdjQqvQMI%2Fv%2F%2F%2F%2F%2F%2F%2F%2F%2F%2FARACGgwwNTkwMDM1NDY4NjUiDMcbl3YeE1cdajmTwCqRAyubWha6%2FFiqgvW4uWGvzsKLUfRhV%2BDLkKbc7WGDLF1L93%2FmUNXuLlZES5g9X1kMkrylE2oEicsQfsRg8SyJGdHem6p4aMxP3%2BI4rX1bWL1LfhPBMkvUf65NKIst59Voe1j23ZVLQYw8KpF9HxD3NajHiI8B%2BFPPYEdUkgVk3tfeOb783UY8cuL5ATK64DtljQ8wO885px%2FwmarJGHGNxZUfCG1dp4T187TAAUpY6rAjyz9pLBVV66JLH0oVxBY3l7LRe7ceY0F1Vt9vf0X9lAc5xcIBaAqbKoDXUIP%2BLUB4hFpcLDibz2Z2%2B2kff5yRC1dBHV2WdOGBa%2Fm7yWS4ZYXM5NowOaQ2E9JNW4PMoRUUsyhvRqX434v5EXnlSDKMMJPVOSp%2BsjkAeBhO6cMo9AZ4lzHKZpjO%2FpNTDnR2JHqqTWLWTKrlPceskBPtevAmGhiT4rOdkM30t61tIB7m35e9OHOqcSbX2KxPODDeDo0v01PqRHDWXZ0%2B62Gf%2B4hsOv8u9LyJGCDGyaGFEbyhzZW6MNPozvEFOusBBg6ysc%2FGjyYA0x1OYfTnFAGHjaIbeRj%2B0h35FkjSf2mvl9NbT0RxM3YdSUOKxXu10UmWToKnYJDLLn1pVSJV3aT5cqTPzjAMB%2BMMXo80MWsz64ZzLnEORhnRHvw4u%2Fc9RCJPWl5CjNFnX%2Bvj6f%2BFLVz0OuvbTuxUhUj%2BwGfHGd6skVAZM0XUXmtHHL1Inp0COZMwRuClW6bk%2Bj05FhPVD%2Fxb%2Fao%2BiL3w3pDiqFo84OnX%2FikPcEzLSNz3qCA3iG3Yo2VOOXY4giTOUeN4hrYQJL2i%2ForjTgYoxFr57rlvzRzMv0q1YOjH0qFilg%3D%3D&X-Amz-Algorithm=AWS4-HMAC-SHA256&X-Amz-Date=20200131T053001Z&X-Amz-SignedHeaders=host&X-Amz-Expires=300&X-Amz-Credential=ASIAQ3PHCVTYS5JGD6PJ%2F20200131%2Fus-east-1%2Fs3%2Faws4_request&X-Amz-Signature=90dde5afb729ce8019144294642a35a4c6894e5324fe99c322dd2cf44ab2d24a&hash=82de8683dbab2519f09b603c6c220fc3a6c639bb14bbd20b7af032697098d286&host=68042c943591013ac2b2430a89b270f6af2c76d8dfd086a07176afe7c76c2c61&pii=S1871519210000855&tid=spdf-706cc5fa-6c4d-4a2d-8602-05eb53495d09&sid=ab1ceb4e2494434c11390e89012b933cfdefgxrqa&type=client |
| Wiysonge CS, Paulsen E, Lewin S, Ciapponi A, Herrera CA, Opiyo N, Pantoja T, Rada G, Oxman AD. Financial arrangements for health systems in low‐income countries: an overview of systematic reviews. Cochrane Database of Systematic Reviews. 2017(9). https://www.cochranelibrary.com/cdsr/doi/10.1002/14651858.CD011084.pub2/abstract |
| Wolfenden L, Campbell E, Wiggers J, Walsh RA, Bailey LJ. Helping hospital patients quit: what the evidence supports and what guidelines recommend. Preventive medicine. 2008 Apr 1;46(4):346-57. https://doi.org/10.1016/j.ypmed.2007.12.003 |
| Wolfenden L, Wiggers J, d'Espaignet ET, Bell AC. How useful are systematic reviews of child obesity interventions?. obesity reviews. 2010 Feb 1;11(2):159-65. https://doi.org/10.1111/j.1467-789X.2009.00637.x |
| World Health Organization. WHO position paper on mammography screening. World Health Organization; 2014. https://apps.who.int/iris/bitstream/handle/10665/137339/9789241507936_eng.pdf |
| World Health Organization. WHO recommendations for prevention and treatment of maternal peripartum infections. World Health Organization; 2015. https://www.who.int/reproductivehealth/publications/maternal_perinatal_health/peripartum-infections-guidelines/en/ |
| World Health Organization. WHO recommendations on interventions to improve preterm birth outcomes; 2015. https://www.who.int/reproductivehealth/publications/maternal_perinatal_health/preterm-birth-guideline/en/ |
| Worrall G. Herpes labialis. BMJ Clin Evid. 2009;1704. PMID: 21726482 |
| Worswick J, Wayne SC, Bennett R, Fiander M, Mayhew A, Weir MC, Sullivan KJ, Grimshaw JM. Improving quality of care for persons with diabetes: an overview of systematic reviews-what does the evidence tell us?. Systematic reviews. 2013 Dec;2(1):26. https://doi.org/10.1186/2046-4053-2-26 |
| Wu L, Norman G, Dumville JC, O’Meara S, Bell-Syer SEM. Dressings for treating foot ulcers in people with diabetes: an overview of systematic reviews. Cochrane Database of Systematic Reviews 2015, Issue 7. Art. No.: CD010471. DOI: 10.1002/14651858.CD010471.pub2. https://www.cochranelibrary.com/cdsr/doi/10.1002/14651858.CD010471.pub2/full |
| Wu X, Chung VC, Hui EP, Ziea ET, Ng BF, Ho RS, Tsoi KK, Won g SY, Wu JC. Effectiveness of acupuncture and related therapies for palliative care of cancer: overview of systematic reviews. Scientific reports. 2015 Nov 26;5:16776. https://www.ncbi.nlm.nih.gov/pmc/articles/PMC4660374/ |
| Wu X, Tian W, Kubilay NZ, Ren J, Li J. Is it necessary to place prophylactically an abdominal drain to prevent surgical site infection in abdominal operations? A systematic meta-review. Surgical infections. 2016 Dec 1;17(6):730-8. https://doi.org/10.1089/sur.2016.082 |
| Wuttke C, Mangnus E, Kumar S. Manualtherapeutische Interventionen bei Kopfschmerzerkrankungen. manuelletherapie. 2013 May;17(02):88-93. https://doi.org/10.1055/s-0033-1346994 |
| Xin Z, Xue-Ting L, De-Ying K. GRADE in systematic reviews of acupuncture for stroke rehabilitation: recommendations based on high-quality evidence. Scientific reports. 2015 Nov 12;5:16582. https://www.nature.com/articles/srep16582 |
| Xing D, Wang B, Zhang W, Yang Z, Hou Y, Chen Y, Lin J. Intra‐articular platelet‐rich plasma injections for knee osteoarthritis: An overview of systematic reviews and risk of bias considerations. International journal of rheumatic diseases. 2017 Nov;20(11):1612-30. https://doi.org/10.1111/1756-185X.13233 |
| Xing D, Wang Q, Yang Z, Hou Y, Zhang W, Chen Y, Lin J. Mesenchymal stem cells injections for knee osteoarthritis: a systematic overview. Rheumatology international. 2018 Aug 1;38(8):1399-411. https://doi.org/10.1007/s00296-017-3906-z |
| Xu J, Lombardi G, Jiao W, Banfi G. Effects of exercise on bone status in female subjects, from young girls to postmenopausal women: an overview of systematic reviews and meta-analyses. Sports Medicine. 2016 Aug 1;46(8):1165-82. https://link.springer.com/article/10.1007/s40279-016-0494-0 |
| Xu X, Mishra GD, Jones M. Evidence on multimorbidity from definition to intervention: an overview of systematic reviews. Ageing research reviews. 2017 Aug 1;37:53-68. https://doi.org/10.1016/j.arr.2017.05.003 |
| Yamada J, Stinson J, Lamba J, Dickson A, McGrath PJ, Stevens B. A review of systematic reviews on pain interventions in hospitalized infants. Pain Research and Management. 2008;13(5):413-20. http://dx.doi.org/10.1155/2008/232316 |
| Yang C, Hao Z, Zhang LL, Guo Q. Efficacy and safety of acupuncture in children: an overview of systematic reviews. Pediatric research. 2015 Aug;78(2):112-9. https://www.nature.com/articles/pr201591.pdf?origin=ppub |
| Yang C, Hao Z, Zhu C, Guo Q, Mu D, Zhang L. Interventions for tic disorders: an overview of systematic reviews and meta analyses. Neuroscience & Biobehavioral Reviews. 2016 Apr 1;63:239-55. https://doi.org/10.1016/j.neubiorev.2015.12.013 |
| Yang M, Jiang L, Wang Q, Chen H, Xu G. Traditional Chinese medicine for knee osteoarthritis: An overview of systematic review. PloS one. 2017;12(12). https://www.ncbi.nlm.nih.gov/pmc/articles/PMC5739454/ |
| Yang S, Zhang R, Zhu Q, Wang G, Ding X, Wang J. Evaluation of surgical and non-surgical interventions for clavicle fractures. Acta orthop traumatol turc. 2014 Jan 1;48(3):253-8. http://www.aott.org.tr/Content/files/sayilar/398/398-3991.pdf |
| YANG Yan-tao;MA Li;YANG Xiao-xia;ZHAO Chun-xiang. Traditional Chinese Medicine for Essential Hypertension:An Overview of Systematic Reviews. Journal of Chinese Evidence-Based Medicine , Chinese Journal of Evidence-Based Medicine 9; 2014. |
| Yi J, Xiao J, Li H, Li Y, Li X, Zhao Z. Effectiveness of adjunctive interventions for accelerating orthodontic tooth movement: a systematic review of systematic reviews. Journal of oral rehabilitation. 2017 Aug;44(8):636-54. https://doi.org/10.1111/joor.12509 |
| Yiu KC, Rohwer A, Young T. Integration of care for hypertension and diabetes: a scoping review assessing the evidence from systematic reviews and evaluating reporting. BMC Health Services Research. 2018 Dec;18(1):481. https://doi.org/10.1186/s12913-018-3290-8 |
| Young SM, Bansal P, Vella ET, Finelli A, Levitt C, Loblaw A. Guideline for referral of patients with suspected prostate cancer by family physicians and other primary care providers. Canadian Family Physician. 2015 Jan 1;61(1):33-9. https://www.cfp.ca/content/cfp/61/1/33.full.pdf |
| Yount KM, Krause KH, Miedema SS. Preventing gender-based violence victimization in adolescent girls in lower-income countries: Systematic review of reviews. Social Science & Medicine. 2017 Nov 1;192:1-3. https://doi.org/10.1016/j.socscimed.2017.08.038 |
| Yuan Q, Wang CW, Shi J, Lin ZX. Effects of Ginkgo biloba on dementia: An overview of systematic reviews. Journal of ethnopharmacology. 2017 Jan 4;195:1-9. https://doi.org/10.1016/j.jep.2016.12.005 |
| Zhang F, Shen A, Jin Y, Qiang W. The management strategies of cancer-associated anorexia: a critical appraisal of systematic reviews. BMC complementary and alternative medicine. 2018 Dec;18(1):236. https://doi.org/10.1186/s12906-018-2304-8 |
| Zhang JH, Wang D, Liu M. Overview of systematic reviews and meta-analyses of acupuncture for stroke. Neuroepidemiology 2014;42(1):50-8. https://www.karger.com/Article/PDF/355435 |
| Zhang W, Moskowitz RW, Nuki G, Abramson S, Altman RD, Arden N, Bierma-Zeinstra S, Brandt KD, Croft P, Doherty M, Dougados M. OARSI recommendations for the management of hip and knee osteoarthritis, part I: critical appraisal of existing treatment guidelines and systematic review of current research evidence. Osteoarthritis and cartilage. 2007 Sep 1;15(9):981-1000. https://www.sciencedirect.com/science/article/pii/S1063458407002348 |
| Zhang W, Moskowitz RW, Nuki G, Abramson S, Altman RD, Arden N, Bierma-Zeinstra S, Brandt KD, Croft P, Doherty M, Dougados M. OARSI recommendations for the management of hip and knee osteoarthritis, Part II: OARSI evidence-based, expert consensus guidelines. Osteoarthritis and cartilage. 2008 Feb 1;16(2):137-62. https://www.sciencedirect.com/science/article/pii/S1063458407003974 |
| Zhang W, Nuki G, Moskowitz RW, Abramson S, Altman RD, Arden NK, Bierma-Zeinstra S, Brandt KD, Croft P, Doherty M, Dougados M. OARSI recommendations for the management of hip and knee osteoarthritis: part III: Changes in evidence following systematic cumulative update of research published through January 2009. Osteoarthritis and cartilage. 2010 Apr 1;18(4):476-99. https://www.sciencedirect.com/science/article/pii/S1063458410000464 |
| Zhang X, Liu XT, Kang DY. Traditional Chinese patent medicine for acute ischemic stroke: An overview of systematic reviews based on the GRADE approach. Medicine. 2016 Mar;95(12). https://www.ncbi.nlm.nih.gov/pmc/articles/PMC4998369/ |
| Zhao XF, Du Y, Liu PG, Wang S. Acupuncture for stroke: evidence of effectiveness, safety, and cost from systematic reviews. Topics in stroke rehabilitation. 2012 May 1;19(3):226-33. https://doi.org/10.1310/tsr1903-226 |
| Zheng X, Sun Y, Zhang Y, Cai T, Sun F, Lin J. Implants for orthodontic anchorage: An overview. Medicine. 2018 Mar;97(13). https://dx.doi.org/10.1097%2FMD.0000000000010232 |
| Zubala A, MacGillivray S, Frost H, Kroll T, Skelton DA, Gavine A, Gray NM, Toma M, Morris J. Promotion of physical activity interventions for community dwelling older adults: a systematic review of reviews. PloS one. 2017 Jul 10;12(7):e0180902. https://doi.org/10.1371/journal.pone.0180902 |
| Zwicker JG, Mayson TA. Effectiveness of treadmill training in children with motor impairments: an overview of systematic reviews. Pediatric Physical Therapy. 2010 Dec 1;22(4):361-77. https://doi.org/10.1097/PEP.0b013e3181f92e54 |
